# Homology Modeling of Type-P5 ATPases from the Malaria Parasite: Insight into Their Functions and Evolution, and Implications About the Effect and Role of Intrinsically Disordered Protein Structure

**DOI:** 10.3390/pathogens14111164

**Published:** 2025-11-14

**Authors:** Mark F. Wiser

**Affiliations:** Department of Tropical Medicine and Infectious Disease, Celia Scott Weatherhead School of Public Health & Tropical Medicine, Tulane University, New Orleans, LA 70112, USA; wiser@tulane.edu; Tel.: +1-504-988-2507

**Keywords:** P-type ATPase, type-P5 ATPase, malaria parasite, *Plasmodium*, Haemosporida, homology modeling, intrinsically disordered proteins, Swiss Model, AlphaFold

## Abstract

Type-P5 ATPases are the least characterized among the P-type ATPases and this is especially true in the case of the malaria parasite. In this study, Spf1, a subtype-P5A ATPase of yeast, and ATP13A2, a subtype-P5B ATPase of humans, were used as templates to extensively characterize the sequences and structural features of haemosporidian type-P5 ATPases. Malaria parasites have both subtype-P5A and subtype-P5B ATPase genes and the structural features of the proteins recapitulate the known structures of subtype-P5A and subtype-P5B ATPases. Detailed structural analysis detected an additional α-helix in the P-domain of subtype-P5A ATPases, which is not found in subtype-P5B ATPases. This feature may be an additional signature to distinguish subtype-P5A and subtype-P5B ATPases, in addition to the previously described differences in the membrane loops of the N-terminal domain, the arm in the P-domain of subtype-P5A, and substrate differences. A notable difference in the type-P5 ATPases from the malaria parasite, as compared to the templates, is the insertion of multiple variable and low-complexity regions that form intrinsically disorganized loops. These loops may form a shroud-like structure that protects the core ATPase structure and/or participates in low-affinity interprotein interactions. Homology modeling did not provide definitive answers about the substrate specificity of the haemosporidian type-P5 ATPases. However, the haemosporidian subtype-P5A ATPase is likely an ER transmembrane dislocase as are the other subtype-P5A ATPases. In contrast, the subtype-P5B ATPases of the malaria parasite are not likely to be polyamine transporters in lysosomes, as have been described in fungi and metazoans. This suggests that subtype-P5B ATPases have undergone lineage-specific divergence in regard to their function(s).

## 1. Introduction

P-type ATPases move substances—especially cations and lipids—across membranes through the hydrolysis of ATP and the transient phosphorylation of a highly conserved aspartate (D) residue [1]. Despite their crucial importance in cellular physiology, relatively little research has been conducted on the potential of P-type ATPases as drug targets against the malaria parasite. Among the potential twelve P-type ATPases that have been identified in the *Plasmodium falciparum* genome [2], most research has been conducted on the SERCA orthologue, and potential drugs that target this enzyme have been identified [3,4]. The ability to generate accurate three-dimensional models of proteins using known structures as templates, often called homology modeling, will certainly expedite the identification and characterization of potential drug targets [5]. This may be especially true of P-type ATPases, since their three-dimensional structures are relatively well characterized. Homology modeling is a powerful and essential tool in pathogen research and drug discovery that will provide insight into protein structure, function, and interactions.

### 1.1. P-Type ATPases

P-type ATPases comprise a large and ubiquitous gene family that is defined by canonical structural features. The first and foremost is the highly conserved phosphorylation motif DKTGT which defines P-type ATPases. During substrate transport the aspartate (D) is transiently phosphorylated via ATP hydrolysis and the resulting protein conformation changes associated with the phosphorylation-dephosphorylation cycles facilitate movement of substances across membranes. All P-type ATPases comprise four highly conserved domains called the actuator (A) domain, the phosphorylation (P) domain, the nucleotide-binding (N) domain, and membrane (M) domain [6]. The A-domain has phosphatase activity to remove the phosphate. The N-domain binds ATP so that the γ-phosphate is adjacent to the DKTGT motif in the P-domain. These three domains are located on the cytoplasmic face of the membrane. The M-domain is formed by six core transmembrane helices (cTM) and a variable number of supporting transmembrane helices (sTM). A substrate-binding groove that opens to the extracytoplasmic (i.e., extracellular or luminal) side of the membrane is formed by the cTM. There is a highly conserved proline in the fourth transmembrane helix (cTM4) that forms a ‘kink’ at the base of the substrate binding groove.

**Figure 1:**
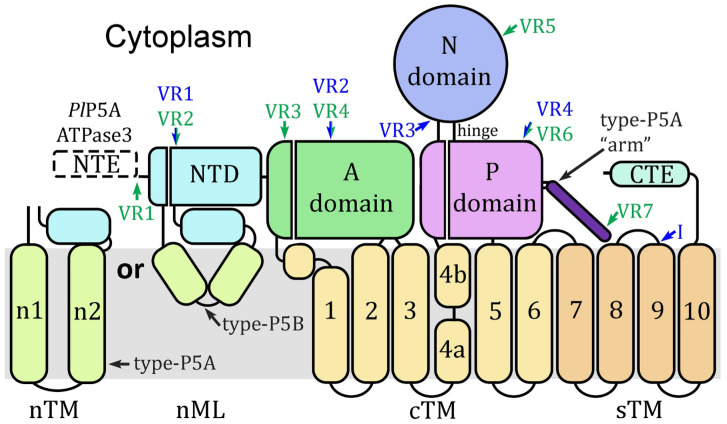
Schematic representation of type-P5 ATPases. The canonical A-domain, N-domain, and P-domain are located on the cytoplasmic face of the membrane along with an N-terminal domain (NTD) and a C-terminal extension (CTE) of variable length. The M-domain (gray background) consists of six core transmembrane (cTM) helices, four supporting transmembrane (sTM) helices, and membrane helices associated with the NTD. Subtype-P5A ATPases have two N-terminal transmembrane helices (nTM) and subtype-P5B has an N-terminal membrane loop (nML) formed by three short helices. Subtype-P5A ATPases have a helical projection from the P-domain that is not found in subtype-P5B ATPases, called the “arm”. ATP binding occurs in a crevice between the N-domain and P-domain called the hinge. The transmembrane helix of cTM4 is disrupted by conserved prolines that results in a ‘kink’ in the helix (between 4a and 4b), which plays an essential role in the formation of the substrate-binding groove. Similar nomenclature and color schemes are used in all subsequent figures. A single subtype-P5A ATPase (*Pl*P5A) and two subtype-P5B ATPases (ATPase1 and ATPase3) have been identified in genomes of malaria parasites. *Pl*P5A has a single exon and ATPase1 and ATPase3 have two exons with the position of the intron denoted by a blue arrow near sTM9. *Pl*P5A and ATPase3 have N-terminal extensions that are not found in other type-P5 ATPases. Alignment of *Pl*P5A sequences reveals seven variable regions (VR1-7 green) composed of low-complexity sequences, which are denoted with green arrows and lettering. ATPase1 and ATPase3 exhibit four variable regions (VR1-4), three of which are shared with *Pl*P5A, composed of a low-complexity sequence, and are denoted with blue arrows and lettering.

There are five major types (numbered 1–5) of P-type ATPases based on sequence homology, additional structural domains, and substrate specificity [1,7]. Subtypes have also been defined and are designated with capital letters. Type-P5 ATPases, the least characterized among the P-type ATPases, are defined through sequence homology and structural features. For example, the signature motif is an expanded FDKTGTLT, there is an N-terminal domain (NTD), and the M-domain contains four sTM helices (Figure 1). The two subtypes (A and B) of type-P5 ATPases are quite similar and differ primarily in the NTD, the P-domain, and substrate specificity [1,8]. For example, the NTD of subtype-P5A has two N-terminal transmembrane helices (nTM), whereas subtype-P5B has a triangular N-terminal membrane loop (nML). Another distinguishing feature in subtype-P5A is a helical projection from the P-domain, called the ‘arm’, that is not found in subtype-P5B. And subtype-P5A ATPase has been described as a transmembrane helix dislocase of the ER [9] and subtype-P5B ATPase has been described as a polyamine transporter of the lysosome [10,11].

### 1.2. Type-P5 ATPases of Plasmodium

Type-P5 ATPases are only found in eukaryotes, and most eukaryotes have a single copy of the subtype-P5A and multiple paralogues of the subtype-P5B, especially in multicellular organisms [8,12]. A single gene for a P5A-subtype has been identified in *Plasmodium* [12]. The gene has not been characterized other than its presence in genomic databases and a putative description as a cation-transporting P-type ATPase that is located on chromosome seven [2]. Two subtype-P5B genes, designated as ATPase1 and ATPase3, have been identified in *P. falciparum*. ATPase1 arose from a duplication of ATPase3 early in the evolution of the malaria parasite and is only found in *P. falciparum* and related parasites of the great apes (i.e., *Laverania*), avian malaria parasites, and *Haemoproteus* [13]. Orthologues of ATPase3 can be positively identified throughout the Apicomplexa, but no clear orthologues outside of the Apicomplexa could be identified. ATPase1 and ATPase3 from all the apicomplexan species exhibited a high level of sequence homology except for four rather large variable regions composed of low-complexity sequences. Both ATPase1 and ATPase3 genes also have a single intron located in the same position. The only quasi-substantial difference between these two paralogues is an extended N-terminus of variable sequence in ATPase3.

Homology modeling with Phyre^®^ was carried out in the previous study [13] to search for structural homologues. Numerous P-type ATPases were identified as potential templates and templates from type-P5 ATPases tended to produce robust structural models with the expected canonical structural features. However, in some of the structural models, conserved sequences were sometimes excluded and replaced with sequences from variable regions to form the secondary elements of the ATPase domains. This suggests that the variable regions may interfere with the ability of the templates to generate accurate and robust models. If true, this may be an important limitation in the analysis of *Plasmodium* proteins, which often contain large regions of low-complexity sequences [14,15].

In addition, detailed analyses of the polyamine-binding site in the modeled structures of ATPase1 and ATPase3 were carried out. The predicted structures using a P5B-subtype as the template are quite similar to the experimentally determined polyamine-binding site and most of the key amino acids are conserved, especially in ATPase1. The differences between ATPase1 and ATPase3 opens the possibility of different substrate specificities. However, as discussed in detail [13], neither ATPase1 nor ATPase3 are likely to be polyamine transporters and neither ATPase1 nor ATPase3 appear to be located in the lysosome, which is called the digestive vacuole in the malaria parasite [16]. Immunofluorescence studies using antibodies against ATPase1 or ATPase3 reveal a diffuse vesicle-like cytoplasmic staining [17,18,19]. As previously discussed [20], this immunofluorescence pattern is consistent with the known ultrastructure of the ER in malaria parasites [21,22]. The possible ER-localization of ATPase1 and ATPase3 indicates that these *Plasmodium* subtype-P5B ATPases may have a different function than the subtype-P5B ATPases of fungi or metazoans.

To address these potential limitations with three-dimensional modeling and to gain insight into the structure and function of type-P5 ATPases from the malaria parasite, Spf1, a rather well-characterized subtype-P5A ATPase [9], and ATP13A2, a rather well-characterized subtype-P5B ATPase [11], were used as templates to investigate the sequence and structural homologies of *Plasmodium* subtype-P5A ATPase (*Pl*P5A), ATPase1, and ATPase3 from *P. falciparum* and *P. relictum*. These two templates were particularly robust in the previous study at predicting structures that recapitulated the experimentally determined structures [13]. The two species not only serve as replicate analyses but also may provide insight into the effects of the variable regions since the *P. relictum* proteins tend to have smaller variable regions.

## 2. Materials and Methods

### 2.1. Identification of Subtype-P5A ATPases from Haemosporidians and the SAR Supergroup

The *P. falciparum* subtype-P5A ATPase (Gene ID: PF3D7_0727800) was used as a query in BLASTP searches [23] of non-redundant protein sequences at NCBI and PlasmoDB [24]. Alignments associated with the BLAST results were used to eliminate partial sequences and sequences with obvious errors. Subsequently, the newly identified sequences were used as queries in BLAST searches to ensure that no subtype-P5A sequences were missed. In cases where complete sequences were available from multiple strains of a single species, the reference strain for that species was chosen. For the haemosporidians, a chromosome number was recorded when available. A partial genome sequence of *Haemoproteus tartakovskyi*, which is not part of the non-redundant database, is available [25]. A TBLASTN search of those contigs was performed using the subtype-P5A ATPase from *P. relictum* as a query. A complete gene sequence was identified on a single contig (HtScaffold0006) and corrected by the insertion of a single A residue. A summary of all the sequences is found in Table S1.

A conserved-domain (CD) analysis was also carried out [26] and the e-values associated with the subtype-P5A (accession number cd07543) and subtype-P5B (accession number TIGR01657) ATPases were recorded.

### 2.2. Sequence Alignments

The haemosporidian subtype-P5A sequences were aligned with ClustalW within Mega XI (Version 11) [27]. Alignments were adjusted manually to accommodate variable regions and core regions of P-type ATPases. The adjusted alignments were used to generate phylogenetic trees (Figure S1).

Subtype-P5A and subtype-P5B ATPases from *P. falciparum* and *P. relictum* were subjected to a detailed analysis of their sequences and predicted 3-dimensional structures, which were compared to Spf1 and ATP13A2 (Table 1). Spf1 was used as a prototype of subtype-P5A ATPases and ATP13A2 was used as a prototype of subtype-P5B ATPases. The *P. falciparum* subtype-P5A ATPase was originally described in a survey of transporter genes as Gene ID PF07_0115 [2], which was later changed to PF3D7_0727800. This sequence was also used in the phylogenetic analysis of type-P5 ATPases [12]. The *P. relictum* orthologue of subtype-P5A was identified in PlasmoDB [24]. ATPase3 is a subtype-P5B ATPase found in apicomplexans and ATPase1 arose from a gene duplication early in the evolution of the malaria parasite and is only found in some malaria species [13].

The initial alignment of these eight ATPases was carried out using ClustalW within Mega XI [27]. This alignment was adjusted manually using 3-dimensional structures generated by Swiss Model [28] as a guide to determine the boundaries between the various domains. The transmembrane helices of the M-domain were determined from the membrane annotation of Swiss Model predictions. The first residue after the β-sheet in the NTD was used as the boundary between the NTD and the A-domain. The first residue of β-strand-1 and the last residue of β-strand-6 of the modified Rossmann fold were used as the boundaries between the P-domain and N-domain. Furthermore, aligning the β-strand-2 from the subtype-P5A and subtype-P5B ATPases greatly improved the alignment. Boxshade (https://junli.netlify.app/apps/boxshade/) (last accessed on 4 September 2025) was used to denote identical (shaded in black) or similar (shaded in gray) residues.

Pairwise distances of the conserved regions of the ATPases were also determined in Mega XI after removing the variable regions of the low-complexity sequence from the alignment as previously recommended [7,29].

### 2.3. Prediction of 3-Dimensional Structures

Homology modeling of the eight type-P5 sequences was carried out with Swiss Model^®^ (https://swissmodel.expasy.org (accessed on 23 August 2025)) [28] using two templates each from Spf1 (PDB ID 6xmq and 6xmu) and ATP13A2 (PDB ID 7m5v and 7m5x). Templates 6xmq and 7m5v were bound with a non-hydrolysable ATP analog (AMP-PCP) and templates 6xmu and 7m5x were bound with BeF_3_ and their respective substrates of either a transmembrane helix or spermine. These templates were previously identified as reliable templates in Phyre^®^ analyses using the ATPase1 and ATPase3 sequences to search structural databases [13]. Homology modeling was also carried out on the *Plasmodium* ATPases after removal of large regions of low complexity and variable sequence.

Four criteria were used to assess the quality of the predictions by Swiss Model: the Global Model Quality Estimation (GMQE), the QMEANDisCo, the percentage of Ramachandran favorable ψ and Θ angles, and the retention of ligands. GMQE predicts the expected accuracy of a protein model. QMEANDisCo is a composite scoring function that provides both global and local quality estimates for a protein model. Non-covalently bound ligands are retained in models if there are at least three coordinating residues in the protein and those residues are conserved in the target–template alignment, and if the resulting atomic interactions in the model are within the expected range for van der Waals interactions and water-mediated contacts.

Three-dimensional structures were also generated with AlphaFold-3 [30]. AlphaFold uses artificial intelligence and machine learning to predict a protein’s 3D structure based on its primary amino acid sequence by aligning the sequence to similar proteins and identifying regions that tend to change together in evolutionary time. The structures of Spf1 and ATP13A2 generated with AlphaFold were compared to the structures produced by Swiss Model with concordant templates since the disorganized regions were often excluded from the experimentally determined structures. The ranking score, pTM, and fraction of the disorganized sequence generated by AlphaFold were recorded. A pTM score above 0.5 means the overall predicted structure may be similar to the true structure. Ranking scores higher than 0.8 represent high-quality predictions with a high level of confidence. Values below 0.6 are likely failed predictions and values between 0.6 and 0.8 may be either correct or incorrect.

Images were generated with PyMOL 3.1.3 (Schrodinger, LLC, New York, NY, USA). The various domains were demarcated in different colors, and the color names refer to the names given by PyMOL. Color scheme: nMH or nML (limon), cTM (yelloworange), sTM (light orange), NTD (aquamarine), A-domain (tv_green), N-domain (skyblue), P-domain (violetpurple), cTM4 kink and signature motif (magenta), phosphorylated asp (yellow), P-domain arm (purpleblue), and IDL (salmon).

## 3. Results

### 3.1. Subtype-P5A ATPase of Haemosporida

The *P. falciparum* subtype-P5A ATPase (*Pf*P5A) was originally described in a survey of transporter genes as a presumptive cation transporter [2] and subsequently included in a phylogenetic analysis of type-P5 ATPases [12]. Searches of PlasmoDB and NCBI identified orthologues of subtype-P5A ATPase from other haemosporidians (Table S1). All the haemosporidian genes have a single exon and their chromosomal locations correspond to the known gene synteny of *Plasmodium* [31]. A search of conserved domains [26] identified cd07543 (subtype-P5A) and TIGR01657 (subtype-P5B) as the two highest scoring domains based on E-values. In both cases, the E-values were extremely low, and as expected the cd07543 tended to be lower than TIGR01657. This confirms that the sequences are subtype-P5A ATPases but also indicates that subtype-P5A and subtype-P5B are very similar.

Alignment of the haemosporidian subtype-P5A orthologues revealed regions of very high sequence homology interspersed with variable regions of low sequence homology (Figure S1a). These haemosporidian subtype-P5A ATPases exhibit a similar phylogeny as reported for ATPase3 [13] and separate into eight clades (Figure S1b,c) that are consistent with the current views on the phylogeny of malaria parasites [32,33]. The eight clades are *Haemoproteus*, avian parasites, *Laverania*, rodent parasites, *Hepatocystis*, malariae, ovale, and vivax-like. As previously observed with ATPase3 [13], *Haemoproteus*, avian parasites, and *Laverania* form a clade and a second clade is formed from the other mammalian parasites including *Hepatocystis* (Figure S1b).

The *Plasmodium* subtype-P5A ATPases (*Pl*P5A) have N-terminal extensions (NTE) not found in either Spf1 or ATP13A2. ATPase3 also has an NTE but ATPase1 does not [13]. The NTE of *Pl*P5A is longer than the NTE of ATPase3 and there is no shared sequence homology (Figure S2). In the case of ATPase3, there is substantial sequence homology of the NTE within the major apicomplexan clades (i.e., haemosporidians, piroplasmids, and coccidians), whereas there is little homology between these clades [13]. The first half of the NTE of subtype-P5A exhibits a high degree of sequence homology across all haemosporidians and the second half of the NTE exhibits little sequence homology (Figure S1). This creates an insertion of a variable region between the NTE and the NTD designated as variable region 1 (VR1). Six additional variable regions (VR2-VR7) are found in the haemosporidian orthologues of subtype-P5A ATPase. These inserts tend to be low-complexity sequences with a high preponderance of asparagine and lysine residues, and tandem repeats are sometimes observed (Figure S1a).

### 3.2. Sequence Homology Between Subtype-P5A and Subtype-P5B ATPases of Malaria Parasites

ATPase1, ATPase3, and *Pl*P5A from *Plasmodium falciparum* and *P. relictum* were aligned with Spf1 (subtype-P5A) and ATP13A2 (subtype-P5B). The alignment exhibited regions of high sequence homology between all eight proteins, interspersed with regions of little sequence homology (Figure S2). The region including the NTD and the N-terminal membrane loops (nML) exhibit a moderate level of homology. The regions of highest sequence homology correspond to the canonical domains of P-type ATPases (A, N, P, and M). Other than the P-domain (discussed below), there were no substantial regions in which the sequence homology clearly segregated into either subtype-P5A or subtype-P5B. This is consistent with the paucity of specific signature sequences to distinguish subtype-P5A and subtype-P5B [8,12].

Large stretches of low-complexity sequence are inserted between or within the highly conserved canonical domains of the *Plasmodium* type-P5 ATPases. This was previously described in ATPase1 and ATPase3 as four large variable regions inserted into the NTD, A-domain, N-domain, and P-domain, referred to as variable regions (VR) 1–4, respectively [13]. Subtype-P5A ATPase of *Plasmodium* has seven substantial inserts of low-complexity sequence that exhibit sequence variation (Figure S1a). The locations of some of the inserts are exclusive to either subtype-P5A or subtype-P5B, whereas three inserts have a shared location between the two subtypes (Table 2). Inserts with shared locations tend to be substantially larger in one of the two subtypes. In general, the inserts of the subtype-P5A ATPases tend to be smaller in size than the subtype-P5B ATPases. However, the total percentage of the ATPases that are associated with variable regions is approximately the same between subtype-P5A and subtype-P5B. If present, the insert sizes within Spf1 and ATP13A2 are substantially smaller.

An alignment with the low-homology inserts removed was used to generate a pairwise distance matrix of the eight type-P5 ATPases (Table 3). Comparisons of ATPases of the same subtype were designated as concordant and comparisons between subtype-P5A and subtype-P5B ATPases were designated as discordant. The distance between Spf1 (subtype P5A) and ATP12A2 (subtype P5B) is 1.39 and represents the divergence subtype-P5A and subtype-P5B from early in eukaryote evolution. The distances between other discordant sequences exhibit similar values (1.36–1.59). The concordant subtype-P5B sequences also exhibit similar values (1.24–1.56), whereas the values from concordant subtype-P5A sequences were a little lower (1.14–1.16). Even though ATPase1 and ATPase3 are subtype-P5B ATPases, they are also essentially equal distance from subtype-P5A (1.41–1.59) and subtype-P5B (1.24–1.56) templates. As expected, the *P. falciparum* and *P. relictum* orthologues are the most closely related (0.13–0.42) and reflect a substantially more recent divergence on the order of 10 million years ago [34].

### 3.3. Homology Modeling and Structure Comparisons

The experimentally determined structures of Spf1 and ATP13A2 were used as templates to carry out homology modeling of the *Plasmodium* type-P5 ATPases. As controls, the Spf1 and ATP13A2 sequences were modeled with the same template (i.e., concordant) or the other template (i.e., discordant). Modeling with either the concordant or discordant templates produced structures that were similar to each other and to the experimentally determined structures. This is especially true for the predicted structures of the M-, A-, N-, and P-domains. Neither template provided much insight into the structures of the N-terminal and C-terminal extensions, since these sequences were usually excluded from the models.

**Figure 2:**
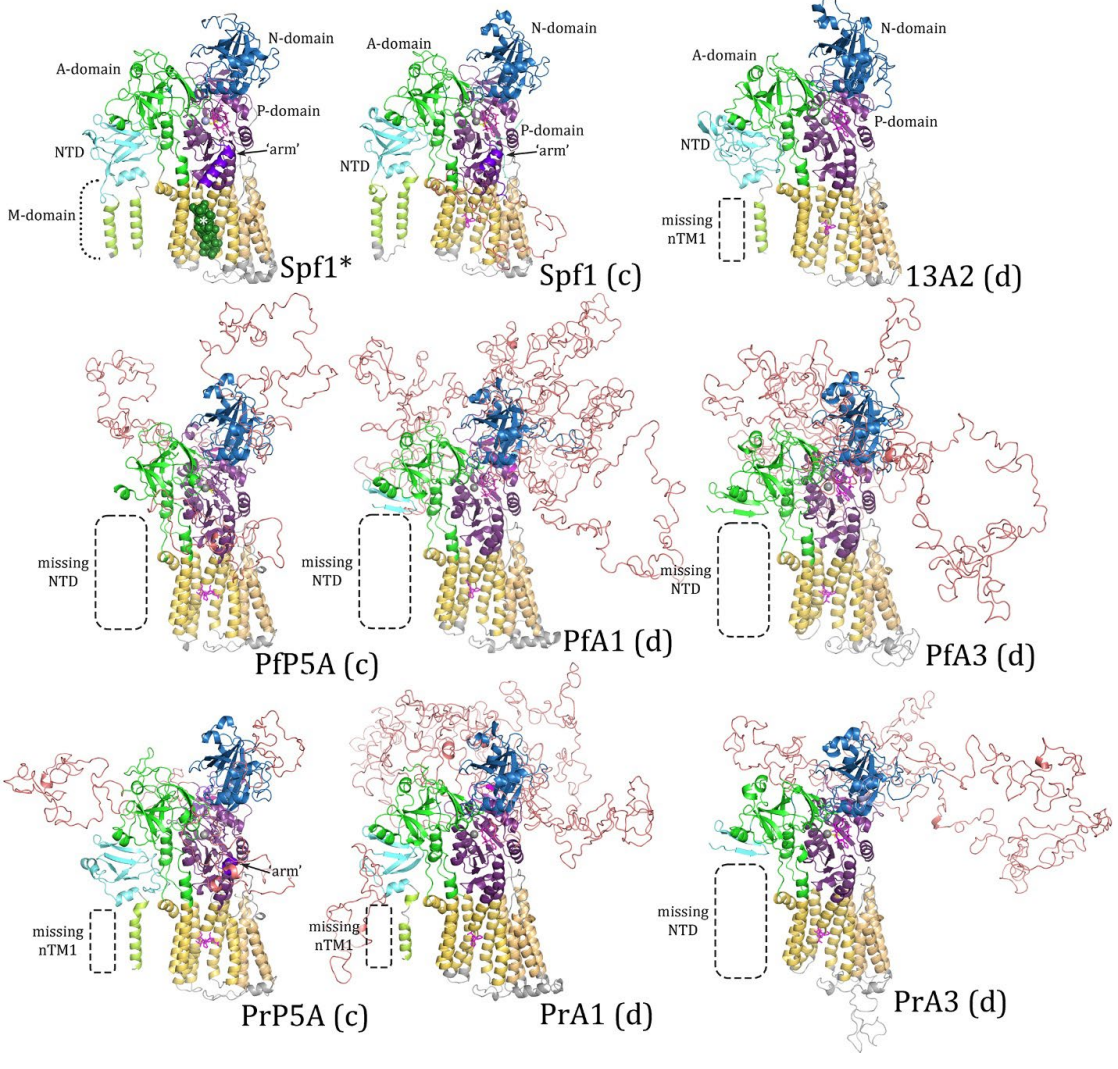
Homology modeling with the Spf1 template. *Plasmodium* subtype-P5A, ATPase1 (A1), and ATPase3 (A3) sequences from *P. falciparum* (Pf) and *P. relictum* (Pr) were modeled by Swiss Model^®^ with the Spf1 (PDB Acc. No. 6xmu) template. Spf1 * is an experimentally determined structure and concordant (c) and discordant (d) models are noted. Dashed boxes denote missing elements in the modeled structures. The ‘arm’ (arrow, dark purple) is an α-helix that is specific to subtype-P5A. The A-domain is colored green, the N-domain blue, the P-domain purple, the transmembrane helices yellow, and the NTD aqua, with the membrane-associated loops being colored light green. The intrinsically disordered loops (IDLs) produced by the variable regions are salmon. The white asterisk (*) denotes the α-helix substrate (dark green) in the substrate-binding groove of Spf1 *.

**Figure 3:**
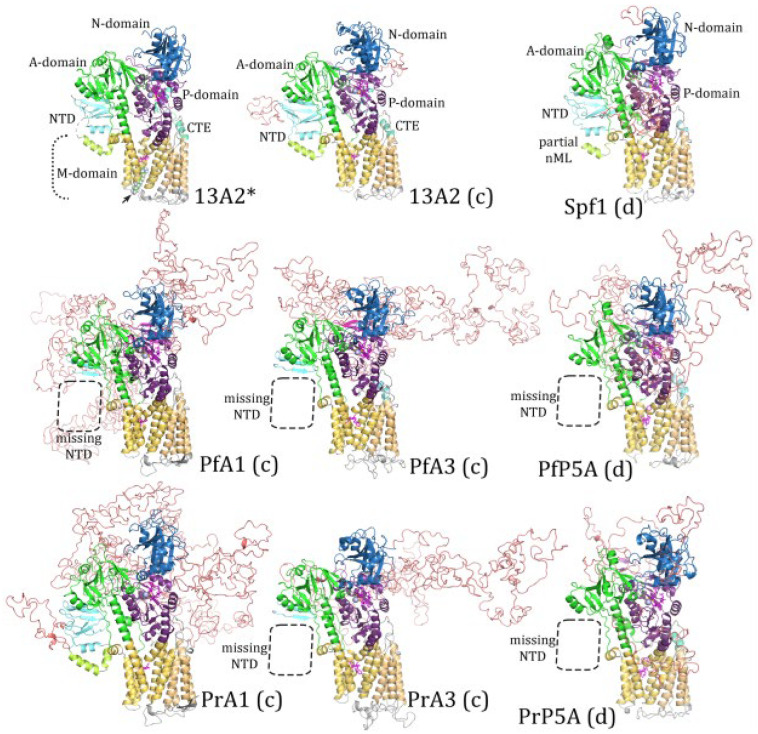
Homology modeling with the ATP13A2 template. *Plasmodium* subtype-P5A, ATPase1 (A1), and ATPase3 (A3) sequences from *P. falciparum* (Pf) and *P. relictum* (Pr) were modeled by Swiss Model^®^ with the ATP13A2 (PDB Acc. No. 7m5x) template. 13A2 * is an experimentally determined structure and concordant (c) and discordant (d) models are noted. Dashed boxes denote missing elements in the modeled structures. The A-domain is colored green, the N-domain blue, the P-domain purple, the transmembrane helices yellow, and the NTD aqua, with the membrane-associated loops being colored light green. IDLs produced by the variable regions are colored salmon. The arrow denotes the spermine substrate in the substrate-binding groove of 13A2.

As expected, differences between subtype-P5A and subtype-P5B were primarily in the NTD, including the N-terminal membrane loops and the arm in the P-domain. Concordant templates produced more complete structures than discordant templates. For example, modeling Spf1 with the Spf1 template generated two N-terminal transmembrane helices as is found in subtype-P5A (Figure 2), and the discordant template generated a single N-terminal transmembrane helix (Figure 3). Similarly, modeling ATP13A2 with the concordant template generated the expected triangular membrane loop that is found in subtype-P5B ATPases (Figure 3) and the triangular loop generated by the discordant template was somewhat incomplete (Figure 2). Another feature specific to subtype-P5A ATPases is a α-helical ‘arm’ that projects from the P-domain. The arm is evident in the experimentally determined structure, and a shorter version is predicted in the concordant model while no arm is generated with the discordant template.

In general, the predicted structures of the *Plasmodium* ATPases are similar to the templates, and the major domains of P-type ATPases are readily discernable. However, neither template did an extremely good job at generating the NTD including the associated membrane loops. No NTD was generated with the Spf1 template in four of the *Plasmodium* ATPases, and the first transmembrane helix was lacking in the other two ATPases (Figure 2). Similarly, the NTD was lacking in five of the *Plasmodium* ATPase models generated with the ATP13A2 template (Figure 3). However, in PrATPase1 a complete triangular membrane loop was generated.

### 3.4. Quality Assessment of Modeling

The quality of the structural models produced from concordant and discordant templates was assessed with two templates representing different conformational states of both Spf1 and ATP13A2 using four criteria, as described in the Methods (Table 4). Models of Spf1 or ATP13A2 produced with concordant templates represent the maximum possible quality scores and serve as positive controls, whereas scores from discordant sequence and template pairs represent the magnitude of the difference between subtype-P5A and subtype-P5B. The *Pl*P5A sequences modeled with the Spf1 templates (i.e., concordant) had notably higher GMQE scores and slightly higher QMEANDisCo scores than those modeled with the discordant templates (7m5x and 7m5v). This was not observed with the *Plasmodium* subtype-P5B ATPases, which exhibited essentially the same quality scores with either the concordant or discordant templates. The *P. falciparum* sequences have lower GMQE scores than the *P. relictum* sequences, which is likely due to the smaller variable regions in the *P. relictum* proteins. Magnesium was the most frequently retained ligand, and was only excluded in a few instances and only with templates bound with non-hydrolysable ATP analogs. The substrates and non-hydrolysable ATP analogs were not retained in any models. There were a few instances of models retaining the BeF_3_ using the ATP13A2 template.

The quality assessments indicate that the models of the *Plasmodium* type-P5 ATPases are relatively accurate, and the quality assessments did not substantially favor one template over the other in subtype-P5B ATPases. In contrast, the concordant template (i.e., Spf1) was favored in the subtype-P5A ATPases.

### 3.5. A-Domain

**Figure 4:**
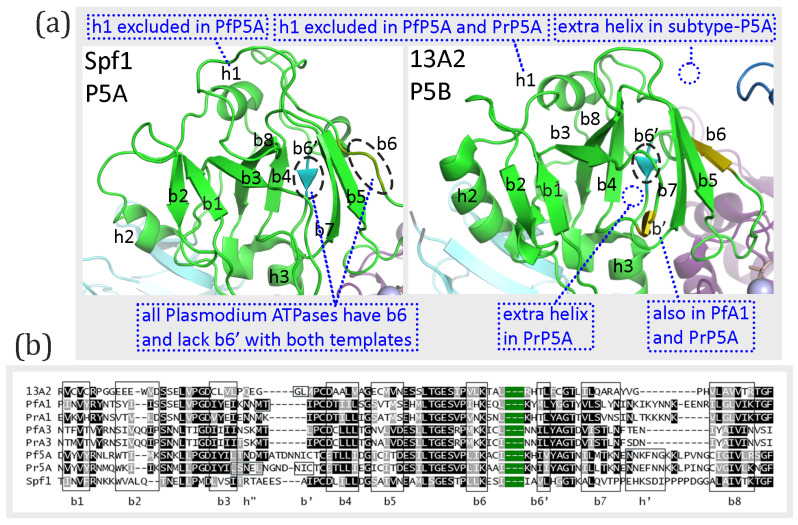
A-domain of type-P5 ATPases. (**a**) Shown are the experimentally determined structures of the A-domain, with the eight β-strands (b1–8) of the distorted jelly roll and the three associated a-helices (h1–3) denoted. Beta-strand-6 in ATP13A2 is colored differently, as well as the corresponding segment in Spf1 (circled). The circled short β-strand (b6′) in teal was found in experimentally determined structures but none of the modeled structures (Figure S3). The blue annotations refer to differences in the Plasmodium type-P5 ATPases modeled with either template. (**b**) The sequence alignment corresponding to the distorted jelly roll with the β-strands boxed. Additional β-strands (b′) and helices (h′ and h″) are denoted. The green segment denotes a region of intrinsically disordered sequence that corresponds to variable region 2 of ATPase1/3 or variable region 4 of PlP5A. Black or gray shading indicates identical or similar residues, respectively.

The A-domain is formed by a segment between the NTD and cTM1 plus the loop between cTM2 and cTM3. The loop between cTM2 and cTM3 is composed of eight primarily anti-parallel β-strands, which form a structure called a distorted jelly roll [6]. The order of the β-strands in the β-sheet according to linear amino acid sequence is b2-b1-b3-b8-b4-b7-b5-b6, and b4 and b7 are parallel while the other β-strands are anti-parallel. Two α-helices connected to cTM1 and a third α-helix connected to cTM3 are stacked against this β-sheet. These basic features of the distorted jelly roll are found in the experimentally determined structures of Spf1 and ATP13A2, except for some minor differences (Figure 4a). For example, Spf1 appears to be missing β-strand-6 and both Spf1 and ATP13A2 have a short β-strand of two amino acids (designated b6′) that is found between β-strand-4 and β-strand-7, which is not found in the standard distorted jelly roll. This short β-strand (b6′) is not observed in any of the models generated with either concordant or discordant templates, and β-strand-6 is present in all the models generated with both templates (Figure S3). Except for the perplexing case of the experimentally determined structure of Spf1, the distorted jelly roll is the same as the known structure of the A-domain of type-P5 ATPases.

The predicted A-domain structures of the *Plasmodium* type-P5 ATPases are similar to the experimentally determined structures, and the same structures were produced with either concordant or discordant templates (Figure S3). Differences in the modeled *Plasmodium* proteins were minor, inconsistent, or of dubious importance. All three subtype-P5A ATPases modeled with the ATP13A2 template (discordant) had an extra helix in a short segment of low sequence homology between β-strand-7 and β-strand-8 (Figure 4b). However, this helix (h′) was not observed in the experimentally determined structure of Spf1 nor in the predicted structures with the concordant template (6xmu). An additional short β-strand (b′) that is not part of the β-sheet was observed in PfATPase1 and PrP5A and an additional short helix (h″) is seen in PrP5A. This additional β-strand is also seen in the experimentally determined ATP13A2 structure but not found in the modeled structures produced with either template. These additional elements are found in regions of low sequence homology (Figure 4b). A large portion of N-terminal sequence, including part of the A-domain, was excluded from the *P. falciparum* subtype-P5A structure modeled with either 6xmu or 7m5x templates and from the *P. relictum* subtype-P5A structure modeled with 7m5x. In summary, there are no major differences in the A-domain between subtype-P5A and subtype-P5B ATPases, including the *Plasmodium* type-P5 ATPases.

### 3.6. N-Domain

**Figure 5:**
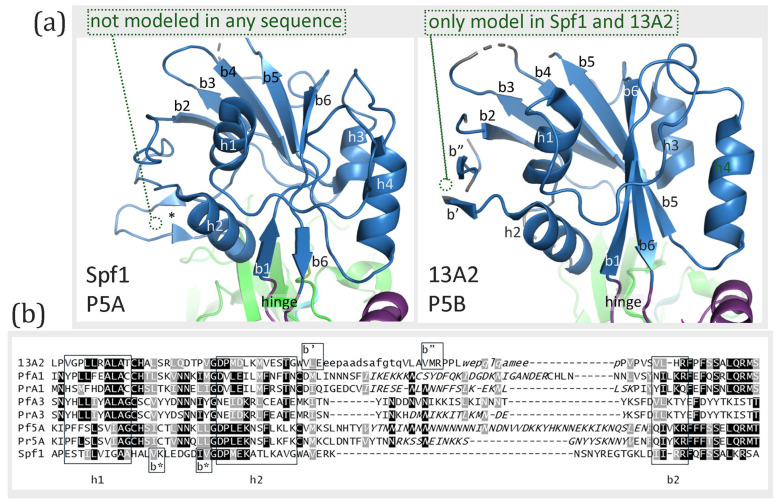
N-domain of Spf1 and ATP13A2. (**a**) Shown are the experimentally determined structures of the N-domain with the six β-strands (b1–6) and four associated a-helices (h1–4) denoted. Two extra short β-strands (b *) are observed in Spf1, which are not modeled in any of the other ATPases (Figure S4). Similarly, two extra β-strands (b′ and b″) are observed in ATP13A2, which are not modeled in the *Plasmodium* ATPases. (**b**) The sequence alignment corresponding to the region of the extra β-strands in relation to helix-1, helix-2, and β-strand-2 (boxed). Black or gray shading indicates identical or similar residues, respectively.

N-domains have been described as either a six-stranded twisted antiparallel β-sheet [35] or a seven-stranded twisted antiparallel β-sheet [6,36] flanked by four [6,35] or five [36] a-helices. The basic structure of the N-domain of both Spf1 and ATP13A2 consists of a twisted six-stranded anti-parallel β-sheet flanked by four a-helices (Figure 5); thus, both are similar to the Cu-transporting P-type ATPase [35]. Additional β-strands are observed in both Spf1 and ATP13A2 (Figure 5). Two additional β-strands of two amino acids each are observed in Spf1 between α-helix-1 and α-helix-2 and are not likely to be incorporated into the twisted β-sheet. These short β-strands are not found in any of the modeled structures (Figure S4a). In ATP13A2 the additional β-strands are located between α-helix-2 and β-strand-2 and are potentially in close enough proximity to be part of the β-sheet. These two β-strands are found in the model structures of ATP13A2 and Spf1 using the ATP13A2 template but are not found in any of the modeled *Plasmodium* ATPases (Figure S4b). The sequence encoding these additional β-strands is in a short non-conserved region that tends to form IDL (Figure 5b), thus raising questions about their validity or significance. Except for the extra β-strands, the N-domain structures of Spf1 and ATP13A2 are nearly identical.

The predicted structures of the N-domains from the *Plasmodium* type-5 ATPases are very similar to the structures of Spf1 and ATP13A2 except for the lack of the extra β-strands (Figure S5). No substantial differences between subtype-P5A and subtype-P5B ATPases are seen in the N-domain and no unique features of the *Plasmodium* type-5 ATPases were observed. The N-domains of all type-P5 ATPases are highly conserved in regard to its secondary structural features.

### 3.7. P-Domain

**Figure 6:**
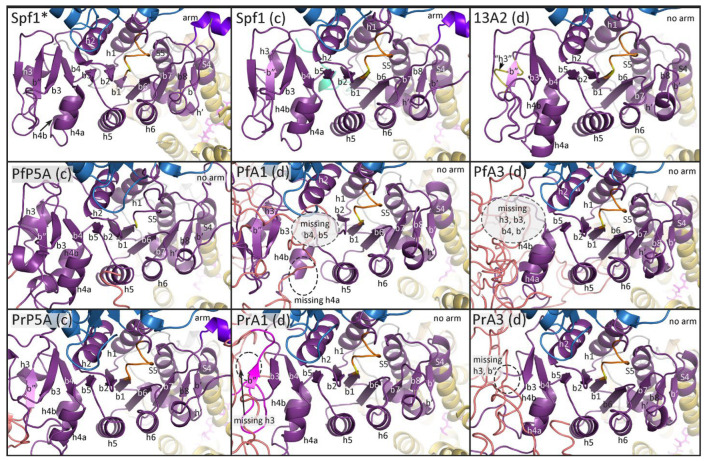
Homology modeling of the P-domain with the Spf1 template. Three-dimensional structures of the P-domain from Spf1, ATP13A2, and the six *Plasmodium* type-P5 ATPases were generated with the Spf1 template (6xmu) and compared to the experimentally determined structure of Spf1 (*). Subtype-P5A sequences are designated as concordant (c) and subtype-P5B are designated as discordant (d). Eight parallel β-strands (b1–8) form a wavy β-sheet that is flanked by two anti-parallel β-strands (b′ and b″). The a-helices include two helices that continue from cTM4 (S4) and cTM5 (S5) and form stalks (S), six helices of the modified Rossmann fold (h1–6), and an additional helix (h′). Missing secondary structural elements are denoted with dashed circles and secondary elements derived from variable regions are magenta. The “h3” (arrow) denotes a region of random coil that has a helical shape (yellow) in ATP13A2.

**Figure 7:**
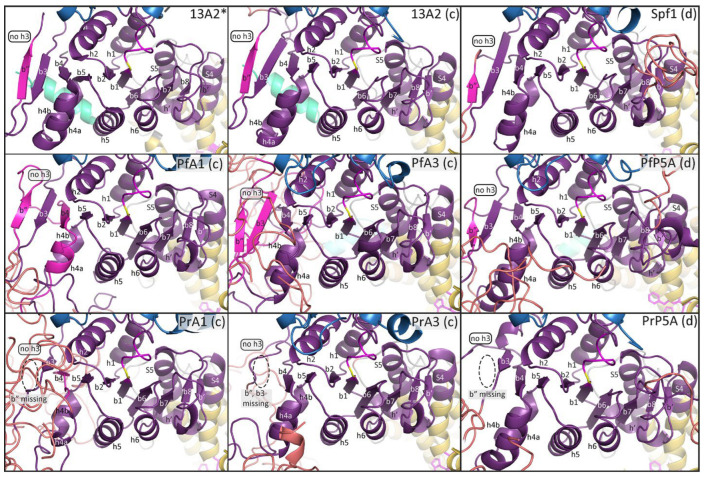
Homology modeling of the P-domain with the ATP13A2 template. Three-dimensional structures of the P-domain from Spf1, ATP13A2, and the six *Plasmodium* type-P5 ATPases were generated with the ATP13A2 template (7m5x) and compared to the experimentally determined structure of ATP13A2 (*). Subtype-P5B sequences are designated as concordant (c) and subtype-P5A are designated as discordant (d). Eight parallel β-strands (b1–8) form a wavy β-sheet that is flanked by two anti-parallel β-strands (b′ and b″). The a-helices include two helices that continue from cTM4 (S4) and cTM5 (S5) and form stalks (S), five helices of the modified Rossmann fold (h1–2, h4–6), and an additional helix (h′). Missing secondary structural elements are denoted with dashed circles and secondary elements derived from variable regions are magenta. The no-h3 box highlights that α-helix-3 is not found in ATP13A2, nor in any of the models generated with ATP13A2 as the template.

The P-domain of SERCA is described as a modified Rossmann fold with a six-stranded parallel β-sheet and six associated a-helices [6]. The Rossmann fold is a common tertiary structure found in proteins that bind nucleotides [37]. This modified Rossmann fold is formed from alternating β-strands and a-helices, with the resulting β-sheet being somewhat sandwiched between two rows of a-helices. The fourth helix has a kinkwhich results in two closely associated helices that are designated as helix-4a and helix-4b. For the most part, the β-strands and a-helices making up this modified Rossmann fold are readily identified in the experimentally determined structure of Spf1 and ATP13A2 (Figure 6 and Figure 7). In addition, two additional parallel β-strands (b7 and b8), two additional anti-parallel β-strands (b′ and b″), and an additional α-helix (h′) are observed.

The experimentally determined structures of Spf1 and ATP13A2 are quite similar to each other except that ATP13A2 is missing the α-helix-3 (Figure 7). However, a homology modeling of the ATP13A2 sequence with the Spf1 template results in random-coil sequence in the shape of a helix at this position (Figure 6), and modeling of the Spf1 sequence with the ATP13A2 template results in the loss of this third helix (Figure 7). In other words, the structures generated with concordant templates better recapitulated the actual experimentally determined structures. These results imply that there may be a difference between the Rossmann folds of subtype-P5A and subtype-P5B ATPases, especially in the region of the third helix.

Another unique feature of the subtype-P5A P-domain is the presence of an a-helical projection called the arm. Following the arm is an intrinsically disordered region that may interact with the membrane [9]. The arm is readily identified in the experimentally determined structure of Spf1 and in predicted structure of Spf1 using Spf1 as a template (Figure 6). As expected, no arm is seen in either the experimentally determined structure of ATP13A2 or the predicted structure using the Spf1 template. These results confirm that subtype-P5A and subtype-P5B differ in regard to the presence of the arm structure between the P-domain and cTM5.

**Figure 8:**
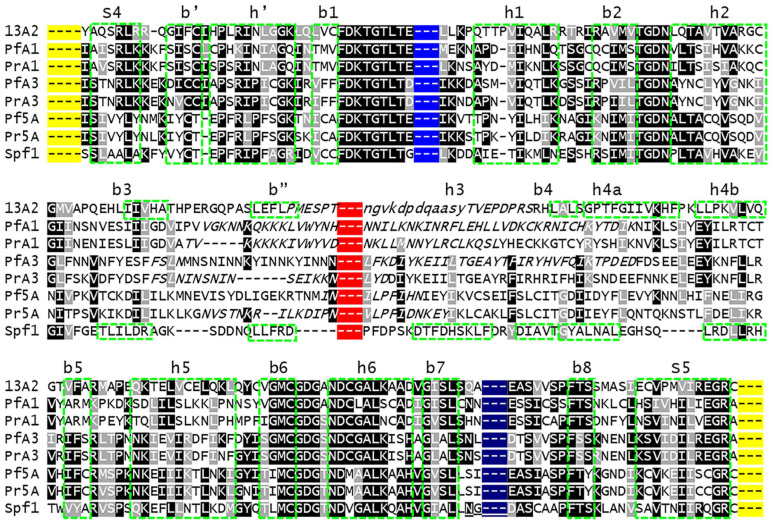
P-domain alignment. The secondary structural elements of the P-domain (Figure 6 and Figure 7) are boxed (green dashes) and labeled as β-strands (b #), a-helices (h #), or stalks (s #). In the low-homology region between h2 and h4b, only the experimentally determined secondary structures are boxed. The yellow shading represents cTM4 and cTM5, which flank the P-domain; the blue shading represents the N-domain; the red shading represents VR6 in Pl5A or VR4 in ATPase1/3; and the dark blue shading between b7 and b8 represents the arm and associated IDL (VR7) of the subtype-P5A ATPases. Sequences in lower case are excluded from the experimentally determined three-dimensional structures and italicized sequence are from IDL.

Homology modeling of the *Plasmodium* subtype-P5A ATPases with the Spf1 (i.e., concordant) generates structures similar to the experimentally determined Spf1, with all the expected secondary structures of the Rossmann fold (Figure 6). However, a shorter-than-expected arm is predicted in the PrP5A structure, and no arm is predicted in the PfP5A structure. As expected, no arms are predicted in ATPase1 or ATPase3. In contrast, there were several missing secondary structural elements in ATPase1 and ATPase3 (i.e., subtype-P5B *Plasmodium* sequences) modeled with Spf1 (i.e., discordant), as well as β-strand-b″ of *Pr*ATPase1 being generated from the variable region-4 sequence. The ATP13A2 template performed approximately equally well with *Plasmodium* types-P5A (i.e., discordant) and subtype-P5B ATPases (i.e., concordant) (Figure 7). For example, the ATP13A2 template also generated several instances of a variable sequence being incorporated into the secondary structures of the P-domain in both concordant and discordant structures. There were also several instances of missing secondary structural elements in both concordant and discordant structures. These anomalies are restricted to the central part of the P-domain in a region that does not exhibit a high level of sequence homology and that includes VR4 of ATPase1/3 and VR6 of PlP5A (Figure 8). In particular, β-strand-3, helix-3, β-strand-4, and helix-4a are in a region of low sequence homology.

### 3.8. Variable Regions Effects

Overall, the predicted structures of the *Plasmodium* type-P5 ATPases are quite similar to the experimentally determined structures, and this is especially true for the A- and N-domains. The discrepancies in the P-domain of the predicted structures are often associated with the variable regions composed of IDL. The potential effects of the variable regions were analyzed by modeling the *Plasmodium* subtype-P5B ATPase sequences with or without variable regions using the Spf1 and ATP13A2 templates. In general, removing the variable regions more than doubled the GMQE scores, increased the QMEANDisCo scores by 20% or more, and increased the percentage of Ramachandran favorable ψ and Θ angles by 10% or more (Table 4). The retention of ligands did not appear to be affected by the presence or absence of variable regions.

Removing the variable regions for the most part either had no effect on the predicted structures or improved the homology modeling by generating structures that better recapitulated the experimentally determined structures (Figure S5 and Table 5). For example, most of the predicted structures of ATPase1 and ATPase3 were missing part of the NTD and removing the variable regions partially or completely restored the membrane associated helices of the NTD. Similarly, for the most part, the minor discrepancies in the P-domain were corrected by removing the variable regions. When there were no discrepancies, removing the variable regions did not introduce discrepancies except for a loss of β-strands in the P-domain of PrATPase3. There was no obvious correlation between the size of the variable regions and whether their removal had a positive, neutral, or negative impact on the predicted three-dimensional structures.

### 3.9. Substrate-Binding Site

**Figure 9:**
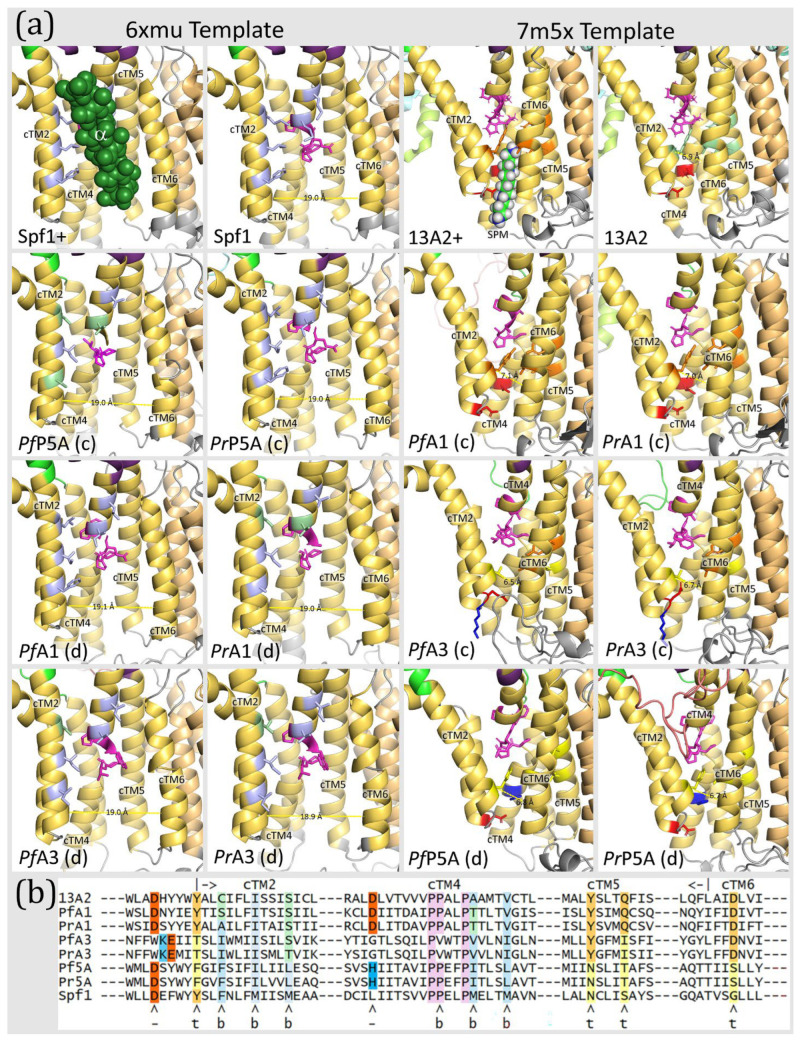
Substrate-binding sites of *Plasmodium* type-P5 ATPases. (**a**) Predicted structures of the substrate binding sites of the *Plasmodium* type-P5 ATPases modeled with the Spf1 (6xmu) or ATP13A2 (7m5x) templates. The top row shows the experimentally determined structures of Spf1 or ATP13A2, with (+) or without their respective alpha-helix (α) or spermine (SPM) substrates. Other rows show ATPase1 (A1), ATPase3 (A3), or subtype-P5A from *P. falciparum* (Pf) and *P. relictum* (Pr) modeled with concordant (c) or discordant (d) templates. The dashed yellow line is the distance between cTM2 and cTM6 measured in angstroms (Å), demonstrating the width of the substrate groove. Sidechains of the amino acids making up the kink in cTM4 are shown and colored magenta. Sidechains of amino acids that may interact with the a-helical substrate of subtype-P5A are shown and colored light blue for nonpolar residues and light green for polar residues, except for the proline in the kink. Sidechains making up the tetrad that stabilizes the substrate groove of subtype-P5B are shown and colored orange if conserved with ATP13A2 or yellow if not conserved. The sidechains of the two aspartate residues that may interact with the polyamine substrate are shown and colored red. Nearby negatively charged sidechains are also colored red and nearby positively charged residues are colored blue. (**b**) Alignment of sequences making up the substrate-binding groove. Carets (^) below the alignment denote residues that bind to substrate (b) in subtype-P5A, make up the stabilizing tetrad (t) in subtype-P5B, or provide negative charges (-) for polyamine binding in subtype-P5B. Shading corresponds to the colors used in the homology models. The |-> <-| denotes the residues used to determine the width of the substrate-binding groove. Three dashes (---) denote a removed sequence.

The substrate-binding sites of P-type ATPases are formed from the core transmembrane helices, and cTM2, cTM4, and cTM6 often interact with the substrate [1]. These helices wrap around to form an incomplete cylindrical structure that makes up the substrate-binding groove. This groove typically opens to the extra-cytoplasmic face of the membrane and the kink in cTM4 forms the base. Subtype-P5A ATPase has a much wider substrate groove than subtype-P5B to accommodate the transmembrane-helix substrate, and the groove opens laterally within the lipid bilayer. This wider substrate groove is due to cTM5 and cTM6 rotating away from the other cTM [8]. For example, the distance between cTM2 and cTM6 is approximately 19 Å in Spf1 and 7 Å in ATP13A2 (Figure 9). Important residues in Spf1 are six non-polar amino acids on cTM2 and cTM4 that interact with the a-helical substrate [12]. These are F223, M227 and M231 of cTM2, P445 of the kink in cTM4, and M449 and M453 of cTM4b. The substrate-binding groove of ATP13A2 is stabilized by interactions between Y259 in cTM2, Y940 and Q944 in cTM5, and D967 in cTM6, called the tetrad [11]. In addition, aspartates that provide negative charges to interact with the positive charges of polyamines were also identified.

Homology modeling of the *Plasmodium* subtype-P5A ATPases with the Spf1 template yields structures that are essentially identical to the experimentally determined Spf1 structures and the six residues implicated in substrate binding are all non-polar (Figure 9). Somewhat unexpectedly, modeling *Plasmodium* subtype-P5A ATPases with the discordant template produces a substrate-binding groove that is essentially identical to the experimentally determined ATP13A2 structure (i.e., subtype-P5B). However, none of the stabilizing tetrad amino acids are conserved, and only one of the aspartates is conserved and the other is replaced with a positively charged histidine. Thus, even though the discordant structure recapitulates the subtype-P5B ATPase, key residues are missing, and therefore the *Plasmodium* subtype-P5A ATPases are not likely to be polyamine transporters.

Likewise, modeling ATPase1 and ATPase3 with the concordant template produced models essentially identical to ATP13A2 and the discordant template produced models essentially identical to Spf1. As previously noted [13], the residues making up the tetrad and the aspartate residues of ATPase1 are identical to ATP13A2, whereas these residues are only partially conserved in ATPase3. Regarding the discordant template, the six non-polar residues implicated in substrate binding are all non-polar in ATPase3, whereas 5/6 of the residues are non-polar in PrATPase1 and 4/6 residues are non-polar in PfATPase1. However, these three polar residues are next to non-polar residues. The conservation of the non-polar residues that interact with the substrate suggests that *Plasmodium* subtype-P5B ATPases may be able to conform to α-helix dislocase activity.

### 3.10. AlphaFold Modeling

The eight type-P5 ATPase sequences were also analyzed by AlphaFold3 to examine the discrepancies with structures produced by homology modeling and specifically focus on the IDL, the N-terminal membrane loops, the arm of subtype-P5A, the substrate-binding site, and the P-domain. Overall AlphaFold produced similar structures as the Swiss Model structures generated with concordant templates (Table 6, Figure S6). One notable difference is in the IDL, where AlphaFold tends to produce a-helices in the IDL (Figure S6a,b). This tendency to invent plausible secondary structures in unstructured regions has been noted as a limitation of AlphaFold and is referred to as hallucination [30]. Furthermore, some of these hallucinated a-helices transcend the M-domain or are found on the extracytoplasmic side of the membrane.

Between the P-domain and cTM5 is an α-helical extension called the arm, followed by disorganized structure that may interact with the cytoplasmic face of the membrane [9]. AlphaFold3 produces an arm structure that is very similar to the experimentally determined Spf1 structure, and this structure appears identical to the previously published AlphaFold2 structure of Spf1 [8]. Homology modeling of Spf1 with the Spf1 template produced a shorter helix than the experimentally determined or AlphaFold structures (Figure S6a). A shorter helix is also predicted for PfP5A by AlphaFold and no helix is predicted for PrP5A. However, a quasi-helix is found in this region of PrP5A. The disorganized sequence following the arm contains substantial a-helical structures in the AlphaFold predicted models which are likely hallucinations. This region corresponds to VR7 and is likely random coil as predicted by Swiss Model.

Swiss Model also had difficulty predicting the N-terminal membrane loops in the *Plasmodium* ATPases (Figure 2 and Figure 3). The N-terminal membrane loop of subtype-P5A has two transmembrane helices and AlphaFold recapitulates this structure in Spf1 (Figure S6c), as did Swiss Model (Figure 2); this structure closely resembles the experimentally determined structure. In contrast, Swiss Model was unable to generate the NTD in PfATPase5A and could only generate one of the N-terminal transmembrane helices in PrATPase5A (Figure 2). AlphaFold generated two N-terminal transmembrane helices in the *Plasmodium* sequences (Figure S6c). However, the IDL produced from VR1 was between the two transmembrane helices, resulting in the IDL being on the extracytoplasmic side of the membrane. This is unprecedented in P-type ATPases [1], and thus is not correct. There are quasi-helical regions and short helices in VR1, which might be able to serve as transmembrane helices that result in the IDL on the cytoplasmic side of the membrane.

The N-terminal membrane of loop of subtype-5B has three short helices that form a triangle [1,8] and both Swiss Model and AlphaFold generate this structure in ATP13A2 (Figure S6b,d). AlphaFold also generated this triangular membrane loop in ATPase1 from both *P. falciparum* and *P. relictum*. However, AlphaFold generated two transmembrane helices in the region corresponding to the N-terminal loops in ATPase3 from both parasites, as well as a hallucinated helix that appears to traverse the membrane. In summary, AlphaFold only predicted the expected structures of the N-terminal membranes for ATPase1 and produced obviously wrong structures for ATPase3 and *Plasmodium* subtype-P5A ATPase.

Subtype-P5A and subtype-P5B also differ in the substrate binding groove with subtype-5A having a substantially wider groove to accommodate the α-helix substrate. Swiss Model generated a substrate-binding groove that reflected the template rather than the sequence being modeled (Figure 9). Modeling with AlphaFold only generated narrow substrate-binding grooves that ranged from 4.6 to 6.5 Å (Table 6, Figure S6c,d). This is likely due to most P-type ATPases having narrow substrate-binding grooves, and type-P5A ATPases are exceptions.

Swiss Model also generated some discrepancies in the P-domain especially from ATPase1 and ATPase3 (Figure 6, Figure 7 and Figure 8). The P-domain structures of Spf1 and ATP13A2 generated by AlphaFold were very similar to the experimentally determined structures and the homology models produced with concordant templates, including the lack of helix-3 in ATP13A2 (Figure S6e). The *Plasmodium* subtype-P5A structures generated by AlphaFold were very similar to Spf1 and the structures generated by Swiss Model (Figure 6) and likely represent correct structures. The AlphaFold-predicted structures of ATPase1 and ATPase3 resembled the P-domain of ATP13A2, but some of the secondary structural elements were generated from the variable region 4 sequence and exhibited some notable variations from the ATP13A2 P-domain structure (Figure S6e). Notably, there were helices resembling the helix-3 of the subtype-P5A that were generated by variable sequence. The nearby β-strands and sometimes helix-4a are also generated from variable sequences. In addition, these secondary structural elements generated from the variable regions were longer than those from ATP13A2. Removal of the variable regions before the modeling did not correct this error and resulted in these structures not being generated (Table 6). This is in contrast to the homology modeling, where removal of the variable regions partially corrected the discrepancies in the P-domain (Table 5).

## 4. Discussion

Type-P5 ATPases are not well characterized, and this is especially true for the type-P5 ATPases of the malaria parasite. Malaria parasites, like most other eukaryotes, have two type-P5 ATPases, designated as subtype-P5A and subtype-P5B. The subtype-P5A gene and protein of *Plasmodium* have not yet been characterized other than to be listed as part of genomic surveys [2] or phylogenetic studies [12]. Two subtype-P5B ATPases have been identified in the major human pathogen *Plasmodium falciparum* and designated as ATPase1 and ATPase3. ATPase1 is not found in all species of malaria parasites and is limited to the parasites of great apes (i.e., *Laverania*), avian malaria parasites, and *Haemoproteus* [13]. ATPase1 arose from a duplication of the ATPase3 gene early in the evolution of the malaria parasite. This further characterization of the type-P5 ATPases from the malaria parasite provided insight into the structures of the *Plasmodium* type-P5 ATPases, revealed a possible role for the IDL, allowed for speculation about the possible functions of type-P5 ATPases in the malaria parasite, and possibly contributed to a better understanding of the evolution of the type-P5 ATPases.

### 4.1. Homology Modeling and Predicted Structures

Sequence alignments and homology modeling of the *Plasmodium* type-P5 ATPases identified all the expected canonical domains found in P-type ATPases (i.e., A, N, P, and M). The predicted secondary structures of these canonical domains recapitulated experimentally determined structures with relatively minor discrepancies, whether concordant (i.e., same subtype) or discordant (i.e., different subtype) templates were used in homology modeling. Many of these discrepancies disappeared if the regions corresponding to the IDL were removed from the sequences before modeling. This suggests that large intrinsically disorganized regions may interfere with homology modeling. Even though this interference is minor, it may be prudent to carry out modeling on both complete protein sequences and sequences with large regions of low-complexity sequence removed. This may be especially important for proteins of the malaria parasite since large regions of low-complexity sequence are often found in *Plasmodium* proteins [15]. In contrast to the canonical P-type ATPase domains, the NTD and CTE were not always accurately modeled, and often the N-terminal and C-terminal regions were excluded from the predicted three-dimensional structures.

The secondary structural features of the A- and N-domains were nearly identical between the subtype-P5A and subtype-P5B ATPases whether concordant or discordant templates were used. This emphasizes the similarity between the two subtypes of the type-P5 ATPases. Overall, homology modeling predicted structures that recapitulated the known structures of type-P5 ATPases and it is likely that the subtype-P5A proteins from *Plasmodium* have a similar structure to other subtype-P5A ATPases. Likewise, ATPase1 and ATPase3 have similar structures to other subtype-P5B ATPases. The good performance of homology modeling also indicates that homology modeling will be a useful tool in future studies of these proteins and may be useful in preliminary studies to search for possible drugs targeting P-type ATPases.

Differences between subtype-P5A and subtype-P5B ATPases are primarily in the membrane associated helices of the NTD, the arm of the P-domain, and the substrate specificity [8]. For example, the subtype-P5A NTD has two additional transmembrane helices and the subtype-P5B NTD has a triangular membrane loop that does not traverse the lipid bilayer. Homology modeling of the *Plasmodium* type-P5 ATPases did correctly generate these structures when concordant templates were used and especially if the variable regions were removed from the protein sequences. Another difference between subtype-P5A and subtype-P5B is an a-helical projection in the P-domain called the arm that is not found in subtype-P5B. As expected, no arm structures are found in ATPase1 or ATPase3 and this arm was predicted in the PrP5A sequence with the Spf1 (i.e., concordant) template and not the ATP13A2 (i.e., discordant) template. It is not clear why the arm was not predicted in PfP5A.

An extra α-helix was observed in the Rossmann fold of the P-domain in the subtype-P5A ATPases, which was not seen in the subtype-P5B ATPases. This extra helix may possibly be another structural feature to distinguish subtype-P5A and subtype-P5B ATPases and merits further investigation. To accommodate their distinct substrates, the substrate-binding groove of Spf1 is much wider than the narrow groove of ATP13A2.

### 4.2. Limitations of Homology Modeling

Discordant templates revealed a potential limitation of homology modeling since the predicted three-dimensional structures had a strong tendency to recapitulate the template, especially in the NTD and substrate-binding grove. For example, homology modeling of the *Plasmodium* subtype-P5A ATPase with the subtype-P5B template (i.e., discordant) generated the triangular membrane loop, whereas modeling with the concordant template produced the expected transmembrane helices. This same effect of the predicted structures reflecting the templates was also observed in the *Plasmodium* subtype-P5B ATPases. However, in the case of the NTD the concordant templates did produce structures of slightly higher quality in regard to recapitulating the experimentally determined structures than the discordant templates. This was not true with the substrate-binding groove. The homology models were essentially identical to the experimentally determined structures of the templates and both concordant and discordant templates appeared to generate structures of equal quality. Thus, it will be important to use the correct template when carrying out homology modeling and to have a means to choose the correct template among multiple possible templates. For example, the Spf1 template ranked equally well to subtype-P5B templates in an analysis of subtype-P5B ATPases using Phyre^®^ [13].

Modeling with AlphaFold was carried out to potentially resolve biases generated by templates. Overall, AlphaFold generated structures that were similar to Swiss Model, especially with Spf1 and ATP13A2. However, the structures of the *Plasmodium* ATPases predicted by AlphaFold tended to be inferior to the structures predicted by the Swiss Model. For example, the IDL often were predicted as a-helices that sometimes appeared to traverse the membrane. This limitation is referred to as a hallucination [30]. AlphaFold did predict the N-terminal membrane loops that the Swiss Model could not. However, in the *Plasmodium* subtype-P5A ATPases and ATPase3 these structures were obviously wrong. Likewise, AlphaFold failed to predict the extra wide substrate-binding groove of the subtype-P5A ATPases and did not correct for the discrepancies in the P-domain of subtype-P5B ATPases.

Another limitation is that most experimental work on eukaryotes has been carried out in humans and other mammals or yeasts. In terms of eukaryotic diversity, fungi and metazoans are relatively closely related as members of the opisthokonts within a major eukaryotic supergroup, or clade, that includes some amoeba called Amorphea [38]. Little experimental work has been carried out in the other major eukaryotic supergroups other than perhaps plants and a smattering of pathogenic eukaryotes. It is not inconceivable that even highly conserved proteins may exhibit structural or functional differences between the major eukaryotic supergroups. In regard to this study, experimentally determined structures from other apicomplexans or members of SAR supergroup, which represents half of all eukaryote diversity [39], may be informative and useful as templates. Thus, more experimental data reflecting the extreme diversity of eukaryotes is clearly needed.

### 4.3. Possible Functions of IDL

An obvious unique feature of the predicted structures of the *Plasmodium* type-P5 ATPases is the presence of large loops of intrinsically disorganized sequence. Intrinsically disorganized regions in proteins are random coil secondary structures that lack bulky hydrophobic amino acids and do not adopt a tertiary structure with a hydrophobic core [40,41]. Traditionally, IDL were viewed as passive linkers between structured domains, but they are now known to have functional roles [42]. For example, IDL can be involved in the formation of protein complexes and higher-order supramolecular structures [43]. The flexibility of the IDL allows them to take on many conformations and may allow for an induced fit as they bind ligands, including other proteins. Thus, the IDL may allow for low-affinity interactions with other proteins. At the same time, the physical mass of the IDL, which ranges from one-third to one-half in the type-P5 ATPases of *Plasmodium*, could also provide spacing between proteins.

The flexibility of the IDL could also form a shroud-like structure that surrounds the core ATPase structure. Such a shroud could have a protective role and prevent damage to the primary domains. Similarly, such a shroud may provide a buffer zone for these ATPase molecules so that other proteins on the membrane do not inadvertently interact with them and thus possibly interfere with their function. A protective role of the shroud and a role in protein–protein interactions are not necessarily mutually exclusive.

The IDL are correlated with inserts of variable regions which intuitively would argue against a specific function. However, these variable regions only exhibit variation between the eight haemosporidian clades and are conserved within the individual haemosporidian clades for both subtype-P5A (Figure S1) and subtype-P5B [13]. These variable regions are composed of low-complexity sequences that are highly enriched in polar amino acids, as are IDL in general [40,41]. Thus, despite the lack of sequence conservation, the flexibility and similar amino acid composition of the IDL could preserve a function such as low-affinity binding to other proteins. Indeed, low-complexity sequences have been demonstrated to be subject to natural selection [44]. The observation that tandem repeats are often observed in these low-complexity regions suggests a mechanism to explain the variability and preservation of the amino acid composition. A periodic replacement and expansion of tandem repeats through slipped-strand mispairing [45] or unequal crossing-over [46] could occur on a similar evolutionary timeframe as the formation of the major haemosporidian clades.

### 4.4. Substrate Specificity

The substrate specificity of P-type ATPases and their subcellular locations are key elements regarding their functions [8]. Subtype-P5A ATPases are localized to the ER [47] and have been previously hypothesized to transport calcium [48], manganese [49], or lipids [50]. Recent studies indicate that Spf1 is likely a transmembrane helix dislocase [8,11]. Presumably this dislocase activity serves as a quality-control mechanism to remove mistargeted transmembrane helices from the ER membrane. Consistent with this function, a subtype-P5A ATPase from *Caenorhabditis elegans* plays a role in correctly targeting membrane and secretory proteins [51]. Other than some early speculation that subtype-P5B might be heavy metal transporters [52], most studies have characterized subtype-P5B ATPases as polyamine transporters of late endosomes or lysosomes [10,53,54,55,56].

Homology modeling did not resolve the substrate specificity of the *Plasmodium* type-P5 ATPases since the substrate-binding sites conform to the template, and modeling with AlphaFold did not resolve this problem. Nonetheless, it is quite likely that *Plasmodium* subtype-P5A ATPases are helix dislocases. This assertion is supported by the observation that the key residues for binding to the α-helix substrate are conserved, whereas key residues involved in polyamine binding are less conserved (Figure 9). Helix dislocase activity is likely a necessary quality-control mechanism in the ER and disruption of the gene has adverse pleiotropic effects [8,57]. The subtype-P5A gene is found in all eukaryotes and with few exceptions it is a single copy gene [58]. It is also believed that subtype-P5A may have a general and highly conserved function [47]. The very high level of sequence conservation, except for the variable region inserts, within the haemosporidians (Figure S1), and identification of clear orthologues throughout the SAR (Table S1) further support a conserved function of the subtype-P5A ATPases in all eukaryotes, including the malaria parasite.

In contrast, as previously discussed [13], it is unlikely that the haemosporidian subtype-P5B ATPases are lysosomal polyamine transporters. Previously published immunofluorescence data [17,18,19] are more reminiscent of ER staining, and proteomic analysis did not detect any P-type ATPases in the lysosomal compartment (i.e., digestive vacuole) of the malaria parasite [59]. Furthermore, malaria parasites are capable of synthesizing polyamines [60] and have a plasma membrane-associated polyamine transporter to take up polyamines from the host cell cytoplasm [61]. In addition, clear orthologues of ATPase3 can only be identified in the apicomplexans and ATPase1 is limited to only some of the malaria parasites [13]. If the apicomplexan and opisthokont subtype-P5B ATPases had the same function, one might expect to be able to identify clear orthologues throughout the eukaryotes. Determining the substrate specificity of the haemosporidian or apicomplexan subtype-P5B ATPases will likely require experimental verification.

### 4.5. Divergent Evolution of Subtype-P5B ATPases

Type-P5 ATPases likely originated during the early evolution of eukaryotes, and its origin was likely coincident with the formation of the ER and other internal membrane systems [12,58]. The duplication and divergence into subtype-P5A and subtype-P5B also likely occurred in a primordial eukaryote before the formation of the major eukaryote supergroups. Only a limited number of subsequent duplications of subtype-P5A have been noted in the archaeplastids and stramenopiles [58]. In contrast, subtype-P5B has been duplicated many times in several different eukaryotic lineages [8,12,13]. In addition, subtype-P5B is not found in all eukaryotes, and this is usually attributed to lineage-specific losses of subtype-P5B [12]. For example, gene loss certainly explains the lack of subtype-P5B in *Entamoeba* since these organisms are amorpheans, as are fungi and metazoans, which both have subtype-P5B. This loss of the subtype-P5B gene likely occurred after the amoebozoans split from the opisthokonts.

Duplicated genes can undergo divergent evolution, and this allows for an expansion and diversification of gene families and provides opportunities for innovation and adaptation to new environments or physiological milieus [62]. Following the split into subtype-P5A and subtype-P5B, it is probable that subtype-P5A maintained its function as an ER helix dislocase in all eukaryotes. Subtype-P5B, on the other hand, may have evolved new functions in a lineage-specific manner. For example, subtype-P5B in the opisthokonts diverged into a polyamine transporter located in the lysosome. In contrast to the opisthokonts, the subtype-P5B ATPases of the malaria parasite appear to have maintained their location in the ER and did not evolve to transport polyamines. Therefore, it is unlikely that subtype-P5B ATPases have a common function in all eukaryotes, and there could be lineage-specific functions in the various eukaryotic supergroups.

The retention of subtype-P5B ATPase in the ER of malaria parasites implies a possible retention and localization of subtype-P5B ATPases to the ER throughout the Apicomplexa, and even perhaps the SAR supergroup. This could represent redundancy or perhaps a divergence to a more specialized function. Furthermore, homology modeling with the discordant subtype-P5A template indicates that ATPase1 and ATPase3 can, at least in theory, be capable of helix dislocase activity. In addition, the observation that the *Plasmodium* subtype-P5B ATPases are approximately equal distance from Spf1 and ATP13A2 in phylogenetic pairwise analyses (Table 3) is also consistent with *Plasmodium* subtype-P5B ATPases having a similar function as subtype-P5A ATPases. Similarly, the quality assessment scores of *Plasmodium* subtype-P5B ATPases are approximately the same, with concordant or discordant templates suggesting no strong preference for either template. In contrast, the quality scores of subtype-P5A ATPases show a preference for concordant templates over discordant templates. Therefore, it is not inconceivable that the subtype-P5B ATPases of *Plasmodium*, other apicomplexans and members of SAR have a function similar to the subtype-P5A ATPases.

Maintenance of two genes with similar functions allows for divergence in regard to the precise substrate specificity. For example, subtype-P5A ATPases have also been implicated in the removal of signal peptides from the ER membrane [8]. Interestingly, the malaria parasite and other apicomplexans may have two distinct pathways targeting proteins for export [63]. One of these pathways involves the traditional signal peptide and the other involves a signal called PEXEL [64]. PEXEL signaling is also found in stramenopiles [65]. As a highly hypothetical speculation, one could propose the need for different helix dislocases to remove these rather different peptide signals from the ER membrane. These secretory pathways of the malaria parasite are involved in the export of virulence factors to the host erythrocyte and thus contribute to the pathogenesis of malaria [66]. Therefore, drugs targeting the type-P5 ATPases of the malaria parasite may be particularly effective.

## 5. Conclusions

The subtype-P5A ATPase of malaria parasites is likely a helix dislocase of the ER as are other subtype-P5A ATPases. In contrast, the subtype-P5B ATPases of the malaria parasite have likely diverged from other subtype-P5B ATPases and are not polyamine transporters of the lysosome. The experimental verification of cellular locations and substrate specificities of the type-P5 ATPases of the malaria are needed. A notable difference between the type-P5 ATPases of the malaria parasite and other subtype-P5A ATPases is the insertion of variable regions composed of low-complexity sequence. This low-complexity sequence may form a shroud that surrounds the core of the ATPase, which may function in low-affinity protein–protein interactions or protection of the core ATPase.

## Figures and Tables

**Table 1:** Sequences used in detailed sequence and structure analysis.

Protein	Species	Abbr	Gene ID	Activity/Description	Ref.
Spf1	*Saccharomyces cerevisiae*	Spf1	AAB64508.1	Transmembrane helix dislocase	[9]
Uncharacterized subtype-P5A	*Plasmodium falciparum*	PfP5A	PF3D7_0727800	Only identified in sequence databases	[12,2]
Uncharacterized subtype-P5A	*Plasmodium* *relictum*	PrP5A	PRELSG_0216200		
ATP13A2	*Homo sapiens*	13A2	NP_071372.1	Polyamine transporter	[11]
ATPase3	*Plasmodium falciparum*	PfA3	PF3D7_0504000	Apicomplexan subtype-P5B of unknown substrate specificity	[13]
ATPase3	*Plasmodium* *relictum*	PrA3	PRELSG_1028500		
ATPase1	*Plasmodium falciparum*	PfA1	PF3D7_0516100	Paralogue of ATPase3 only found in *Laverania* and avian Haemosporida	
ATPase1	*Plasmodium* *relictum*	PrA1	PRELSG_1015800		

**Table 2:** Sizes and locations of low-complexity inserts in type-P5 ATPases ^1^.

Domain	NTE	NTD	A1	A2	N1	N2	P1	P2	Total
P5B-VR		VR1		VR2	VR3		VR4		
13A2	n.a.	23	0	0	0	0	56	0	79 (7%)
PfA1	n.a.	126	0	168	281	37	664	0	1276 (53%)
PrA1	n.a.	45	0	136	145	0	681	0	1007 (48%)
PfA3	0	288	0	127	478	0	327	0	1220 (51%)
PrA3	0	89	0	52	332	0	280	0	753 (39%)
P5A-VR	VR1	VR2	VR3	VR4		VR5	VR6	VR7	
PfP5A	79	25	180	107	2	147	158	103	801 (42%)
PrP5A	79	24	119	15	2	43	152	91	525 (32%)
Spf1	n.a.	0	0	0	0	0	65	119	184 (15%)

^1^ The variable regions of subtype P5A and subtype P5B ATPases are colored green and blue, respectively, to match Figure 1. Between the NTE and NTD there is aninsert (VR1), which is only found in subtype-P5A. The insert in the NTD is in the middle of an anti-parallel β-sheet and corresponds to variable region 1 (VR1) of subtype-P5B and VR2 of subtype-P5A. The first insert in the A-domain (A1) is located near the NTD/A-domain junction and is specific to subtype-P5A. The second A-domain insert (A2) is located between β-strand-5 and β-strand-6 of the distorted jelly roll. The first insert in the N-domain (N1) is VR3 of subtype-P5B and is located near the first P-domain/N-domain junction. Another N-domain insert is found between β-strand-3 and β-strand-4 of the twisted β-sheet of the N-domain (N2). The first insert in the P-domain (P1) is located between β-strand-3 and helix-3 of the Rossmann fold. The second P-domain insert (P2) is located after the ‘arm’ that is exclusive to subtype-P5A. Shown are the sizes of the variable regions, expressed as number of amino acids and the percentage of the total amino acids found in the variable regions. n.a. = not applicable.

**Table 3:** Pairwise distances between *Plasmodium* type-P5 ATPases, Spf1 (subtype-P5A), and ATP13A2 (subtype-P5B) ^1^.

	13A2	PfA1	PrA1	PfA3	PrA3	Pf5A	Pr5A
PfA1	1.32						
PrA1	1.24	0.42					
PfA3	1.49	1.39	1.41				
PrA3	1.56	1.40	1.40	0.13			
Pf5A	1.43	1.37	1.38	1.51	1.47		
Pr5A	1.45	1.36	1.40	1.48	1.44	0.25	
Spf1	1.39	1.44	1.41	1.59	1.58	**1.14**	**1.16**

^1^ Pairwise distances were generated using Mega XI from a ClustalW alignment with the variable regions removed. Concordant alignments are shaded in green and discordant alignments are shaded in red. The comparison of orthologues from *P. falciparum* and *P. relictum* is boxed. The distance between the Spf1 and ATP13A2 templates is underlined and the distances between the *Plasmodium* subtype-P5A ATPases and Spf1 are in bold.

**Table 4:** Quality assessment of the predicted three-dimensional structures of Plasmodium type-P5 ATPases ^1^.

	6xmu (aHS, BeF, Mg)				6xmq (ACP, Mg)				7m5x (spm, BeF, Mg)				7m5v (ANP, Mg)			
ATPase	GM	QM	RF	Ligand	GM	QM	RF	Ligand	GM	QM	RF	Ligand	GM	QM	RF	Ligand
Spf1	0.72	0.78	92.4	Mg	0.78	0.82	95.5	ACP, Mg	0.47	0.55	86.4	Mg	0.50	0.59	86.6	Mg
ATP13A2	0.54	0.59	88.9	Mg	0.54	0.60	89.4	Mg	0.67	0.71	91.9	BeF, Mg	0.69	0.74	91.8	ANP, Mg
PfP5A	0.27	0.50	81.6	BeF, Mg	0.28	0.50	80.2	Mg	0.19	0.46	82.9	BeF, Mg	0.20	0.46	80.6	Mg
PrP5A	0.39	0.51	83.5	Mg	0.39	0.50	84.1	Mg	0.27	0.48	82.9	Mg	0.29	0.51	86.9	Mg
PfA1	0.12	0.37	73.8	Mg	0.13	0.38	73.7	Mg	0.11	0.38	69.4	BeF, Mg	0.12	0.39	72.2	-
PrA1	0.20	0.38	76.0	Mg	0.18	0.38	76.7	-	0.18	0.38	72.7	Mg	0.18	0.38	73.0	Mg
PfA3	0.15	0.41	75.5	Mg	0.15	0.41	74.0	Mg	0.13	0.41	72.9	Mg	0.14	0.43	75.4	Mg
PrA3	0.24	0.41	79.3	Mg	0.24	0.41	78.3	Mg	0.21	0.41	77.2	Mg	0.21	0.42	78.0	Mg
PfP5Avrr	0.54	0.58	91.4	Mg	0.54	0.58	91.6	Mg	not analyzed							
PrP5Avrr	0.54	0.59	91.2	Mg	0.54	0.59	91.2	Mg								
PfA1vrr	not analyzed								0.49	0.53	89.6	BeF, Mg	0.51	0.55	91.0	-
PrA1vrr									0.52	0.56	87.1	BeF, Mg	0.54	0.56	90.4	-
PfA3vrr									0.46	0.52	86.7	Mg	0.47	0.53	89.3	Mg
PrA3vrr									0.49	0.52	88.0	Mg	0.49	0.53	89.7	Mg

^1^ The top row shows the PDB files used as templates to model the sequences in the first column. The features in the parentheses () are the ligands associated with the crystallized protein. Ligands include an α-helix substrate (aHS), spermine (spm), non-hydrolysable ATP analogs (either adenosine 5′-[β,γ-methylene]triphosphate, ACP, or β,γ-imidoadenosine 5′-triphosphate, ANP), BeF_3_ (mimics the phosphorylated state), or Mg^2+^. The second row are the quality assessments and include GMQE (GM), QMEANDisCo (QM), percentage of Ramachandran favorable ψ and Θ angles (RF), and the ligands retained in the model. Dashes (-) indicate no ligand was retained in the model. Concordance or discordance with the prototype ATPases are denoted with light green or red shading, respectively. For the *Plasmodium* ATPases concordance and discordance are denoted with blue and gray shading, respectively. An analysis of the *Plasmodium* type-P5 ATPases with the variable regions removed (vrr) was also carried out with concordant templates.

**Table 5:** The effect of removing variable regions on discrepancies in the modeling of *Plasmodium* subtype-P5B ATPases ^1^.

			6xmu Template (Discordant)			7m5x Template (Concordant)		
Domain	ATPase	VR	Discrepancy	VRR Effect		Discrepancy	VRR Effect	
NTD (VR1)	PfA1	126	missing NTD	partial restoration of nTM2	+	missing NTD	restoration of nML	+
	PrA1	45	missing nTM1	none	0	none	none	0
	PfA3	288	missing NTD	missing NTD	0	missing NTD	partial restoration of nML	+
	PrA3	89	missing NTD	partial restoration of nTM2	+	missing NTD	partial restoration of nML	+
A (VR2)	PfA1	168	none	none	0	extra β-strand	loss of extra β-strand	+
	PrA1	136	none	none	0	none	none	0
	PfA3	127	none	none	0	none	none	0
	PrA3	52	none	none	0	none	none	0
N (VR3)	PfA1	278	none	none	0	none	none	0
	PrA1	153	none	none	0	none	none	0
	PfA3	469	none	none	0	none	none	0
	PrA3	323	none	none	0	none	none	0
P (VR4)	PfA1	558	b4 and b5 missing, extra helix derived from VR4	b4 and b5 restored, loss of extra helix	+	b4 and h3 generated from VR4, extra β-strand derived from VR4	b4 and h3 generated from expected sequence, loss of extra β-strand	+
	PrA1	568	extra β-strand derived from VR4	loss of extra β-strand	+	none	none	0
	PfA3	217	b3 and b4 missing	b3 and b4 restored	+	b3 generated from VR4, extra β-strand derived from VR4	b3 generated from expected sequence, loss of extra β-strand	+
	PrA3	172	none	b3 missing	−	b3 missing	additional loss of b4 and b5	−

^1^ Based on Figure S5, Figure 3, Figure 4, Figure 5, Figure 6, Figure 7 and Figure 8. The b# and h# refer to specific β-strands and a-helices, respectively, found in the domains. VR = sizes of variable regions in that domain. The effects of variable regions are rated as positive (+) if removing the variable regions improved the predicted structure in regard to recapitulating the experimentally determined structures, neutral (0) if removing the variable regions had no effect, and negative (−) if removing the variable regions led to a loss of expected secondary structures.

**Table 6:** Summary of AlphaFold modeling results ^1^.

	Score	pTM	%Dis	IDL Hallucination	Arm	nML	SBG	P-Domain
Spf1	0.88	0.86	0.03	Moderate	Full helix	Expected structure	4.9 Å	Expected structure
Pf5A	0.80	0.65	0.31	Extensive	Partial helix	Extracytoplasmic IDL	4.6 Å	Expected structure
Pr5A	0.83	0.74	0.19	Extensive	Quasi-helix	Extracytoplasmic IDL	6.5 Å	Expected structure
ATP13A2	0.88	0.84	0.08	Minor	n.a.	Expected structure	6.0 Å	Expected structure
PfA1	0.78	0.56	0.43	Extensive	n.a.	Expected structure	5.6 Å	Partially generated from VR4
PfA1_vrr	0.87	0.83	0.06	Minor	n.a.	Expected structure	6.1 Å	Missing structural elements
PrA1	0.81	0.61	0.41	Extensive	n.a.	Expected structure	5.9 Å	Partially generated from VR4
PrA1_vrr	0.87	0.83	0.09	Moderate	n.a.	Expected structure	6.3 Å	Missing structural elements
PfA3	0.81	0.57	0.47	Extensive with a TM helix	n.a.	Two transmembrane helices instead of triangular loop	5.9 Å	Partially generated from VR4
PfA3_vrr	0.89	0.86	0.05	Minor with a TM helix	n.a.	Two transmembrane helices instead of triangular loop	5.9 Å	Missing structural elements
PrA3	0.83	0.65	0.35	Extensive with a TM helix	n.a.	Two transmembrane helices instead of triangular loop	5.7 Å	Partially generated from VR4
PrA3_vrr	0.89	0.86	0.06	Minor with a TM helix	n.a.	Two transmembrane helices instead of triangular loop	6.0 Å	Missing structural elements

^1^ Based on Figure S6. Shown are the ranking score, pTM score, and percent disordered (%Dis) sequence calculated by AlphaFold3. Abbreviations: n.a. = not applicable; vrr = variable region removed; TM = transmembrane; nML = N-terminal membrane loops; SBG = substrate-binding groove width.

## Data Availability

The raw data supporting the conclusions of this article will be made available by the authors on request.

## Supplementary Materials

The following supporting information can be downloaded at: https://www.mdpi.com/article/10.3390/pathogens14111164/s1, Table S1: Identification of subtype-P5A ATPase genes in SAR (Stramenopiles, Alveolata, and Rhizaria); Figure S1: Alignment and phylogeny of subtype-P5A ATPases from haemosporidians; Figure S2: Alignment of *Plasmodium* type-P5 ATPases with Spf1 (type-P5A) and 13A2 (type-P5B); Figure S3: A-domain structure of type-P5 ATPases; Figure S4: N-domain structure of type-P5 ATPases; Figure S5. Effects of low-complexity variable regions on homology modeling; Figure S6. Modeling with AphaFold.

## Abbreviations

The following abbreviations are used in this manuscript:

cTM	Core transmembrane helix
CTE	C-terminal extension
IDL	Intrinsically disorganized loops
PEXEL	Plasmodium export element
PlP5A	Plasmodium subtype-P5A ATPase
SAR	Stramenopiles–alveolates–rhizarians
SERCA	Sarcoplasmic–endoplasmic reticulum ATPase
