# Supplementary material for: Homology Modeling of Type-P5 ATPases from the Malaria Parasite: Insight into Their Functions and Evolution, and Implications About the Effect and Role of Intrinsically Disordered Protein Structure
Source: Pathogens. 2025 Nov 14;14(11):1164. doi: 10.3390/pathogens14111164 (PMC12655044; doi:10.3390/pathogens14111164)
Supplement: Supplementary file 1 [file pathogens-14-01164-s001.zip › Supplemental Figure S1.pdf]

Supplemental Figure S1. Alignment and phylogeny of type-P5A ATPases from haemosporidians.

(a)

```
                      N-terminal extension (NTE)                     <-|->    variable region-1 (VR1)
HtarP5A MKDKYRVRIYAQKKRYKRLDVLLFFFYIFFLNFILKNKNFEAKPSDYEYLKLLEIRRIEEENAKQLDTENLTDFNELSEVERFEDEDTDRE-----------------------------
PrelP5A MKNKYNIIIYKKRKTHCRLDVLLFLFYAFYLNFILQNKKFEAQPSDYEYIEKLKLKKS-EEINILKFNNSNLSSDELLYDESEEEITEKLDG----------------------------
PgalP5A MNNKYNVIIYKKRKTHCRLDVLLFLFYAFYLNFILQNKKFEAQLSDYEYIERLKLEKI-EEENIFKFNNSNISSDKLIYEENGEEITENLDG----------------------------
PfalP5A MRSKYNILIYKKKKKHLRLDVLLFFVYLIFLNLILQNKKFEAQPKDYEYIEILKNKNVNEETFVHVKRFEPTSS--DLGNIGKEKILE--------------------------------
PpraP5A MRSKYNILIYKKKKKHLRLDVLLFFVYLIFLNLILQNKKFEAQPKDYEYIEILKNKNVNEETFVHVKRFEPTSS--DLGNIGKEKILE--------------------------------
PreiP5A MRSKYNILIYKKKKKHLRLDVLLFFVYLIFLNLILQNKKFEAQRKDYEYIEILKNKNVNEETFVHVKRFEPTSS--DLGSIGKEDVLE--------------------------------
PadlP5A MSSKYNILIYKKKKKHLRLDVLLFFFYLIFLNLILQNKKFEAQQKDYEYIENLKIKNVEEEGFVHVKKFDSSSSSYNFQDIGNEDVLEEDNNNI--------------------------
PbilP5A MRSKYNILIYKKKKKHLRLDVLLFFFYLIFLNIILQNKKFEAQVKDYEYIEILKNKNGNEEDFVRVKRYEASSS--DFGNIGKEEVLE--------------------------------
PblaP5A MRSKYNILIYKKKKKHLRLDVLLFLFYLIFLNLILQNKKFEAQLKDYEYIEILKNTNVNEEVFVHVKRFDSSSSS-DLRNVGKEELLEGNDNNIN-------------------------
PgabP5A MSSKYNILIYKKKKKHLRLDVLLFFFYLIFLNLILQNKKFEAQQKDYEYIENLKTKNVKDEGFVHVKKFDSSSSSYKFEDIGNEDVLEDNNNNNN-------------------------
HsppP5A MKNKYNIFIYKKRKTHLRLDVLLFLFYAFFAHLILYNKKFEAQPADYEYIEKLKNAVE-ENIVFNNKNTDIISGIKWFEEDDKEETDVKGDKEKTDVKDDSSFYMVNGKGEVNEKTGIES
PberP5A MERKYNILAYKKRKTYFRLDVLLFIFYAYFLHLIFKNKKFEAQPEDYEYFEKLKTELD-NNDIFHSNN-NSFSTSKWLYGKNNEND----------------------------------
PyoeP5A MERKYNILAYKKRKTYFRLDVLLFIFYAYFLHLIFKNKKFEAQPKDYKYLEKIKTELE-NNDIFHSKN-NSFSTSKWLYG---END----------------------------------
PchaP5A MERKYNVLAYKKRKTYLRLDVLLFIFYAYFLHLIFKNQKFEARPEDYEYLEKLKIELD-NNQIFNSKN-DSHSANKWIYD---KNN----------------------------------
PvinP5A MERKYNVLAYKKRKRYLRLDVLLFIFYAYFLHLILKNQKFEARPEDYEYLEKLKTELD-NNEIFNSKN-ISHSASKWIYD---KNN----------------------------------
PgonP5A MKHKYNVLIYRKKKTHLRLDVLLFFFYTVFIHLILQNEKFEAQPSDYEYLANLQNEIEETNIYNLNEAFKPPYSPDSYVYKKNKDEKEQQ------------------------------
PvivP5A MKHKYHVLIYRKKKTHLRLDVLLFFFYAVFLHFILKNEKFQAKPGDYEYLAKLQNEVEGVEGVEGVEAAEAAAVFGLHGEGQRQEKRQ--------------------------------
PcoaP5A MKHKYHVLIYRKKKTHFRLDVLLFFFYAYFLHLILKNEKFHAKPGDYEYLAKLENEVE--EAVMFGLHGEEQQQ-------RVGGNER--------------------------------
PcynP5A MKHKYHVLIYRKKKTHLRLDVLLFFFYAVFLHLFLKNEKFQAKPSDYEYLEKLQNEVE--EAVMFGLHGEEEQQ-QTFGELKVGASER--------------------------------
PfraP5A MKHKYHVLIYRKKKTHFRLDVLLFFFYAVFLHLILNNEKFQATPGDYEYLAKLQNEVE--EEVMFGLQGDHPN-----GEFKVGTSGR--------------------------------
PinuP5A MKHKYHVLIYRKKKTHLRLDVLLFFFYAVFLHFILKNDKFEAKPGDYEYLAKLQNEVE--EAFMFGLH-EEEKQ-KPLGELKVGASER--------------------------------
PknoP5A MKHKYHVLIYRKKKTHLRLDVLLFFFYAVFLHFILKNEKFYAKPGDYEYLAKLENEVE--EAVTFGLHGEEQKQS--LDERKVEGSES--------------------------------
PbraP5A MKSKYNMQIYRKRKTHLRLDVLLFFFYVVFLNLILQNKKFEAQPSDYEYIEKSKNEIKEENIFKFKNSIDSSDQLKHEQNMKEHEDQLYNELEVGKNEI---------------------
PmalP5A MKSKYNVQIYRKRKTHLRLDVLLFFFYVVFLNLILQNKKFEAQPSDYEYIEKSKNEIKEENIFKFKNSIDSSDQLKHEQNMKEHEDQLYNELEVGKNEI---------------------
PovCP5A MKSKYNVLIYRKRKTHLRLDVLLFFFYAFFLHLILKNKKFEAKPSDYAYIEKIKNETEQANFFTFDKASDSVNAPLHGQHGDVNTDEENLDKLKIGDDKM--------------------
PovWP5A MKSKYNVLIYRKRKTHLRLDVLLFFFYAFFLHLILKNKKFEAKPSDYAYIEKIKNETEQVNFFTFDEASDPVNAPLHGQHGDVNKDEENLDKLKVGDDKI--------------------
```

```
                                                                                    VR1       <-|->  nTM1   <-|     |->  nTM2
HtarP5A -------------------REIGDVPSSTFTSTDGSAISIDSS-----------------------TNKLNKEKSEGHSDFGEDQSVPIIIGKYNLIYILFSVEFVVLLVFILFHIAVFL
PrelP5A --------------------IKIRVNELEKNHINTTDKNINDV--------NISTENNGNDINISTENNGNDEQELLKENDEETQLKPILLGKYNLIYIFYSIEFVCLLLFIFFHMMVFL
PgalP5A --------------------IKIQMDNLEREHENTNYKIVN-----------------NEQNIYIDNNKNDKEKILKDKEEEETQLKPIILGKYNLIYIFYSIEFLCLLLFIFFHLMIFL
PfalP5A -------------------QNNYNVKSINNVELRNDNMNVIN----NEGVYNKYVKENEEIIGDNEKKEKRAKTFENNDFKNEKSFKPIIVGKYNLIYIFYSIEFICLLLFIFLHFLLFL
PpraP5A -------------------KNNYNVKSINNIELRNDNMNVIN----NEGVYNKNVKENEEIIGDNEKKEKRAKTFENNDFKNEKSFKPIIVGKYNLIYIFYSIEFICLLLFIFLHFLLFL
PreiP5A -------------------ENNYNVKSINNIERRNDNMNVVN----NEGVYNKYIKENEEIIGDNEKKEKRKKTFENNDFKNEKSFKPIIVGKYNLIYIFYSIEFICLLLFIFLHFLLFL
PadlP5A -KSIKSINNIKSINNIKSIKSINNIKSINNGEPRNDNINVVN----NKNKYNKSLKENEEIVGGSETNENKKKTFESEDLKNEKSFKPIIIGKYNLIYIFYSIEFICLLLFICLHVLLFL
PbilP5A -------------------ENNYNIKSINNMEPKNDNMNATN----NDKIYDKNVKENEKIIGNNEKKAKKKKTFDNDDFKNEKSFKPIIIGKYNLVYIFYSIEFICLLLFIFLHFLLFL
PblaP5A -----------SI-NKNAYRNDNNIKSINKNESRNDNINVINNIVNNENIYDKNVKENEEVNGDNEKKEKKKKTFDNNDFKNEKSFKPIIIGKYNLIYIFYSIEFICLLLFIFLHFLLFL
PgabP5A --------------------NNNNIKSINNVEARNDNMHVVN----NMNKYDKSLKENEKIVGGNETNENKNKTFESEDLKNEKSFKPIIIGKYNLIYIFYSIEFICLLLFICLHVLLFL
HsppP5A VNEKTGIESVNEKTGIESVNEKTGIESVKITASNNSSRSNSNV------------------------NGSTSNNSNNNKNEEDYSHKPIIIGKYNLIYIFYSIEFVCLLLFILLHLLTFF
PberP5A ---------------------GNKLNKLKIKKGKTNNKG-----------------------------------------NSDNTFKPIIIGKYNLIYIFYSIEFVCLLIFIAFHLLTFL
PyoeP5A ---------------------GNKLNKLKIKKEKEKEKEKDKE---------------------------KENEKDKENTNSDNTFKPIIVGKYNLIYIFYSIEFVCLLIFIAFHLLTFL
PchaP5A ---------------------GNKLSTLKTKKEKKDNKE-----------------------------------------NNDNTFKPIIVGKYNLIYIFYSIEFVCLLIFIVFHLLTFL
PvinP5A ---------------------GNKLSALKTKKGKKDNKE-----------------------------------------NVDNTFKPIIIGKYNLIYIFYSIEFVCLLIFIVFHILTFL
PgonP5A ----------DLGYINVETKERKDKNEWLNKKTTIVEENEKN-------------------------RNSKEEEKESKKKGKENSFKPIIVGKYNIIYIFYSIEFFSLLLFILFHLLTFL
PvivP5A ----------PMGEFKMGPGEGKDSGEFTNGKGSENGKGSAN-----------------------------GEQTGSNGKSKENAFKPIIVGKYNLIYLFYSLEFVGLLLFILFHLLTFL
PcoaP5A ---------------------KDSSQFTSGKHSEQGD------------------------------------EKGSNREGKENTFKPIIIGKYNLIYLFYSIEFVSLLLFIFFHLLTFL
PcynP5A ---------------------KDSSESINGRGNANGEHSANEE------------------------HSSKGEQKGSNRKGKENTFKPIIIGKYNMIYLFYSSEFVCLLLFIFVHLLTFL
PfraP5A ---------------------KDSSEFINWKGSANGE------------------------------------QKDSNKKEKENTFKPIIIGKYNLIYVFYSIEFVGLLLFILFHLLTFL
PinuP5A ---------------------KDSNEFINAKENANGEDSAN------------------------------GEQKGSNRKEKENTFKPIIIGKYNLIYLFYSSEFVGLLLFIFFHLLTFL
PknoP5A ---------------------KDSREFINRKGSANGE------------------------------------EEGSNREGKENTFKPIIIGKYNLIYLFYSIEFVGLLLFIFFHVLTFL
PbraP5A ---------------------KDSVEFPNNEKGSSKEKADYN-------------------------NSNNGKEKEFNNKNRDNSFKPIIIGKYNLIYIFYSVEFVSFLLFMFFHLLIFL
PmalP5A ---------------------KDSVEFPNNEKGSSKEKADYN-------------------------NSNNGKEKEFNNKNRDNSFKPIIIGKYNLIYIFYSVEFVSFLLFMFFHLLIFL
PovCP5A ---------------------KAKENYDRASSEKSNSQNGGD-----------------------------AEGERRKVDERDNSLKPIIVGKYNLIYIFYSIEFVIFLFFVFFHVLTFL
PovWP5A ---------------------KAEENHDRTSSEKSNSQNGGD-----------------------------AKGERRKVDERENSLKPIIVGKYNLIYIFYSIEFVSFLFFVFFHLLTFL
```

```
           <-|->  N-terminal domain (NTD)                        VR2
HtarP5A   LSQWNLNAYLLVAFKNVDSKDKRIYLYNIRNRCTHVYIKPLEG-----RVPLENSNSNNNNGKSTKCSV---YNSKERYNTDSSFYVPKAELVELIKENHDIYFFFKQKKYLFNYKTLQF
PrelP5A   LSQWNLNFYLFIAFNNLNCNDKNKYIYNLRNLCTHVYIKP-----------LLKKN--DKAG----------INSKERFNTDYNFYKPKADLVELKKINNDIFFFYKQKKYLFDYKTVKF
PgalP5A   LSQWNLNVYLFIAFKNLNSNDKNKYIYNLEKLCTHVYIKP-----------LMKTD--EKIG----------INSKERFNTDYNFYKPKAELVELKKLNNDIFFFYKQKKYLFDYKLVKF
PfalP5A   LSQWNIKINLFISYKSLYSKNKEKYLYNLKNFCTHVYIEP----------CIIKSE--EDNG----------IYDKNRYNMDCNFYKPKSELIELKKIDNDIYFFYKQKKYIFNYETFIF
PpraP5A   LSQWNIKINLFISYKSLYSKNKEKYLYNLKNFCTHVYIEP----------CIIKNE--EDNG----------IYDKNRYNMDCNFYKPKSELIELKKVDNDIYFFYKQKKYIFNYETFIF
PreiP5A   LSQWNIKINLFISYKSLYSKNKEKYLYNLKNFCTHVYIEP----------CIIKSE--EGIG----------IYDKNRYNMDCNFYKPKSELIELKKVDNDIYFFYKQKKYIFNYKTFIF
PadlP5A   LSQWNIKINLLISYKSLHNKDKKKYLYNLKNLCTHVYIEP----------CIIKSE--EGNSGS--------IYDRNRYNMDSNFYKPKAQLIELKKVDNDIYFFYKQKKYIFNYNTFVF
PbilP5A   LSQWNVKINLFISYKSLYSKDKEKYLYNLKNICTHVYIEP----------YIIKTE--QGSG----------IYDRNRYNMDSNFYKPKAELIELKKVHNDIYFFYKQKKYIFNYNTFIF
PblaP5A   LSQWNIKVNLFISYKSLYNKDKEKYIYNLKNLCTHVYIEP----------YIKQTE--EGSG----------IYDRNRYNMDSNFYKPKAQLIELKKVDNDIYFFYKQKKYIFNYNTFIF
PgabP5A   LSQWNIKMNLLISYKSLYSKDKKKYLYNLKNLCTHVYIEP----------CIIKSE--ECNSSS--------IYDRNRYNMDSNFYKPKAQLIELKKVDNDIYFFYKQKKYIFNYNTFVF
HsppP5A   LSQWSLMVNLFISFNRLNNHDKKAYIYNLQNVCTHVYIKPIILKMKGQSKSVGDRSSNPSDGKNELLRNSIEYNRKDRFNTDYNLYKPKSELVELKKEGYDIFFFYKQKKYIFNYEILEF
PberP5A   LSQWNLSINLFIAYIRLSNKERDKYIYNLQKFCTHIYIKP----IKSERFQKINKQKGLSKCVKNCLPNKNDTKQKERFNTDYNLYKPKSILVELKKENNDIYFFYKHKKYIFNYETLNF
PyoeP5A   LSQWSLSINLFIAYSRLSNKGRDKYIYNLQKFCTHIYIKP----IKSEGFQKINKEKGLSKCVKNCLPNKNDTKKKERFNTDYNLYKPKSILVELKKENNDIYFFYKHKKYIFNYETLNF
PchaP5A   LSQWNLSINLFIAYNKLSNKGRDRYIYNLQKFCTHIYIKP----IQTGSFQNINKQKGLSKCVKNCWPSKNDAKKKERFNTDYNLYKPKSMLVELKKENNDIYFFYKHKKYIFNYETLNF
PvinP5A   LSQWNLSINLFIAYNKLSNKGRDRYIYNLQKFCTHIYIKP----IQGESFQKINKQKGLSKCVKSCWPSKNDIKKKERFNTDYNLYKPKSMLVELKKENNDIYFFYKHKKYIFNYETLNF
PgonP5A   LSQWNLNVNLFVSFNRLNNNDKNKYVYNLQNLCTHVYIKP-----------LIRRS------------DKSGINSKERFNTDYNLYKPKSELVPLKKGGNYIYFFYKQKKYIFNYETLDF
PvivP5A   LSQWNLRVNLVVSFSRLNSSDRDKYLYNVQKKCTHVYIKP-----------LVKRS------------DKSAVNSKERFNTDYNLYKPKAELVPLKRGKNYIFFFYKQKKYIFNYETLDF
PcoaP5A   LSQWNLSVNLFVSFSRLSSSNKDKYLYNVQNKCTHVYIKP-----------RVKRS------------DKLSINSKERFNTDYNLYKPKAELVPLKRGKNYIYFFYKHKKYIFNYETLDF
PcynP5A   LSQWNLSVNLVVSFSRLNSSDKDKYLYNVQYQCTHVYIKP-----------LVKRS------------DKSAVNSKERFNTDYNLYKPKSELVPLKRGKNYIFFFYKQKKYIFNYETLDF
PfraP5A   LSQWNLSVNLVVSFSRLNSSDKDKYLYNVQNKCTHVYIKP-----------LVKRN------------DKSAVNSKERFNTDYNLYKPKSELVPLKRGKNYTFFFYKQKKYIFNYETLDF
PinuP5A   LSQWNLDVNLVVSFSRLNSSHKDKYLYNVQNRCTHVYIKP-----------LVKRS------------DKSAVNSKERFNTDYNLYKPKAELVPLKRGKNSTFFFYKQKKYIFNYETLDF
PknoP5A   LSQWNLSVNLFISFSRLNSSDKDKYLYNVQNKCTHVYIKP-----------LVKRS------------DKLSVKSKERFNTDYNLYKPKSELVPLKRGKNYIFFFYKHKKYIFNYETLDF
PbraP5A   ISQWNLNVNLLVCYIHLNNTNQNKYIYNLKNLCTHVCIKP-----------LTKKN------------DKSGIHSKERFNTDYNLYKPKSELVELKKGKNDIFIFYKQKKYIFNYKTLDF
PmalP5A   ISQWNLNVNLLVCYIHLNNTNQNKYIYNLKNLCTHVCIKP-----------LTKKN------------DKSGIHSKERFNTDYNLYKPKSELVELKKGKNDIFIFYKQKKYIFNYKTLDF
PovCP5A   LSQWNLYINLIVSYNRLNSSDRNSYIYNIQHLCTHIYIKP-----------LIRRS------------DNSGISNKDRFNMDYNLYKPKAELVELKKENNNLFFFYKQKKYIFNYETLHF
PovWP5A   LSQWNLCINLIVSYNRLNSSDRNSYIYNMQHLCTHVYIKP-----------LIRRS------------DNSGISNKDRFNMDYNLYKPKAELVELKKENNNLFFFYKQKKYIFNYETLHF
```

```
       <-|->  A-domain                                                VR3
HtarP5A DSLTHFDNFPISFYVKWKGLIETDTEG--IFKENEANEFCDEIRKEVKQGK----------------------MNTVLKKISLLYSERWGQLPKNSKKGSALEKASKTT-----------
PrelP5A ESLKHFDIFNLSFYLKWKGLIEYEKE---AYDNIQVNNISNDIKRSIKNED----------------------LNSLFNKIDILYSDKLKKKDIKSYKDKTKVIDSDNN-----------
PgalP5A ESLKHFDIFNVSFYLKWKGLIECEQN---LYDDLYISNITEDIKRSIKSEN----------------------LNDLFNKIDILYSDKARRKDIKSNKTKTKLVDSDNI-----------
PfalP5A ESLKHFDNFNLSFYLNWKGLLDYPKGKDINKMDIYNNKMMRDIKDRVTCNN----------------------IESVIKKLDILYALHNDKKGNLNIDEKVDDVNFNCN----------D
PpraP5A ESLKHFDNFNLSFYLNWKGLLDYPKGKDINKMDIYNNKMMRDIKDRATCNN----------------------IESVIKKLDILYALHSDKKGNLNIDEKVDDVNFNCN----------D
PreiP5A ESLKHFDTFNLSFYMNWKGLLDYPKGEDINKMDMYNNRMMRDINESVTCNN----------------------IESVIKKLDILYTLHSDKKGNLNIDEKVDDVNFNRN----------D
PadlP5A ESLKHFDNFNVGFYLNWKGLIDYNKGSVVNKMDIYNNKMMKNIKESIACNN----------------------IESIIKKLDTLYTSHSDTSKKVYMNEKVDEMNDNNNNNNNNNNNNNN
PbilP5A ESLKHFDNFNLSFYMNWKGLLYYNKERDINKLDMYNNKMMKDINESVRCNN----------------------IESVTKKLDILYISHIDNRENLDMNKKVDYVNKNGN----------D
PblaP5A ESLKHFDNFNLSFYMNWKGLLDYNKGKDVNKMYIYNNKMMRDINESVTCNN----------------------IESVIKKLDILYTSHNDIKGNLDLNEKVDDVNNICN----------D
PgabP5A ESLKHFDNFNVGFYLNWKGLIDYNKGSGVNKMDIYNNKMMKNINESVTCNN----------------------IESVIKKLDILYTSHSDNSKKVYMNEKVDEVNDNNNNNNNN----ND
HsppP5A QSIKQFDVFNINFYLKWKGLIDEESS---NISNNSNRKYINNICKNIEDCLSY---------------EKYKNLNKLQEDIDKLYVHSSNKGMGKSSDKHVGDQKRYDT-----------
PberP5A ESVKHFDIFNLPFYLNWKGLIECEHK---IPCQENLVKKIHKESE----------------------------LLYIDSNAESSYINGWKNLINNIKELYEGICFKKN------------
PyoeP5A ESVKHFDIFNLPFYLNWKGLIECENK---ISCQENFVKKINKESE----------------------------ILYIDGNIESSYINEWKNLINNIKELYEIECFKKN------------
PchaP5A ESVKHFDIFNIPFYLNWKGLIECEHK---VPCQENFIKKIHTEAK----------------------------LFYTNENIESNYINTWNGVISSIEELYEAECFKKNT-----------
PvinP5A ESVKHFDIFNLPFYLNWKGLIESEYK---VPCQENLVKKIHKEAK----------------------------LFYNNEHIESNYINTWNEVISSIEELYESECFKKNT-----------
PgonP5A ESVKHFDTFNLSFYQKWKGLIECKQE--NLQDDVNMCKTLREIEENVKNHQ-----------------RRSGNLNILFLQLDGLYLSKGIISEMS-KLVHNNNKGAGNK-----------
PvivP5A ESVKHFDNFNLSFYQKWKGLIECPMGG-DLYEDEDTSTTLQRIEENVKKIQ-----------------KRGGNVHILFCQLDGLYLDKGGGSGMA-QLAYRNKKGGNNP-----------
PcoaP5A ESVKHFDNFNLSFYQKWKGLIEYQMG--GLYDDGNTSTTLQGIEENVKKIE-----------------KRGGNVNTLFCQLEGLYLDKGGRSGID-QLAHCNK-GGNNI-----------
PcynP5A ESVKHFDNFNLSFYQKWKGLIECQMG--GLYDDENTSTTLQGIEENVKSIQ-----------------KRGGNVHILFCQLDGLYLDNGGKSGMT-QLSQRNK-GGNNP-----------
PfraP5A ESVKHFDNFNLSFYQKWKGLIEYQMG--DLYHDENTRITLRGIEENVKNIQ-----------------KRRGNVSILFCQLDGLYLDKGGLSGMA-QLARRNK-GENNI-----------
PinuP5A ESVKHFDNFHLSFYQKWKGLIECQMR--DLYDDEKTGTTLRAMEQNVKNIQ-----------------KRGGNVHILFCQLDGLYLDKAGRCAMA-QLAQRYK-GGSNS-----------
PknoP5A ESVKHFDNFNLCFYQKWKGLIEYQMG--SLYDDENTSATLQRIEENVKNIE-----------------KRGINVNTLFCQLEGLYLDKSGRSGIN-QLAHRNK-GGNRL-----------
PbraP5A ESVKHFDIFNLSFYLKWNGLIEFETD-DIYDEINMSHTLRDIEESVNYYEDYYYHNDCDDRNDGDNGNDDTNNLNALFSKIDALYLGK--RNDIA-KSANKSNKMYNEL-----------
PmalP5A ESVKHFDIFNLSFYLKWNGLIEFETD-DIYDEINMSHTLRDIEESVNYYEDYYYHNDCDDRNDGDNGNDDTNNLNALFSKIDALYLGK--RNDIA-KSANKSNKMYNEL-----------
PovCP5A EAVKHFDTFNISFYVKWKGLIECLPSDTVCGSPFVNSLIPQIEKCLKEES--------------------GYSLKDFLTILDELYGEYTEKAQDR-EGMRSTKMESIDK-----------
PovWP5A EAVKHFDTFNISFYVKWKGLIECQPSDSVCDSPFVNSLIPQIEKCLKEES--------------------GYSLKHFLTILDELYGEYTEKVQGR-EGMRSTKMESIDK-----------
```

```
                                                                         VR3
HtarP5A --------------------------------KEKYHHRKEKVEIQNGE----------------------------------------------------VQKEPIEMSELNNLPCMN-
PrelP5A --VKEE---------------------------GRKINRLMNDKNKDEET-------NFSEV---------------------------------------NKNSKVHVIDNHKIIN---
PgalP5A --LKEE---------------------------ENKRSNLINDKNKNEEN-------NSNEV---------------------------------------NKSKKVHIMESQGKMN---
PfalP5A FISKEKN------VDNVYDMNKKNMINIKYRGKTNKGNNNFIDVLKNDN------MCEKDINKNDIFGDYNKDDMDDT---HNHNVDRNIIC---------SNKKKVD-SIKNSIHNDVV
PpraP5A FIGKEKN------VDNVYDMNKKNMINIKYRGKTNKGNNNFIDVLKNDN------MCEKDINKNDIFGDYDKDDMDDT---HNHNVDRNIIC---------SNKKKVD-SIKNSIHNDVV
PreiP5A FIAKEKN------VHNVYDMNKKYMINIKYRGKTNKGNNNFIDLLKNDN------MCEKDINKNDIFGDYDKDIMDDGGDLHDNNVDRNVIYRNKERLDKMSNKKKVH-TIENSIDNDVR
PadlP5A LVHKEKNVNNIYDMKNIDDTNKNNMN-IKYRGKTNKGNNNHIDISKSYNMN---NMCEMDDG---------------------------------------DNKKKIY-NIQNNIHNNIK
PbilP5A FIDKEK---------NIDNTN--DMINIKYRSKTNKGNNNFVDISRRSNKS---NMCEKDEK----------DEKDDN------NVDRNNIYSNKERFDKMSSKKKIY-TIENSIQYNDK
PblaP5A FIDKEKNVVNAYDMRNIHNINK-NMRNIKYRGNTNKVNNNFIDISRNSNIS---KICEKDINKNDRFELYEINEKDDN------NVNRNNIHRNKDTLDKMSNKKKIYNTIENSIHNDVR
PgabP5A LINKEKNVNNIYDMKNMDDTNKSNMN-IKYRGKTNKGNNNHIDKSKGDNMNNMNNVCERDDG---------------------------------------DNKKKIY-NIQNNIHNNIK
HsppP5A --TKMEVQRKDYDTQVGMRERKTQVERHSVMSDEVIMSKNINNTIIDKEKEVISKDNNNKKIHISDNILVN--EAKTNILYFLKHN----------------------------------
PberP5A --TRNKCNSIG---------------------NNGELNEHVNYQLKDGYE---ISGKINKKIGITNISN------INNNFD---------------------------------------
PyoeP5A --TKSKCNIIE---------------------NNEELNENINYQVKGGYE---ISGKNNKKIGITNIYKNVYKNDYNNDYNNDYNNDYKNVYKSVY------------------------
PchaP5A --ARDKCISIGNTGDNNN-------------YNNGISSEPINGQRKGSDD---ILDQIGKKIGIADINN------INKKFY---------------------------------------
PvinP5A --ARSKSTSISNTGDSN---------------NNGELSKPINDQLKGDDD---ILDQLSKKIGIADIN---------KKIY---------------------------------------
PgonP5A --PEEEIENIYEYTKK-------------GNEKMPKLLQTIEE--RNMSQL----SYHNGVL---------------------------------------KKRNKVHIDESKNMQHEKD
PvivP5A --SEEEIEDVYHYTRGGR-----------GNSKGAKILHSIEG--STTMQR----SICDEEL---------------------------------------KSKKRVHPTEGEGKQNGNS
PcoaP5A --SEEEIEDVSHYTRKGK-----------GSAEG-----------STTVQG----SVKRDEV---------------------------------------KSKNRVHPTQGGRKQNGHQ
PcynP5A --SEKEIENIYDYTRKGR-----------EREKGTKILHFIEG--STTIER----SIQGDEL---------------------------------------KRKNRVHPTQGKSKQNGDK
PfraP5A --YGEEIEDVYQYNRKGR-----------GCAEGTKVLHSIEG--STTIQS----SIEGDEL---------------------------------------KVKNRVHPTQGKSKQYGNQ
PinuP5A --SEEEIEDVYHYSMKGR-----------GSSKGTKMLQSFEG--STTIQR----SIDADEL---------------------------------------KNRNRVHPSQGESKQNGNQ
PknoP5A --SEEEIEDAYDYIGKER-----------GSEEGTKIGHSIEG--SSTIGR----SIQGDER---------------------------------------KSKKRVHPIEGGRKQNVDQ
PbraP5A --GQEEGGNIHSTVRGRG-----------KKSKQIHTNETLNK--SHESN-----FPHGDIN---------------------------------------KNKKKVEISEIKN--DGSN
PmalP5A --GQEEGGNIHSTVRGRG-----------KKSKQIHTNETLNK--SHESN-----FPHGDIN---------------------------------------KNKKKVEISEIKN--DGSN
PovCP5A --EEQ-----WKE----------------GEGDGTRKRRTQGGDLHSIDGA----ESKGKNY---------------------------------------ERKKKVHLSSTKDAYRSDN
PovWP5A --EEQRKEEQWKEEQRKE-----------GEGDGTRKRRTQGGDLHSIDEA----ESKGKNY---------------------------------------ERKKKVHLSNAKVANRSDN
```

```
            VR3  <-|->           A-domain           <-|->       cTM1           <-|   |->    cTM2      <-|->       A-domain
HtarP5A ----------LFYNKRNYFELPYDVYVDRNLNKYGRNVYNIPAPCFKTLLYEALLSPFFIFQFFSIILWMLDSYWHFGVFSIFILIVLEAQLISKRIRDFNLINDMKVPAHNVYVYRNLK
PrelP5A ---------ENFYYNKNYFQIPHDLYVNNNLNKYGENIYDIPSPCFKKLLYESMLSPFFIFQFFSIILWMLDSYWYFGVFSIFILVVLEAQLINKRIREFNLINSMKVPSQNVYVYRNMQ
PgalP5A ---------ENFYYNKSYFQIPHDLYVNNNLNKYGENIYDIPSPCFKKLLYEAMLSPFFIFQFFSIILWMLDSYWYFGIFSIFILIVLEAQLINKRIREFNLINSMKVPAQNVYVYRNMQ
PfalP5A RDDNVNNNFYNFYYKKSCFEIPYDIFVHNNVEKYGENIYDIPCPCFKELLYESMLSPFFIFQFFSIVLWMLDSYWYFGIFSIFILIILESQLINKRIREFNMINGMKVDPQDVYVYRNLR
PpraP5A RDDNVNNNFYNFYYKKSCFEIPYDIFVHNNVEKYGENIYDIPCPCFKELLYESMLSPFFIFQFFSIVLWMLDSYWYFGIFSIFILIILESQLINKRIREFNMINGMKVDPQDVYVYRNLR
PreiP5A RNDHVNNNSYNFYYKKSSFEIPYDIFVHNNVEKYGENIYDIPCPCFKELLYESMLSPFFIFQFFSIVLWMLDSYWYFGIFSIFILIILESQLINKRIREFNMINGMKVDPQDVYVYRNLR
PadlP5A KNDNLN-DSNSFYYKKSSFEIPYDIFVHNNIEKYGENIYDIPCPCFKELLYESMLSPFFIFQFFSIVLWMLDSYWYFGIFSIFILIILESQLINKRIREFNMINGMKVDPQDVYVYRNLR
PbilP5A RNDYFN-NCYSFYYKKSSFEIPYDIFVHNNIEKYGENIYDIPCPCFKELLYESMLSPFFIFQFFSIVLWMLDSYWYFGIFSIFILIILESQLINKRIREFNLINGMKVDPQDVYVYRNLR
PblaP5A KNNYFN-DSYYFYYKKSSFEIPYDIFVHNNVEKYGENIYDIPCPCFKELLYESMLSPFFIFQFFSIVLWMLDSYWYFGIFSIFILIILESQLINKRIREFNMINGMKVDPQDVYVYRNLR
PgabP5A KNDNLN-DSNYFYYKKSSFEIPYDIFVHNNIEKYGENIYDIPCPCFKELLYESMLSPFFIFQFFSIILWMLDSYWYFGIFSIFILIILESQLINKRIREFNMINGMKVDPQDVYVYRNLR
HsppP5A ---------PEYYLKKCFYHIPYDIYVNNNLKKYGENTYDIPSPNFKKLLYEAMLSPFFIFQCFSILLWMLDSYWIFGLFSVFILITLEAQLIHKRIREFNMINSMKMPAQDVYVYRSLK
PberP5A ----------SYHCKKNYFEIPYDLYVHNNLSKYGENIYDIPSPCFKKLLYEAMLSPFFIFQFFSIILWMLDSYWYFGIFSIFILIVLESQLINKRIREFNLINSMKMSPQNVYVYRNLQ
PyoeP5A ----------NYDCKKNYFEIPYDLYVHNNLNKYGENIYDIPSPCFKNLLYQAMLSPFFIFQFFSIILWMLDSYWYFGIFSIFILIVLESQLINKRIREFSLINSMKMSPQNVYVYRNLQ
PchaP5A ----------GYDCKKSYFEIPYDLYVHNNLSKYGENIYDIPSPCFKKLLYEAMLSPFFIFQFFSIILWMLDSYWYFGIFSIFILIVLESQLINKRIREFNLINSMKMCPQNVYVYRNLQ
PvinP5A ----------GYNCKKSYFEIPYDLYVHNNLSKYGENIYDIPSPCFKKLLYEAMLSPFFIFQFFSIILWMLDSYWYFGIFSIFILIVLESQLINKRIREFNLINSMKMCPQNVYVYRNLQ
PgonP5A TDSF-----FNYYKKKSSFEIPHDIFVHNNIDKYGENIYDIPSPCFKKLLYEAMLSPFFIFQFFSILLWMLDSYWYFGIFSIFILVVLEAQLINKRIREFNLINSMKVPAQNLYVYRNLQ
PvivP5A ADPF-----LSYYKK-SNFEIPYDIFVHNNLDKYGENTYDIPSPCFKKLLYEAMLSPFFIFQFFSILLWMLDSYWYFGIFSIFILVMLEGQLINKRIREFNLINSMKVPAQNLYVYRNLQ
PcoaP5A TDPF-----LSYYKK-SNFEIPYDIFVHNNLDKYGENIYDIPSPCFKKLLYEAMLSPFFIFQFFSILLWMLDSYWYFGIFSIFILVMLESQLINKRIREFNLINSMKVPAQNVYVYRNLQ
PcynP5A TDPF-----LSYYKK-SNFEIPYDIFVHNNLDKYGENIYDIPSPCFKKLLYEAMLSPFFIFQFFSILLWMLDSYWYFGIFSIFILVMLEGQLINKRIREFNLINSMKVPAQNLYVYRNLQ
PfraP5A RDSF-----LSYYKK-SNFEIPYDIFVHNNLDKYGENIYDIPSPCFKKLLYEAMLSPFFIFQFFSILLWMLDSYWYFGIFSIFILVMLEGQLINKRIREFNLINSMKVPAQNIYVYRNLQ
PinuP5A TNPF-----HSYYKK-SNFEIPYDIFVHNNLDKYGENIYDIPSPCFKKLLYEAMLSPFFIFQFFSILLWMLDSYWYFGLFSIFILVMLEGQLINKRIREFNLINSMKVPPQNLYVYRNLQ
PknoP5A TDPF-----LSYYKK-SNFEIPYDIFVHNNLDKYGENIYDIPSPCFKKLLYEAMLSPFFIFQFFSILLWMLDSYWYFGIFSIFILVMLEGQLINKRIREFNLINSMKVPAQNVYVYRNLQ
PbraP5A NDPF-----FYDYKKKNYFEIPYDIYVHNNLSKYGENIYDIPSPCFKKLLYEAMLSPFFVFQFFSIILWMLDSYWYFGIFSIFILIVLEAQLINKRIREFNLINSMKVPAQNVYVYRNLQ
PmalP5A NDPF-----FYDYKKKNYFEIPYDIYVHNNLSKYGENIYDIPSPCFKKLLYEAMLSPFFVFQFFSIILWMLDSYWYFGIFSIFILIVLEAQLINKRIREFNLINSMKVPAQNVYVYRNLQ
PovCP5A ---------LYYYKR-SHFEIPYDIFVHNNVNKYGENIYDIPSPCFNKLLYESLLSPFFIFQFFSILLWILDSYWYFGLFSIFILILLEAQLINKRIREFNLINSMKVPAQNVYVYRNLE
PovWP5A ---------PYYYKK-SHFEIPYDIFVHNNVNKYGENIYDIPSPCFNKLLYESLLSPFFIFQFFSILLWILDSYWYFGLFSIFILILLEAQLINKRIREFNLINSMKVPAQNVYVYRNWE
```

```
                                                A-domain                                             VR4
HtarP5A WKIILSSSLLPGDIYILETSMSG-----GADICTCETLLVDGMCITDESILTGESVPLIKAAIDKEEGENED------------VNDEEENNDGKASTSTSS------------------
PrelP5A WKIIKSNMLLPGDIYILSNELNG-----NDNICTCETLLIEGICITDESILTGESVPLIKAAIDKNTEEKNVESSYEN------------------------------------------
PgalP5A WKIIKSNMLLPGDIYILANELNG-----NDNICTCETLLIEGICITDESILTGESIPLIKAAIDKN----------------------TEEKN-DINFEK--------------------
PfalP5A WTIMKSNKLLPGDIYILTNDMTAT----DNNICTCETLLIDGTCITDESILTGESVPLIKACIDKSVINNKSNNNNNKNENNNNKNENNNKKKDNNKNENNNKKKDNNKNENNNKKKDNN
PpraP5A WTIMKSNKLLPGDIYILTNDMTAT----DNNICTCETLLIDGTCITDESILTGESVPLIKACIDKSVINNKNENNNNNKNENNN----KKKDNNKNENNNKKKDNN--------------
PreiP5A WNIMKSNKLLPGDIYILTNDMTAT----DNNICTCETLLIDGTCITDESILTGESVPLIKSCIDKSVINKHNNNNNNNSYNNNSYNNNNSYNNNGYNNNGCNNNSY--------------
PadlP5A WNIMKSNMLLPGDIYILTNDMSMT----DNNICTCETLLIDGTCITDESILTGESVPLIKACIDKSIMISDNKMIKSEKNEKNEKNEKIEKNEKNEKNEKNEKNEKIEKNEKNEKNEKNE
PbilP5A WNIIKSNMLLPGDIYILTNDMTMT----DNNICTCETLLIDGTCITDESILTGESVPLIKSCIDKSVINSKNNNNNK-------------------------------------------
PblaP5A WNIMKSNKLLPGDIYILTNDMTMT----DNNICTCETLLIDGTCITDESILTGETVPLIKTCIDKSVIKKSNNNNNIKNNDNN--NNNIKNNDNNNNNNNNNNNNI--------------
PgabP5A WNIMKSNMLLPGDIYILTNDMSMT----DNNICTCETLLIDGTCITDESILTGESVPLIKACIDKSIIGSDNKMIKNEKNEKNEKYEKYEKNEKYEKYEKYEKNE---------------
HsppP5A WRIIKSDKLLPGDVYILSFEHNNKNNSNNDYICTCETLLVEGVCITDESILTGESVPLIKAAVDKSGLNDGNLGDSNGDKTVQHYAKSNKNNENDGNKGNNENDGNKGNNENDGNNGNNG
PberP5A WKIIKSNYLLPGDIYILTNDING-----NDNICTCETLLLEGMCITDESILTGESIPLIKASIDK-------------------------------------------------------
PyoeP5A WNIIKSNYLLPGDIYILSNDING-----NDNICTCETLLLEGMCITDESILTGESIPLIKASIDK-------------------------------------------------------
PchaP5A WKIIKSNLLLPGDIYILSNDING-----NDNICTCETLLLEGMCITDESILTGESIPLIKASIDK-------------------------------------------------------
PvinP5A WKIIKSNLLLPGDIYILTNDING-----NDNICTCETLLLEGMCITDESILTGESIPLIKASIDK-------------------------------------------------------
PgonP5A WKIIKSSMLLPGDIYILSNETNG-----GDNLCTCETLLLDGVCITDESILTGESIPLIKAAIDKT-EEDFM------------------------------------------------
PvivP5A WKVIKSNMLLPGDIYILSNETSG-----GDNVCTCETLLLEGVCITDESILTGESIPLIKAAIDKAEEEEYA------------------------------------------------
PcoaP5A WKVIKSNMLLPGDIYILSNETSG-----GDNVCTCETLLLEGVCITDESILTGESIPLIKAAIDKTEGEEYL------------------------------------------------
PcynP5A WKIIKSNMLLPGDIYILSNETSG-----GDNVCTCETLLLEGVCITDESILTGESIPLIKAAIDKTEEEEYV------------------------------------------------
PfraP5A WKIIKSSMLLPGDIYILSNESSG-----GDNVCTCETLLLEGVCITDESILTGESIPLIKAAIDKTEEEEYV------------------------------------------------
PinuP5A WKIIKSNMLLPGDIYILSNETSS-----GDNVCTCETLLLEGVCITDESILTGESIPLIKAAIDKTEEEEYL------------------------------------------------
PknoP5A WKIIKSNMLLPGDIYILSNETNS-----VDNVCTCETLLLEGVCITDESILTGESIPLIKASIDKTEGEEYI------------------------------------------------
PbraP5A WKIIKSNMLLPGDIYILSNEIRG-----SDNICTCETLLLEGVCITDESILTGESVPLIKAAIDKNVEEE--------------------------------------------------
PmalP5A WKIIKSNMLLPGDIYILSNEIRG-----SDNICTCETLLLEGVCITDESILTGESVPLIKAAIDKNVEEE--------------------------------------------------
PovCP5A WKTIKSNMLLPGDIYILSNETSA-----NDHVCTCETLLLEGICITDESILTGESVPLIKAAIDRNVENG--------------------------------------------------
PovWP5A WKSIKSNMLLPGDIYILSNETSA-----NDLVCTCETLLLEGICITDESILTGESVPLIKAAIDRNVENG--------------------------------------------------
```

```
                                                 VR4                                                  A-domain
HtarP5A ---------------------------------------------------------------KVHEIENTDHEEVFVS------KRIATLVSKEKLDIKNKHKKHIVYAGTKLLLTKNE
PrelP5A ------------------------------------------------------------------------------------------SIFLDRIDIKNKHKKNIIYAGTNILLTKNE
PgalP5A ------------------------------------------------------------------------------------------SIFLDKIDIKNKHKKNIVYAGTNVLLTKND
PfalP5A ------------------------KNNDNN-----------KNNSNNNSYR--FIGDDNVERLNYKNFENNENEFIKDKECDYESSNYCNSLFCNRLDIKNKHKKHIVYAGTNILMTKNE
PpraP5A ---------KNNDNN--------------------------KNNNNNNSYR--FIGDDNVERLNCKNFENNENEFIKEKECDYESSNFCNSLFCNRLDIKNKHKKHIVYAGTNILMTKNE
PreiP5A ------------------------NNNAYN-----------NNSYSNNSYSNTFIGDDNVERLNYKNCENNKNKFIKKKECDYESSNFCNSLFSNRLDIKNKHKKHIVYAGTNILMTKNE
PadlP5A KNEKIEKNEKNEKNEKNEKIEKNEKNDDTNHMNHMNHMNHMNHMNHINSYNSTCNLQSDGENLHHTYYQNKENEFIKNDEYHFDHCNVSNSLFCNRLDIKNKHKKHIVYAGTNILMTKNE
PbilP5A ----------NNGNS--------------------------KNNNSNNTFS----IENNIESLNNTDYRNNKNKYIKKGEYDDEATNFSNSLFCNRLDIKNKHKKHIVYAGTNILMTKNE
PblaP5A ---------NNNNNN--------------------------NNNNNNNTFN----IEDNMNSLNNTDYLNNKNNYIKKGEYDYEATNFSNSLYCNRLDIKNKHKKHIVYAGTNILMTKNE
PgabP5A ---------KNEKND------------------------HMNHINNINSYNSTCNLQSDGETLHDTYYQNNPNEFIKNDEYYFDDWNVSNSLFCNRLDIKNKHKKHIVYAGTNILMTKNE
HsppP5A NNGNNGNNENNGNNGNNGNSENGENCENNENNENNENNE------------NNE-NNGNNENNENNENNENNGNNENNGNNETSHETNNTLLSSNMIDIKNKHKKHVVYAGSKILLTKNE
PberP5A -----------------------------------CVDMHNNDNCNDNKNID--------------EIYYSS--------------------YFEKIDIKNKHKKHVVYAGSNILLTKNE
PyoeP5A -----------------------------------GVAINSNDNDNDNKNLD--------------GTSYYS--------------------YFDKIDIKNKHKKHVVYAGSNILLTKNE
PchaP5A -----------------------------------GVDMHSSDNFIDNKNRE--------------EIDYSS--------------------YLNKIDIKNKHKKHVVYAGSNILLTKNE
PvinP5A -----------------------------------GVDMHNGNNLIDTQNQD--------------EIDYSS--------------------YLNKIDIKNKHKKHVVYAGSNILLTKNE
PgonP5A ------------------------------------DDYDSINNNFDEMEE----------------PSENQ--------------MDGISIFANKIDIKNKHKKHVVYAGSNILLTKNE
PvivP5A ------------------------------------EQGDSDHDTCGDSGG----------EVKGDFQSDEAPSVVG---------TKGSSIFANKIDIKNKHKKHVVYAGSNILLTKNE
PcoaP5A -----------------------------------DHDTSDNDTCADWED-------------GGDPTMGISP-VGG---------TKGSSIFTNKIDIKNKHKKHVVYAGSKILLTKNE
PcynP5A -----------------------------------EQDVSDNDTCGDWGG-------------EGDSMGGVSP-IEG---------TKGSSIFTNKIDIKNKHKKHVVYAGSNILLTKNE
PfraP5A -----------------------------------EQDASDNDTCADWGG-------------EGDAMSGVSP-MEG---------IKGRSIFTNKIDIKNKHKKHVVYAGSKILLTKNE
PinuP5A -----------------------------------EQDGSDNDTCGDWGGAAEEEAEAEDGEEEGDPMRGVSP-MEGNKGNKRNKRNKGNSIFTNKIDIKNKHKKHVVYAGSNILLTKNE
PknoP5A -----------------------------------QEDTSDNDTCCDWA---------------GHSMEGVSP-MGG---------SKGNSIFTNKIDIKNKHKKHVVYAGSKILLTKNE
PbraP5A -----------------------------------------NDVCIKKGG-----------------EKNGNR----------------ISIFSNKIDIKNKHKKHIVYAGSNILLTKNE
PmalP5A -----------------------------------------NDVCIKKGG-----------------EKNGNR----------------ISIFSNKIDIKNKHKKHIVYAGSNILLTKNE
PovCP5A -----------------------------------------NDSSITKDG----------------KMSEEE--------------LDASSIYVDKINIKNVHKKHIVYAGSNVLLTKNE
PovWP5A -----------------------------------------DDSSTTKGE----------------KMSEEE--------------LEASSIYVDKINIKNVHKKHIVYAGSNVLLTKNE
```

```
                                           A-domain  <-|->     cTM3      <-|             |->         cTM4         ->|-> P-domain
HtarP5A NIEFDNKKLPISGCVGIVLKNGFSTYQGKLVRTIINSSEKVNSSSFDSLVFLFILLFFAICSSAYVIHQVVYSHHERNLYKVLLSVSHVITSVIPPEFPITLSLAVTISVVYLYSLKIYC
PrelP5A NNEFNNKKLPINGCVGIVLKNGFTTYQGKLVRTIINTSEKVNSSSIDSIIFLFILLLFSISSSAYVVYSVLHSNHERNLYKLILSVSHIITAVIPPEFPITLSLAVTISIVYLYNLKIYC
PgalP5A KNEFNNKKLPISGCVGIVLKNGFTTYQGKLVRTIINTSEKVNSSGIDSIVFLLILLLFSISSSAYVVYSVLQSNHERNLYKLVLSVSHIITAVIPPEFPITLSLAVTISIVYLYNLKIYC
PfalP5A NNKFNGKKLPVNGCIGIVLRSGFSTYQGKLVRTIINTSEKVNSSSIDSIIFLMILLLFSICSSAYVVYSVLKTNEERNLYKLLLSVSHIITAVIPPEFPITLSLAVTISIVYLYNMKIYC
PpraP5A NNKFNGKKLPVNGCIGIVLRSGFSTYQGKLVRTIINTSEKVNSSSIDSIIFLMILLLFSICSSAYVVYSVLKTNEERNLYKLLLSVSHIITAVIPPEFPITLSLAVTISIVYLYNMKIYC
PreiP5A NNKFNGKKLPVNGCLGIVLRSGFSTYQGKLVRTIINTSEKVNSSSIDSIIFLMILLLFSICSSGYVVYSVLKTNEERNLYKLLLSVSHIITAVIPPEFPITLSLAVTISIVYLYNMKIYC
PadlP5A NNKFNGKKLPVNGCIGIVLRSGFSTYQGKLVRTIINTSEKVNSSSFDSIIFLIILLLFSICSSAYVVYSVLKSNEERNLYKLLLSVSHIITAVIPPEFPITLSLAVTISIVYLYNMKIYC
PbilP5A NNKFNGKKLPVNGCLGIVLRSGFSTYQGKLVRTIINTSEKINSSSFDSIIFLMILLLFSICSSAYVVYSVLKTNEEKNLYKLLLSVSHIITAVIPPEFPITLSLAVTISIVYLYNMQIYC
PblaP5A NNIFNGKKLPVNGCLGIVLRSGFSTYQGKLVRTIINTSEKVNSSSFDSIIFLMILLLFSICSSGYVVYSVLKTNEERNLYKLLLSVSHIVTAVIPPEFPITLSLAVTISIVYLYNMKIYC
PgabP5A NNKFNGKNLPVNGCIGIVLRSGFSTYQGKLVRTIINTSDKVNSSSFDSIIFLIILLLFSICSSAYVVYSVLKSNEERNLYKLLLSVSHIITAVIPPEFPITLSLAVTISIVYLYNMKIYC
HsppP5A NNEFDKKKIPINGCIGIVLKNGFNTYQGKLVRTIINTSEKVNSSSTDSIIFLFILLLFSFCSCIYVVYTLVQLNTERNLYKILLSVSHIITAVIPPEFPITLSLSVTISIVYLYKLKIYC
PberP5A NTEFNNSKLPITGCVGIVLKNGFSTYQGKLVRTIINTSEKINSSSTDSIIFLFILLFFSITSCVYVVYTLLKTTNERNLYKILLSASHIITAVIPPEFPITLSLGVTISIVYLYNLKIYC
PyoeP5A NPEFNNSKLPITGCVGVVLKNGFSTYQGKLVRTIINTSEKINSSSTDSIIFLFILLFFSITSCVYVVYTILKTTDERNLYKILLSASHIITAVIPPEFPITLSLGVTISIVYLYNLKIYC
PchaP5A NTEFNNSKLPITGCVGVVLKNGFSTYQGKLVRTIINTSEKINSSSTDSIIFLFILLFFSITSCVYVVYTILKTTSERNLYKILLSASHIITAVIPPEFPITLSLGVTISIVYLYNLKIYC
PvinP5A NPEFNNSKLPITGCVGVVLKNGFSTYQGKLVRTIINTSEKINSSSTDSIIFLFILLFFSITSCVYVVYTILKTTNERNLYKILLSASHIITAVIPPEFPITLSLGVTISIVYLYNLKIYC
PgonP5A NNEFNNMKLPINGCIGVVLKNGFTTYQGKLVRTIINTSEKVNSSSTDSIIFLFILLLFSLSSCAYVIYTVSQSAHERNLYKLLLSVSHIITAVIPPEFPITLSLGVTISIIYLYNLKIYC
PvivP5A NDHFNNMKLPINGCVGIVLKNGFTTYQGKLVRTIINTSEKVNSSSIDSIIFLFILLLFSLSSCAYVIYTVLQATHERNLYKLLLSVSHIITAVIPPEFPITLSLGVTISIVYLYNLKIYC
PcoaP5A NDQFNNMKLPINGCIGIVLKNGFTTYQGKLVRTIINTSEKVNSSSIDSIIFLFILLLFSLSSCAYVVYSVLQSTHERNLYKLLLSVSHIITAVIPPEFPITLSLGVTISIVYLYNLKIYC
PcynP5A NDQFNNMKLPISGCIGIVLKNGFTTYQGKLVRTIINTSEKVNSSSIDSIIFLFILLLFSLSSCAYVVYTVLQATHERNLYKLLLSVSHIITAVIPPEFPITLSLGVTISIVYLYNLKIYC
PfraP5A NDEFNNMKLPINGCVGIVLKNGFTTYQGKLVRTIINTSEKVNSSSMDSIIFLFILLLFSLSSCAYVVYAVLQSTQERNLYKLLLSVSHIITAVIPPEFPITLSLGVTISIVYLYNLKIYC
PinuP5A NDQFNNMKLPVSGCIGIVLKNGFTTYQGKLVRTIINTSEKVNSSSIDSIIFLFILLLFSLSSCAYVAYTVLQGTHERNLYKLLLSVSHIITAVIPPEFPITLSLGVTISIVYLYNLKIYC
PknoP5A NDQFNNMKIPINGCIGIVLKNGFTTYQGKLVRTIINTSEKVNSSSIDSIIFLFILLLFSLSSCAYVIYSVLQSSHERNLYKLLLSVSHIITAVIPPEFPITLSLGVTISIVYLYNLKIYC
PbraP5A NNEFKNKKLPISGCIGIVIKNGFTTYQGKLVRTIINTSEKVNASSTDSIIFLIILLLFSLFACAYVVYSVLKANKERNLYGLLLSVSHIITAVISPEFPITLSLGVTISIVYLYNLKIYC
PmalP5A NNEFKNKKLPISGCIGIVIKNGFTTYQGKLVRTIINTSEKVNASSTDSIIFLIILLLFSLFACAYVVYSVLKANKERNLYGLLLSVSHIITAVISPEFPITLSLGVTISIVYLYNLKIYC
PovCP5A NNKFNGKKIPVNGCIGIVLKNGFTTYQGKLVRTIINTSEKVNTSSIDSIIFLLILLLFSFTSCVYVVYSVLKSSNEKNLYKLLLSASHIITAIIPPEFPITLSLGVTISIVYLYKQKIYC
PovWP5A NNKFNGKKIPVNGCIGIVLKNGFTTYQGKLVRTIINTSEKVNTSSIDSIIFLLILLLFSFTSCVYVVYSVLKSSNEKNFYKLLLSASHIITAIIPPEFPITLSLGVTISIVYLYKQKIYC
                                                                                                      kkkkk
```

```
                      P-domain  <-|->                                N-domain                                      VR5a
HtarP5A TEPFRLPFSGKAHICAFDKTGTLTENDMIVLGLFGFDENATTQITEIKEPVVNKQKIPFLAVSVIASCHSLCVVEGELLGDPLEKNSFLKLNCAMNSLDHTSVNTFIISR----------
PrelP5A TEPFRLPFSGKSKICAFDKTGTLTEDNMIVLGLFGLDNN-FKKINEIKESIVNKQKIPFLSLSVIAGCHSICTVNNQLLGDPLEKNSFLKFKCNMKCLDNTFVYTNNRKS----------
PgalP5A TEPFRLPFSGKSKICAFDKTGTLTEDNMIVLGLFGLDNN-FTKINELKESIVNKQRIPFLSLSVIAGCHSICTVNNKLLGDPLEKNSFLKLKCNMKSLDNTFVYTDNRKS----------
PfalP5A TEPFRLPFSGKTNICAFDKTGTLTEDNMIVLGLFGLDDN-LKRINEINESIINKQKIPFFSLSVIAGCHSICTLNNKLLGDPLEKNSFLKLKCVMKSLNHTYVYTNNINNNN--------
PpraP5A TEPFRLPFSGKTNICAFDKTGTLTEDNMIVLGLFGLDGN-LKRINEINESIINKQKIPFFSLSVIAGCHSICTLNNKLLGDPLEKNSFLKLKCVMKSLNHTYVYTNNNNNNN--------
PreiP5A TEPFRLPFSGKTNICAFDKTGTLTEDNMIVLGLFGLDGN-LKRINEINESIINKQKIPFFSLSVIAGCHSICTLNNKLLGDPLEKNSFLKLKCVMKSLNHTYVYTNNNNNNI--------
PadlP5A TEPFRLPFSGKTNICAFDKTGTLTEDNMIVLGLFGLDNN-YKKINEIKESIINKQKIPFFSLSVIAGCHSICSINNKLLGDPLEKNSFMKLKCVMKSLNHTYVNTNN-KNDD--------
PbilP5A TEPFRLPFSGKSNICAFDKTGTLTEDNMIVLGLFGLDNN-IKEINEIKESIINKQKIPFFSLSVIAGCNSICTVNNKLLGDPLEKNSFIKLKCVMKSLNHTYVYTNNKNVNNNNENKNND
PblaP5A TEPFRLPFSGKTNICAFDKTGTLTEDNMIVLGLFGLDNN-FKKINEIKESIINKQKIPFFSLSVIAGCHSICSVNNKLLGDPLEKNSFMKLKCVMKSLNSTYVYTNNKNVSNSNDNKNDD
PgabP5A TEPFRLPFSGKTNICAFDKTGTLTEDNMIVLGLFGLDNN-YKQINQIKESIINKQKIPFFSLSVIAGCHSICSVNNKLLGDPLEKNSFMKLKCVMKSLNHTYVHTNNNKNDD--------
HsppP5A TEPFRLPYSGKCTICAFDKTGTLTDDNMILLGLFGFDKN-CHKINKIKDSIINKQKVPFLSVAVIAGCHSLCIINNKILGDPLEKNCFTSLNCSMKGLDNTYVNMR--------RDIDDV
PberP5A TEPFRLPFSGKSNICAFDKTGTLTEDNMIVLGLFGLDNK-INHIYESNQSIINKQRIPFLSLAVISGCHSLCTVNNKLLGDPLEKNSFLKLNCNMKNLDNIYVRSSSYVSK-SGNDITSD
PyoeP5A TEPFRLPFSGKSNICAFDKTGTLTEDNMVVLGLYGLDNK-MNHIYESNKSIINKQRVPFLSLAVISGCHSLCTINNKLLGDPLEKNIFLKLNCNMKSIDNIYVRSSSYVSN-GGNDITSD
PchaP5A TEPFRLPFSGKSNICAFDKTGTLTEDNMIVLGLFGLDNK-INRIYESNQSIINKQRVPFLSLAVISGCHSLCTINNKLLGDPLEKNSFLKLNCSMKSLDNIFVRSSSYVSK-GGNDITSD
PvinP5A TEPFRLPFSGKSNICAFDKTGTLTEDNMIVLGLFGLDNK-INRIYESNQPIINKQRVPFLSLAVISGCHSLCTINNKLLGDPLEKNSFLKLNCSMKSLDNIFVRSSSYVSK-VGNDITSD
PgonP5A TEPFRLPFSGKSNICAFDKTGTLTEDNMIVLGLFGFDNN-TEKINEINESIINKQKVPFLSVAVIAGCHSICTVNNKLLGDPLEKNSFQKLKCMMRNLDNTYVYTNNNHI--------DG
PvivP5A TEPFRLPFSGKSNICAFDKTGTLTEDNMIILGLFGFDHN-TERINEINESIIGKQRVPFLSVAVIAGCHSLCTVNSKLLGDPLEKNSFQRLKCMMRSLDSTYVHTNNCQN----VSAATA
PcoaP5A TEPFRLPFSGKSNICAFDKTGTLTEDNMIVLGLFGFDQN-TERINEINESIISKQRVPFLSVAVIAGCNSICTVNNKLLGDPLEKNSFERLKCMMRSLDKTYVHTNNYPN--GAASAATS
PcynP5A TEPFRLPFSGKSNICAFDKTGTLTEDNMIILGLFGFDEN-TEKINEINESIISKQKVPFLSVAVIAGCHSICTVNNKLLGDPLEKNSFQRLKCMMKSLDKTYVYTNNCQN----AATATP
PfraP5A TEPFRLPFSGKSNICAFDKTGTLTEDNMIVLGLFGFDQN-TDRINEINESIISKQRLPFLSVAVIAGCHSICTVNNKLLGDPLEKNSFERLKCMMRSLDKTYVYTNNYQN----AAGATT
PinuP5A TEPFRLPFSGKSNICAFDKTGTLTEDNMIILGLFGFDQN-TERINEINESIIGKQRVPFLSVAVIAGCHSICTVNNKLLGDPLEKNSFQRLKCMMSSLDRTYVYTNNYQS----AAAATS
PknoP5A TEPFRLPFSGKSNICAFDKTGTLTEDNMIILGLFGFDKN-TERINEINESIISKQRVPFLSVAVIAGCHSICTVNNKLLGDPLEKNSFQRLKCMMRSLDKTYVHTNNYHNSASATSSATS
PbraP5A TEPFRLPFSGKSNICAFDKTGTLTEDNMIVLGLFGLDNN-IEKINDINKSIISRQKVPFFSVAVIAGCHSLCTVNNKLLGDPLEKNSFLKLKCMMKSLDSTYVYTNNKNN---------SS
PmalP5A TEPFRLPFSGKSNICAFDKTGTLTEDNMIVLGLFGLDNN-IEKINDINKSIISRQKVPFFSVAVIAGCHSLCTVNNKLLGDPLEKNSFLKLKCMMKSLDSTYVYTNNKNN---------SS
PovCP5A TEPFRLPFSGKSNICAFDKTGTLTEDNMIVLGLFGFDHY-TENIYEINESVINKQRVPFLSLAVIAGCHSICTVNNKLLGDPLEKNSFQKLKCVMKSLDNTYVHTIGGIG-------TTR
PovWP5A TEPFRLPFSGKSNICAFDKTGTLTEDNMIVLGLFGFDHY-TENIYGINDSIINKQRVPFLSLAVIAGCHSICTVNNKLLGDPLEKNSFQKLKCIMKSLDNTYVHTIGGVG-------TTR
                                 *
```

```
                       VR5a                                                    N-domain                                       VR5b
HtarP5A -----DRFGEKGAATKGKDIKKDDNTVHEKIIKTMVQDRFYIYKRFFFTSELQRMTCVVFHEGFGGDWYGEKYAYNEKKNKNVGNTTD--------------------------------
PrelP5A -------------------SNEINKKSGNYYSKNNYVENFQIYKRFFFTSELQRMTCIIAHEGYEYDWYGEEYEEEYES--KENDDN--------------------------------
PgalP5A -------------------ANEINKKSGNSSSKNNYVENFQIYKRFFFSSELQRMTCITFHEGYENDWFGEEYEEESTNNNKENKDK--------------------------------
PfalP5A -----NNNNNNNINNDNVVDKKYHKNNEKK-IKNQSLENFQIVKRFFFSSELQRMTCIILHEGSQHDWYGDEYETDTCDSDEQNEEQ--------------------------YKNTKQH
PpraP5A -----NNNNNNNINNDNVVDKKYQKNNEKK-IKNQSLENFQIVKRFFFSSELQRMTCIILHEGSQHDWYGDEYETDTCDSDEQNEEQ--------------------------YKNTKQH
PreiP5A -----NNNNNNNIKNNNIVDKKFEKKNEKK-IKNQSLENFQIVKRFFFSSELQRMTCIILHEGSQHDWYGDEYETDTCDSDEQNEEE--------------------------YKNTKQH
PadlP5A --TTIDENKNNDMFNLKNEKNKDQK--KIK-IKNESVENFQIVKRFFFSSELQRMTCILLHEGFQNDWYGDEYETDTCHSDDTN------------------------------ENIKRD
PbilP5A MNNLKNNNSNNNNSNNNIVDKKNGKNKDKK-IKNQSLENFKIVKRFFFSSELQRMTCIILHEGSQHDWYGEEYETDTCDSEERHEEQ--------------------------YKHTKQD
PblaP5A MIILKNNNNNNNNNNNILDKKNDINKDKK-IKNQSVENFQIVKRFFFSSELQRMTCIILHEGSQNDWYGDEYETDTCDSEEQNEEQ--------------------------YKNTKQD
PgabP5A --TTIDENKNNDMLNLKNEKNKDKKKINIK-IKNESVENFQIVKRFFFSSELQRMTCILLHEGSQNDWYGDEYETDTCHSDETN------------------------------ENIKSD
HsppP5A LGSDNNDVNTKKGDSSNITRTVSSRYKND---SGNFLEHFQICKRFFFSSELQRMTCILLHEGYERDWVGNQYTSDEEDVDNEFSNS------------------------YGYNIQEQYK
PberP5A GGNKFSEFFISSKSLTNLNKNISNSGKENRQVKNNMVENFFIYKRFFFSSDLQRMTCILQHTGYEGDWYGELYESDNDTNAEEFCN------------------------------DIENN
PyoeP5A GGNKFSEFFISSKSLTNLNKRISNFGKENRQVKNNLIEHFFIYKRFFFSSDLQRMTCILQHTGYEGDWFGDLYESDDDTNVDNFCN------------------------------DIENN
PchaP5A GGGKFSEFFISSKSLTNLNKKIPSFSKENREAKNNLIENFYIYKRFFFSSDLQRMTCILQHTGYEGDWYGDSYESDEEVTVEDFCN------------------------------DTESN
PvinP5A GGGKFSEFFISSKSLTNLNKKLPSFIKENREAKNNLIENFYIYKRFFFSSDLQRMTCILQHTGYEGDWYGDSYESDEEISVEDYCN------------------------------DTENN
PgonP5A SITHQSSGDRGSK-IIGMFGKKAPNPSLNEKKKKTCVENFQIYKRFFFSSELQRMTCIILHEGHEGDWYGEEFEEVTESSTCTNRSD------------------------------MEEKT
PvivP5A SVAAQGSGEKGGKSSMGMFSKKAPNTAANE-RKGPCVENFQIYKRFFFSSELQRMTCIILHEGYEGDWYGEEFEEAVNPSNAATAANPATAANPATAANPANAA---NPANAANPANAAN
PcoaP5A SETTQDSAEKGGKS-MGIFSKKTSNTMGSE-KKSPCVENFQIYKRFFFSSELQRMTCIILHEGYEGDWYGEEFEEVTDVATCAGSFN------------------------------GEIN
PcynP5A TANGQGSGEKGGKS-LGMFSKKVPNTTASE-KKSPCVENFQIYKRFFFSSELQRMTCIILHEGYEGDWYGEEYEEAAESATCVGSSN------------------------------GEKS
PfraP5A TTTAQSSGEKGGLS-MGMFLKKSSNTSVNE-KKSPCVENFQIYKRFFFSSELQRMTCIILHEGYEGDWYGEEFEEVTDPATCMSSSN------------------------------GDIS
PinuP5A ----QGSPEKGGKS-MVMFSKKAPNKTVSE-KKSPFVENFQIYKRFFFSSELQRMTCIILHEGYEGDWYGEEYEEAAEPATCVGSSN------------------------------GEKS
PknoP5A TVTTQGSEEKAGKS-LGIFSKKAPSTTIKG-EKGPCVENFQIYKRFFFSSELQRMTCIILHEGYECDWYGEEFEEVTDPGTCVDTPN------------------------------GEKN
PbraP5A SSSSNNNNSNDYKS-VNSFSKKLFSFHNEK-KKSPCLENFQIYKRFFFSSELQRMTCIVLHEGYEEDWYGEEYEESFTPCKHTTKVE-------------------------------SDK
PmalP5A SSSSNNNNSNDYKS-VNSFSKKLFSFHNEK-KKSPCLENFQIYKRFFFSSELQRMTCIVLHEGYEEDWYGEEYEESFTPCKHTTKVE-------------------------------SDK
PovCP5A GTSVWSSLWNNGHE-----NKEEVVQTNDR-RRNYQVENFQIVKRFFFSSELQRMTCIVQHEGYEGDWYGEEYDMGKTVPVEKTVPV------------------------EERTNVNSS
PovWP5A GTSVWSSLWNNGHE-----NKEEVVQTNDR-KKNYQVENFQIVKRFFFSSELQRMTCIVQHEGYEGDWYGEEYDMGKTAPVGKTAPVGKTAPVGKTAPVGKTAPVGKTASVEERTNVNSN
```

```
                                                                    VR5b
HtarP5A --GENRNKNSG-------------ETEMSGEGRG--------------------------------------------------------------------------------------
PrelP5A TISENVEIIDN-----------LKKNKNDNSNQ---------------------------------------------------------------------------------------
PgalP5A TINENMEILDN-----------LKKNKNNNNNQ---------------------------------------------------------------------------------------
PfalP5A VLRNNGHEIYKTTPYK-ESAQMLRKLKRKNNNEYDTESDDHADQNCDIHNNDIHNNDIPNNDIHNKDIHNKDIHNKDIRHNDVHHNDICYKHTEKE---------KKKKNRIKNILFVKK
PpraP5A VLRNNGHEIYKTTPYK-ESAQMLRKLKRKNNNEYDTESDDHADQNCDIHNNDIHNNDIHNKDIHNKDIHNKDIHNKDIRHNDVHHNDICYKHTEKE---------KKKKNRIKNILFVKK
PreiP5A VLHNNGHEIYKSTPYK-ESAEMLRKLKRKNNNEYDTQSDDDADQNYDLPNNDIHN-------------------------NDTHHNDICYKQTEKQ---------KKKKKKIKNILFVKK
PadlP5A VLHNNMNDISKSRCYK-DYDEILKKLKRKNNNENETQSEDDMDQND----------------------------------------YSNYNTTEKQ---------KKKKNKIKNILFVKK
PbilP5A VLDNNVNGIYKSTFYN-ECAENLRKLKRKNNIEYDTQSDDDADQNDDDDGHDDGD-------------------------------YHNYNPVEKQ---------KKKKNKIKNILFVKK
PblaP5A VLHNNGNERYNPTFYYNECAEKLRKLKRKNNYENNTQSDDNAEQNDNRHHKD-----------------------------------HNYNASEKQ---------NKKKKKIKNILFVKK
PgabP5A VLHNNMNDISKSTCYK-DYDEILRKLKRKNNNENETQSEDDMDQND----------------------------------------YSNYNPTEKQMMMKKKKNNNNKKNKIKNILFVKK
HsppP5A YINQNISDLQN-----------IQKDKKKIINKKNNEIANKDKNSK--------------------------------------------------------------SAKNNSSSSSSS
PberP5A IINKSLSNLNK-----------LKKKKKRNMTS------------------------------------------------------------------------------NSITGSTIN
PyoeP5A IINKNLSNLNK-----------LKKKKKKNISS------------------------------------------------------------------------------NSITGSTIN
PchaP5A IINKNLSNLNK-----------LKKKKKRNISS------------------------------------------------------------------------------SSITGSTIN
PvinP5A IINKNLSNLNK-----------LKKKKKWNIGS------------------------------------------------------------------------------NSITSSSIN
PgonP5A PNSENIAILEN-----------LKKKKKKNDSSK--------------------------------------------------------------------------------------
PvivP5A PCSENVATLEG-----------LKKKKKKNDSST--------------------------------------------------------------------------------------
PcoaP5A PCSENMATLEG-----------LKKKKKKNDSMK--------------------------------------------------------------------------------------
PcynP5A PCSENVATLEG-----------LKKKKKKNDSSK--------------------------------------------------------------------------------------
PfraP5A ACTENLTTLEG-----------LKKKKKKNDSSK--------------------------------------------------------------------------------------
PinuP5A PCSENVATLEG-----------LKRKKKKNDSSK--------------------------------------------------------------------------------------
PknoP5A PCNENVATLEG-----------LKKKKKKNDSSK--------------------------------------------------------------------------------------
PbraP5A IINENIAILDN-----------LKKRKKK-DNSK--------------------------------------------------------------------------------------
PmalP5A IINENIAILDN-----------LKKRKKK-DNSK--------------------------------------------------------------------------------------
PovCP5A IIIGNIAILNS-----------LKKKKKK-DSNK--------------------------------------------------------------------------------------
PovWP5A IIIGNIAMLDS-----------LKKKKKK-DSNK--------------------------------------------------------------------------------------
```

```
                                                                                N-domain  <-|->       P-domain
HtarP5A ------VFRREYLVVSKGSPEMMKKFLRHVPKDYDEILKKLTIKGYRVLCLAANVLNE-KYVSKNIKRELIEKDLIFCGFLTFICPIKNATLSYIDDIKGAGLKNIMITGDNALTACQVA
PrelP5A -------IIRQYLVVSKGSPEMMKKFLKKIPENYDQILNSLSIKGYRVICLAAKVLDS-KIINKSIKREEIEKDLHFCGFLAFICPIKKSTPKYILDIKRAGIKNIMITGDNALTACQVS
PgalP5A -------FTRQYLVVSKGSPEIMKKFLKKIPANYDQILDSLSIKGYRVLCLAANVLDN-GIISKNIKREEIEKDLYFCGFLAFICPIKKLTPNYILDIKRAGIKNIMITGDNALTACQVS
PfalP5A KKENKDKIVKQYLVVSKGSPEIMKRFLKKVPEHYDEVLNSLSIKGYRVLCLAVNILDN-NMYKENISREEVEKDLYFCGFLTFICPIKVTTPNYILHIKNAGIKNIMITGDNALTACQVS
PpraP5A KKENKDKIVKQYLVVSKGSPEIMKRFLKKVPEHYDEVLNSLSIKGYRVLCLAVNILDN-NMYKENISREEVEKDLYFCGFLTFICPIKVTTPNYILHIKNAGIKNIMITGDNALTACQVS
PreiP5A KKETNDKVVKQYLVVSKGSPEIMKRFLKKVPEHYDEVLNSLSIKGYRVLCLAVNVLDN-NMYKENISREEVEKDLYFCGFLTFICPIKVTTPNYILHIKNAGIKNIMITGDNALTACQVS
PadlP5A KKENKDKCVKQYLVVSKGSPELMKRFLKKIPEHYDEVLNSLSIKGYRVLCLAVNILDD-NMFNENIRREDVEKDLYFCGFLTFICPIKVSTPNYILNIKNAGIKNIMITGDNALTACQVS
PbilP5A KKETNNKVVKQYLVVSKGSPEIMKRFLKKVPEHYDAVLNSLSIKGYRVLCLAVNVLDN-NMYKENITREDVEKDLYFCGFLTFICPIKVTTPKYILHIKNAGIKNIMITGDNALTACQVS
PblaP5A KKENKDKVVKQYLVVSKGSPEIMKRFLKKVPEHYDDVLNSLSIKGYRVLCLAVNVLDN-NMYNENITREDVEKDLFFCGFLTFICPIKVSTPNYILHIKNAGIKNIMITGDNALTACQVS
PgabP5A KKENNDKCVKQYLVVSKGSPELMKRFLKKIPEHYDEVLNSLSIKGYRVLCLAVNILDD-NMFNENITREDVEKDLYFCGFLTFICPIKVSTPNYILNIKNAGIKNIMITGDNALTACQVS
HsppP5A SSNIKREYVKQYIVVSKGSPEIMKKFLKRVPDNYDEMLKQLSIRGYRVLCLAANVLNSSKIIPTNMKREEIEKNLYFCGLLAFKCPIKQFTPSYISEIKNAGIRNIMITGDIALTACQVS
PberP5A RSNHKNDMIKQYIVVSKGSPEIMKNFLKEIPENYDKILNSLSIKGYRVICLAANILDN-KIINKNLKREKIEKNLYFCGFLAFLCPIKISTPIYISDIKNAGIKNIMITGDNALTACQVA
PyoeP5A RSNNKNDMIKQYIVVSKGSPEIMKNFLKEIPENYDKILNSLSIKGYRVICLAANILDN-KIINKNLRREDVEKNLYFCGFLAFLCPIKISTPIYISDIKNAGIKNIMITGDNALTACQVA
PchaP5A RSSHKNDMVKQYIVVSKGSPEIMKNFLKEIPENYDKILTSLSIKGYRVICLAASILDN-KLIQKNLRREDVEKDLYFCGFLAFLCPIKISTPTYISDIKNAGIKNIMITGDNALTACQVA
PvinP5A RSSNKNDMVKQYIVVSKGSPEIMKNFLKEIPDNYDKILNSLSIKGYRVICLAANILDN-KIIQKNLRREDVEKDLYFCGFLAFLCPIKISTPIYISDIKNAGIKNIMITGDNALTACQVA
PgonP5A -----GETIKQYLAVSKGSPEMMKKFLKKIPQNYDQVLNSLSIKGYRVICLAANVLDS-KIISKNVKREDVEKNLHFCGFLTFICPIKASTPSYILDIKQAGLKNVMITGDNALTACQVS
PvivP5A -----SEPVKQYLAVSKGSPEMMKKFLKKIPPNYDQVLNSLSIKGYRVICLAANVLDN-KVVSKNVKREDVEKDLYFCGFLTFICPIKASTPSYILDIKQAGIKNVMITGDNALTACQVS
PcoaP5A -----NEPVKQYLAVSKGSPEMMKKFLKKVPANYDEMLNSLSIKGYRVICLAANVLDN-KVVSKNVKREDIEKDLHFCGFLTFICPIKVSTPSYILDIKQAGIKNVMITGDNALTACQVS
PcynP5A -----NEPVKQYLAVSKGSPEMMKKFLKKIPPNYDQVLNSLSIKGYRVICLAANVLDS-KVVSKNVKREDIEKDLHFCGFLTFICPIKAATPSYILDIKQAGIKNVMITGDNALTACQVS
PfraP5A -----NEPVKQYLAVSKGSPEMMKKFLKKIPPNYDQVLNSLSIKGYRVICLAANVLDN-KVVSKNVKREDVEKDLHFCGFLTFICPIKASTPRYILDIKQAGIKNVMITGDNALTACQVS
PinuP5A -----NEPVKQYLAVSKGSPEMMKKFLKKIPPNYDQMLNNLSIKGYRVICLAANVLDN-KVVSKNVKREDIEKDLHFCGFLTFICPIKASTPSYILDIKQAGIKNVMITGDNALTACQVS
PknoP5A -----NEPVKQYLAVSKGSPEMMKKFLKKIPANYDQVLNSLSIKGYRVICLAANVLDS-KVVSKNVKREDIEKDLHFCGFLTFICPIKASTPSYILDIKQAGIKNVMITGDNALTACQVS
PbraP5A -----NEPTKQYLVLSKGSPEMMKKFLKKIPENYDEVLNSLSIRGYRVLCLAANVLDN-KIISKNVRREDIEKDLFFCGFLTFICPIKTSTPEYIIDIKNAGIKNIMITGDNALTACQVA
PmalP5A -----NEPTKQYLVLSKGSPEMMKKFLKKIPENYDEVLNSLSIRGYRVLCLAANVLDN-KIISKNVRREDIEKDLFFCGFLTFICPIKTSTPEYIIDIKNAGIKNIMITGDNALTACQVA
PovCP5A -----NETVKQYLVVSKGSPEVMKKYLKKIPENYDIILNNLSIKGYRVLCLAANILDS-KIICKSIKREDVEKDLYFCGFLAFVCPIKKSTPSYILDIKNAGIKNIMITGDNALTACQVA
PovWP5A -----NETVKQYLVVSKGSPEVMKKYLKKIPENYDIILNNLSIKGYRVICLAANILDS-KVICKSIKREDVEKDLYFCGFLAFVCPIKKSTPNYILDIKNAGIKNIMITGDNALTACQVA
```

```
                 P-domain                                         VR6
HtarP5A QDVNLIPMVEKKDILIVKIVDND------------------------------------------------------SGTISEEDIKKGIQYLDAIVKISS--ELHAYKERLVNILENKN
PrelP5A QDVNITPSVKIKDILILKLKGNVSTNKRILKDIPNEMQHNKKNNTNEEEKEN------KNDINKKEENESMNENR-KYQTFTNEEKEEVITMLNHISKRNI--NLKNYVEKIIEIIE-NS
PgalP5A LDVNIIPPVKIKDILILKLKENYLTDKRVLNENSKDLNQDEKNYAIEEEKGN------KNEINKKEEHKSINENT-SNQTFTNKEKEEAIVILNNISKRNI--NLKIYVEKLIRIIE-NN
PfalP5A QDVNIVPKVTCKDILILKMNEVISYDLIGEKRTNMINMIDIMTTNN----NHTCDTNQSDDVCKNKINNVVKDLRYDHAIFKNDDIKDCIEFLTCLKNKETSVILKNHVENLIRIIEVNY
PpraP5A QDVNIVPKVTCKDILILKMNEVISYDLIKEKRTNMINMIDMMRTNN----NHICDTNQSDDVCKNKINNVVKDLRYDHAIFKNDDIKDCIEFLTCLKNKETSVILKNHVENLIRIIEVNY
PreiP5A QDVNIIPNVTCKEILILKLNEVISYDILEEKRT---NVIHMMTIHN----NHRCDTHESEDVCKNNMNSVVKDLRYDNGILKNEDIKDCIEFLTCLKNKETSVILKNHVENLIRIIEVNY
PadlP5A QDVNIIPDVANKHILILKLKEIS-YDMLNEKVINTLSVINKMMTNN----DDTCDDMS-----TNNTNNTVKDFSDDNKIFNKDDIKDSIEFLTCIKNKETNIFLKNHVENLIKIIEMNC
PbilP5A QDVNIIPNVTNKEILILKLKEVLSYDIIDEKRR---NMKSMMMINN----IRTCDINKNVDMSQNDMNLVLNDLRNDNGIFYNDNIKDSIEFLTYIKNKEMSVVLKKKIENLIKIIQVNY
PblaP5A QDVNIVPNVTSKEILILKMKEILSYDMISEQRT---NMLSMMMINNNNNNNNTCDVNKSGDMSPNIMNSVVKDIRYDNTIFKNDDIKDSIEFLKCIKNKETSVFLKKHIENLIKIIQVNY
PgabP5A QDVNIVPHVASKHILILKLKELS-YDMLNEKVTNTLSVINKMMTNN----DDTCDDMSS----TNNTNNIVKDFSDDNKIFNKDDIKDSIEFLTCLKSKETNIFLKNHVENLIKIIEMNC
HsppP5A LDVNIIAPIKCKDILILKLKNEQLENIANNSISCSSKEVTYDKDVFSKTPNQVEASDKKETITQPDVIYVQGEKN-EQGEKNEQEQKEIVNYLNKLGLQES--KKKKITDELIKILE-NN
PberP5A QDVNMIPSVKDKDILILKLKESYNK---GNNLYLKKCETS--LSVGDD---------KQFFIDEIKKNTISNE------DKIDILKKETISVLKNIYKEDL--NKKKIVEHLINIIE-NE
PyoeP5A QDVNMIPYVKDKDILILKISEHYNK---GNNLYLKKSETS--LSVDED---------KQISIGEIKKNTIFND------DQIDILKKEAITNLQNIYKENL--NKKKIIEHLINMIE-NE
PchaP5A QDVNMIPSVRDKDILILKKNESYNK---GNSLYLKRYETSSSLDISEDGR----GGNKQNIIDELKNITSLSE------DKIDILKKEAIFILQNIFKDDI--NKKKIADHLINMIM-KA
PvinP5A QDVNMIPSVKDKDILILKKNESYNK---GSSLHLKRYETSSSLSVSEN---------IHNFIDDIKNRRSLSD------DKIDVLKKEAIFILENIFKEDI--NKKKIAENLINVIK-KE
PgonP5A QDVNIVPSIKSKDILILKLREDYNT-----TSHGKKKLRSGTSFDPSHEMLQ---NEEKKFSGEKSPSDLADQ----EDIFSNNEKEKAIELIKNIPVHNK--TQNNAINKLIKIMKEKK
PvivP5A QDVNIVPPIKIKDILILKLREDY-------AAHGKRARAG----------------------GKAPSTGMTNE----GGIFTVCELEGAVEMIRNLPLQDK--SQQSAAEKLIKMMS-AK
PcoaP5A QDVNIVPSIKVKDILILKLREDY-------PMHG-----------------------------KTTSTRVTKD----GDTFNLCDLEGAIELIRNLPLEKK--SQQNAAEKLIKMIK-EK
PcynP5A QDVNIVPSIKIKDILILKLREDY-------AVHGKRMLAC----------------------GKATSTSLTNE----GNTFSVCERERAIELIRNLPLQNK--SQQNAAEKLIKMMK-EK
PfraP5A QDVNIVPSIKIKDILILKLREDY-------AVHGKRALVG----------------------GKGPWTSLTNE----GDIFSIYERESAIDLIRNLPLQNK--SQQNAAEKLIKMMK-EG
PinuP5A QDVNIVPSIKIKDILILKLREDY-------AVHGKMALVS----------------------GKTTSTSLTDE----GDTFSVFERERAIDLIRNLPLQNK--SQQNAAEKLIKMMN-EK
PknoP5A QDVNIVPSVKVKDILILKLRENY-------ALHG-----------------------------KTTSTSLTSD----GDTFNLCERERAIQLIRNLPLQMK--SQQKAGEKLIKMME-KK
PbraP5A QDVNIIPSVKSKDILILKLKEDY-------SLNDRRIRLG------PLSQSN---EAIEQRITKSLNDKISNDLI-GSEIFSNQDKVDAIEMLYSIGKSSM--VEKNVIEKVTKIIE-KN
PmalP5A QDVNIIPSVKSKDILILKLKEDY-------SLNDRRIRLG------PLSQSN---EAIEQRITKSLNDKISNDLI-GSEIFSNQDKVDAIEMLYSIGKSSM--VEKNVIEKVTKIIE-KN
PovCP5A QDVHIIPQVSSKDILILKIKENFVS-----EERKNNVEIFCP--------------------SRKLSSIGNGDES-SWAHFSNSEREDAIEMLNNIGKINT--TQRKIVDKVVQLIE-IN
PovWP5A QDVHIIPQVSSKDILILKIKENYVS-----EEGKNNIGMFSS--------------------STELSSISNGDES-SWTHFSKSEREDAIEMLNNIGKINT--TQRQIVHKIVQLVE-IN
```

```
                                                                                                   P-domain
HtarP5A TNMRKTPLFFFNREGNKMIP-FIENEEYLELCSNLFTLCITGDIIEYFIQKSFENEETSSVFDELIKRGSVFCRVSPKNKEIVIKTLNKIGNVTVMCGDGTNDMAALKAAHVGVSLLSVK
PrelP5A KNINNM-LYFINRENKKVLP-FIDNKEYIKLCAKLFSLCITGDIIEYFLQNTQKN---STLFDELIKRVHIFCRVSPKNKEIIIKTLNKLGNITIMCGDGTNDMAALKAAHVGVSLLSIK
PgalP5A KNINNI-LYFVNRENKKVLP-FIDSEEYIKLCGKLFSMCITGDIIEYFLQNIQKH---FTLFDELIKRVHVFCRVSPKNKEIIIKTLNKLGNITIMCGDGTNDMAALKAAHVGISLLSIK
PfalP5A NKCSNI-LYFMNRENKKILP-FIHNIEYIKVCSEIFSLCITGDIIDYFLEVYKNN---LHIFNELIRGVHIFCRMSPKNKEIIIKTLNKIGYITIMCGDGTNDMAALKAAHVGVSLLSIK
PpraP5A NKCSNI-LYFMNRENKKILP-FIHNIEYIKVCSEIFSLCITGDIIDYFLEVYKNN---LHIFNELIRGVHIFCRMSPKNKEIIIKTLNKIGYITIMCGDGTNDMAALKAAHVGVSLLSIK
PreiP5A NKCSNI-LYFMNRENKKILP-FIHNIDYIKVCSEIFSLCITGDIIDYFLEVYKNN---LHIFNELIRGVHIFCRMSPKNKEIIIKTLNKIGYITIMCGDGTNDMAALKAAHVGVSLLSIK
PadlP5A DKCSNI-LYFMNRENKKILP-FIDNIDYIKGCGALFSLCITGDIIDYFLQVYKNN---LNIFNELIRGVYIFCRMSPKNKEIIIKTLNKIGYITIMCGDGTNDMAALKAAHVGVSLLSIK
PbilP5A NKCSNI-LYFMNRENKKILP-FIDNIDYIKGCSELFSLCITGDIIDYFLQEYKNN---LNIFNELIRGGYIFCRMSPKNKEIIIKTLNKIGYITIMCGDGTNDMAALKAAHVGVSLLSIK
PblaP5A NKCSNI-LYFMNRENKKILP-FIDNIDYIKGCSELFSLCITGDIIDYFLEVYKNN---LNIFNEVIRGVHIFCRMSPKNKEIIIKTLNKIGNITIMCGDGTNDMAALKAAHVGVSLLSIK
PgabP5A NKCSNI-LYFMNRENKKILP-FIDNIDYIKSCGALFSLCITGDIIDYFLQVYKNN---LNIFNELIRGVYIFCRMSPKNKEIIIKTLNKIGYITIMCGDGTNDMAALKAAHVGVSLLSIK
HsppP5A KNVNSM-LNFVNRENKKILP-FINNEEYIKLCEKLFTLCLTGDIIDYFLKTYTNN---IYIFDELIRHVQIYCRVSPKNKEIIIKTLNKLGNITIMCGDGTNDMAALKAAHVGISLLNIK
PberP5A KNINNM-LFFSNRENKKIIPFFIGNEEYIKLCSELFTLCITGNIIEYFLRKYKNN---INLIDMLINKVHIFCRVSPKNKEIIIKTLNKLGNITIMCGDGTNDMAALKAAHVGISLLSIK
PyoeP5A KNINNM-LFFSNRENQKIIPFFIGNEEYIKLCSELFTLCITGNIIEYFFREYKNN---INLIDMLINKVHIFCRVSPKNKEIIIKTLNKLGNITIMCGDGTNDMAALKAAHVGVSLLSIK
PchaP5A KNVNTM-LFFSNRENKNVIPFFIGNEEYIKLCSELFTLCITGDIIEYFFREYQNN---INLIDMLINKVHIFCRVSPKNKEIIIKTLNKLGNITIMCGDGTNDMAALKAAHVGVSLLSIK
PvinP5A KNVNDI-LFFSNREDKKIIPFFIGNEEYIKLCSELFTLCITGDIIEYFFMEYQNN---INLIDMLINKVHIFCRVSPKNKEIIIKTLNKLGNITIMCGDGTNDMAALKAAHVGVSLLSIK
PgonP5A KNVNKM-VYFINRENKKMLP-FIDCEEYIKLCEELFTLCITGDIIEYFLNNYQND---LTIFDELIKRVLIFCRVSPKNKEIIIKTLNKLGNITIMCGDGTNDMAALKAAHVGVSLLSIK
PvivP5A KNVSNV-LYFINRENKKMLP-FIECEEYLKSCEQLFTLCITGDIIEHFLSTYQND---LGLFDELIKRGLIFCRVSPKNKEIIIKTLNKLGNITIMCGDGTNDMAALKAAHVGVSLLSIK
PcoaP5A KNVSNV-VYFINRENKKMLP-FIECEEYIKLCEQLFTLCITGDIIEHFLTKYQND---MGIFDELIKRGVIFCRVSPKNKEIIIKTLNKLGNITIMCGDGTNDMAALKAAHVGVSLLSIK
PcynP5A KNVSNV-LYFINRENKKMLP-FIECEEYIKLCEELFTLCITGDIIEYFLSSYQND---LAIFDELIKRVLIFCRVSPKNKEIIIKTLNKLGNITIMCGDGTNDMAALKAAHVGVSLLSIK
PfraP5A RNVSNV-VYFINRKNKKMLP-FIECEEYIKLCEELFTLCITGDIIEHFLSRYQND---LALFDELIKRGLIFCRVSPKNKEIIIKTLNKLGNITIMCGDGTNDMAALKAAHVGVSLLSIK
PinuP5A KNISNV-VYFINRENKKMLP-FIECEEYIKLCEQLFTLCITGDIIEHFLSSYQND---LALFDELIKRALIFCRVSPKNKEIIIKTLNKLGNITIMCGDGTNDMAALKAAHVGVSLLSIK
PknoP5A K-ASNV-LYFINRENKKMLP-FIECEEYIKLCEQLFTLCITGDIIEYFLTNYEND---MGIFDELIKRGLIFCRVSPKNKEIIIKTLNKLGNITIMCGDGTNDMAALKAAHVGVSLLSIK
PbraP5A KNVSSR-IYFIDRENKKMLP-FIDNEEYIKLCEHIFSLCITGDIIDYFLNNYQND---LTLFNELIKRVHIFCRVSPKHKEIIIKTLNKLGNISIMCGDGTNDMAALKAAHVGISLLSIK
PmalP5A KNVSSR-IYFIDRENKKMLP-FIDNEEYIKLCEHIFSLCITGDIIDYFLNNYQND---LTLFNELIKRVHIFCRVSPKHKEIIIKTLNKLGNISIMCGDGTNDMAALKAAHVGISLLSIK
PovCP5A KNVNYP-LYFINRENKKVLP-FICNEQYVKLCEKVFTLCITGDIIEYFLTNHQND---LIPFYELIKRVHIFCRVSPKNKEIIIKTLNKMGNITIMCGDGTNDMAALKAAHVGVSLLSIK
PovWP5A KNVNYP-LYFINRENKKVLP-FICNEQYIKLCEKVFTLCITGDIIEYFLTNHQND---LIPFYELIKRVHIFCRVSPKNKEIIIKTLNKMGNITIMCGDGTNDMAALKAAHVGVSLLSIK
```

```
                                                                         VR7
HtarP5A MCGKGG---------------------NERVAINRQNRHDMQNLNGRNGN----------PLFKKMKHFMQPKNKVSENVGELEELKKIVENKELLRLNEN---------------GKKI
PrelP5A IGYKNS-----------------------NLSSNVKNNSNYLC----------------------NNKYYNNSVYVNENY-----LKTMYDNLEIKNRSTG--------------MHNNM
PgalP5A ISYKNN-----------------------NLSNNLKNNESYLN----------------------NNRDYNNSLKTYENH-----LKNAYDNLEIKNRSNV--------------MHNNM
PfalP5A ISYKNR----------------------DGNRKSVLNDDRKSLLNNHNNM-------------RMMNMYGDGRVKSVYDN-----LRASYSEARNIINNNSNN------------LGGIN
PpraP5A ISYKNR----------------------DGNRKSVLNDDRKSLLNNHNNM-------------RMMNMYGDGRVKSVYDN-----LRASYSEARNIINNNSNN------------LGGIN
PreiP5A ISYKNR----------------------DGNKRSVLNDDRKSLLNNNNN-----------------NMYGDGRVKSVYDN-----LRASYSEARNIINNNSNN------------LGGIN
PadlP5A VSYKNK----------------------DGKNKKMLYDERKNSLNNNN--------------MMMMNMYGDGRSRSLYDN-----LRASYAEARNIINNNNNN-------SGTNCGMGLK
PbilP5A VSYKNR----------------------DGYTRNVLNNERKNLLNNNNNDNNNN-------MMMMANMYSNGRVRSVYDN-----LKASYSEARNIINNSSNNC-----------VGGIN
PblaP5A ISYKNR----------------------DGYNRNMLNDHRKNLLTNNNNNNNNNN------MTMMMNMYGDRRIRSVYDN-----LRATYSEARNIINNNSNNC-----------VGGIN
PgabP5A VSYKNK----------------------DGKNKKMLYDERKNSLNNNNNN------------MMMMNMYGDGRSRSLYDN-----LRASYAEARNIINNNNNNNNNNNNNSGVNCGVGLK
HsppP5A MVYTNKQDKKTAYSNSSNANRLLHYNTQNISTGSSSYGISSNILNNKSDMVSRSTYFMDQHVNNQLYDNNSKSINNQPYDSTYLEKNLGSNYGTINNYLNKGTYPITNYTTTNSCNRNNA
PberP5A ICYKNK-------------------DLKTKIDGNSNYMHGKSIIEHR----------------DEYYENNVPNINNCINDYN----NVRAKYGNIRPYNLD----------------NNM
PyoeP5A ICYKNK-------------------DLKNKIDENNNYIHNKSIIEHQ----------------NGNYEKQIPNINNCISDYK----NVCAKYGNIPQYNLD----------------NNM
PchaP5A ICYKNK-------------------DSKSKIRGSNSYMHGNSILDHQ----------------NEYYRNRVPNVNNCISDYN----NVCAKYGNIPPYNPD----------------NNM
PvinP5A ICYKNK-------------------DVKSKIRGSNNYMHTNSILDHQ----------------SDYYRNRVPNVNNCINDYN----NVVAKYGNIPPYNPD----------------NNM
PgonP5A ITYKNT-----------------------RSDNSNINITNQLNRGNIN-------------PNTTNYNNGNNG-AIQNYEQR-----LRSVYDNLNAQYNAAS---------ANGGNNLA
PvivP5A ISYKSG-----------------------RPDGG--------------------------------YPGDLTGGATRNYEQR-----LRTAYDNLNAQYNAAS---------ASGVSNAA
PcoaP5A ISYKSG-----------------------RPDGGGVNMATQMNGGHTNALLGG--------THVGGYPGNFNGGTVSNYEQR-----LRTAYDNLNAQYNAAS---------ASGASNTA
PcynP5A ISYKSG-----------------------RPDGGGANMATQFNGGRTNALVGG--------PHVGAYPGDFNAGAARNYEQR-----LRTAYDNLNAQYNATS---------ASGGSNVA
PfraP5A ISYKSG-----------------------RAHGGHANMDRHLNAGRMNALVDG--------PHVGGYPCDFSGGASRNYEQR-----LRAAYDKLNAQYNAAS---------ASGGSNMA
PinuP5A ISYKSG-----------------------RPDGGGANMATQLNGGCTSALVGD--------PHVGGYPGDLHGGATRNYEQR-----LRNAYDNLNAQYNASY---------ASGGSNVA
PknoP5A ISYKSA-----------------------RPDDAGANMATQLNGEHANALLGG--------THVGSYPGHFNGGATRNYEHR-----LRSAYDNLNAQYNAAS---------ASGANNLA
PbraP5A ISYKNK-----------------------EIDNSNNNKQYNAN-------------------SLNTYDKGYNN-YYANSENH-----FRNPYNNLYAPYNSSST------TTASSGSSNM
PmalP5A ISYKNK-----------------------EIDNSNNNKQYNAN-------------------SLNTYDKGYNN-YYANSENH-----FRNPYNNLYAPYNSSST------TTASSGSSNM
PovCP5A ISYTNR-----------------------KTHSDCSSTTTMLP------------------------HGQNNSDNVSAYEKR-----LRSAYEKLMAQDAAAG------------VANGP
PovWP5A ISYTNR-----------------------KTHSDCSSNATMLP------------------------HGQNNSDTVSAYEKR-----LRSAYEKLMAQDAAAG------------VANGP
        arm (orange box)
```

```
                                                        P-domain   <-|->        cTM5         <-|         |->    cTM6    <-|
HtarP5A LRQRLQHMVLYKEEKEQFEKLLQSADDSLPLVKLGEASIASPFTYKGSDIKCVKDIISCGRCALSKVIMMYKLMIINSLITAFSVSILTLDGVKIGDVQTTAISLVYTALVVLMSKATPL
PrelP5A NYKYYEQMKLYNERKKKLENMMQSMDDSLPLIKLGEASIASPFTYKGNDIKCVKEIICCGRCALSKVIMMYKLMIINSLITAFSVSILTLDGVKLSDAQTTIISLLYTSLIVLISKATPL
PgalP5A NYRYYEQMKLYNERKKKIESMMQSMDDSLPLIKLGEASIASPFTYKGNDIKCVKEIICCGRCALSKVIMMYKLMIINSLITAFSVSILTLDGVKLSDAQTTIISLLYTTLIVLISKSTPL
PfalP5A FRRSYEQMKLYNEKKKELDKMLQSLDDSLPLIKLGEASIASPFTYKGNDIKCVKEIISCGRCALSKVIMMYKLMIINSLITAFSVSILTLDGVKLSDAQTTIISLLYTCLIVLISKTSPL
PpraP5A FRRSYEQMKLYNEKKKELDKMLQSLDDSLPLIKLGEASIASPFTYKGNDIKCVKEIISCGRCALSKVIMMYKLMIINSLITAFSVSILTLDGVKLSDAQTTIISLLYTCLIVLISKTSPL
PreiP5A FRRSYEQMKLYNEKKKELDKMLQSLDDSLPLIKLGEASIASPFTYKGNDIKCVKEIISCGRCALSKVIMMYKLMIINSLITAFSVSILTLDGVKLSDAQTTIISLLYTCLIVLISKTSPL
PadlP5A FRRSYEQMKLYNEKKKELEQMLQSLDDSLPLIKLGEASIASPFTYKGNDIKCIKEIISCGRCALSKVIMMYKLMIINSLITAFSVSILTLDGVKLSDAQTTVISLLYTSLIVLISKTSPL
PbilP5A FRRSYEQMKLYNEKKKELEKMLQSFDDSLPLIKLGEASIASPFTYKGNDIKCVKEIISCGRCALSKVIMMYKLMIINSLITAFSVSILTLDGVKLSDAQTTIISLLYTSLIILISKTSPL
PblaP5A IRGSYEQMKLYNEKKKELEKMLQSFDDSLPLIKLGEASIASPFTYKGNDIKCVKEIISCGRCALSKVIMMYKLMIINSLITAFSVSILTLDGVKLSDAQTTIISLLYTSLIVLISKTSPL
PgabP5A FRRSYEQMKLYNEKKKELEQMLQSLDDSLPLIKLGEASIASPFTYKGNDIRCIKEIISCGRCALSKVIMMYKLMIINSLITAFSVSILTLDGVKLSDAQTTVISLLYTSLIVLISKTSPL
HsppP5A NRRYQEQMKLYNVRKKELENMMQSMDDSLPLIKLGEASIASPFTYKGNDISCVKEIICCGRCALSKVIMMYKLMIMNSLITAFSVSILTLDGVKLSDAQTTIISLLYTSLIVLLSKTTPL
PberP5A NAKYYEQIKLYNERKKQLENMMKTMDDSLPLIKLGEASIASPFTYKGNDIKCVKEIISCGRCALSKVIMMYKLMIINSLITAFSVSILTLDGVKLSDAQTTVISLLYTTFIVLISKTTPL
PyoeP5A NAKYYEQIKLYNERKKQLENMMKNMDDSLPLIKLGEASIASPFTYKGNDIKCVKEIISCGRCALSKVIMMYKLMIINSLITAFSVSILTLDGVKLSDAQTTIISLLYTSFIVLISKTTPL
PchaP5A NAKYYEQIKLYNERKQQLENMMKTMDDSLPLIKLGEASIASPFTYKGNDIKCVKEIIACGRCALSKVIMMYKLMIINSLITAFSVSILTLDGVKLSDAQTTVISLLYTTFIVLISKTTPL
PvinP5A NAKYYEQMKLYNERKQQLENMMKTMDDSLPLIKLGEASIASPFTYKGNDIKCVKEIISCGRCALSKVIMMYKLMIINSLITAFSVSILTLDGVKLSDAQTTVISLLYTTFIVLISKTTPL
PgonP5A SARYYEQMKLYNERKKQLENMMQSMDDSLPLIKLGEASIASPFTYKGNDIKCIKEIICCGRCALSKVIMMYKLMIINSLITAFSVSILTLDGVKLSDAQTTVISLLYTSLIVLISKTTPL
PvivP5A NAKYYEQMKLYNERKRQLEHMMQSMDDSLPLIKLGEASIASPFTYKGNDIKCVKEIICCGRCALSKVIMMYKLMIINSLITAFSVSILTLDGVKLSDAQTTVISLLYTSLIVLISKTAPL
PcoaP5A NAKYYEQMRLYNERKRQLEQMMQSMDDSLPLIKLGEASIASPFTYKGNDIKCVKEIICCGRCALSKVIMMYKLMIINSLITAFSVSILTLDGVKLSDAQTTVISLLYTSLIVLISKTAPL
PcynP5A NAKYYEQMKLYNERKRQLEHMMQSMDDSLPLIKLGEASIASPFTYKGDDIKCVKEIICCGRCALSKVIMMYKLMIINSLITAFSVSILTLDGVKLSDAQTTVISLLYTSLIVLISKTAPL
PfraP5A NAKYYEQMKLYNERKRQLENMMQSMDDSLPLIKLGEASIASPFTYKGNDIKCVKEIICCGRCALSKVIMMYKLMIINSLITAFSVSILTLDGVKLSDAQTTVISLLYTSLIVLISKTAPL
PinuP5A NAKYYEQMKLYNERKRQLEHMMQSMDDSLPLIKLGEASIASPFTYKGNDIKCVKEIICCGRCALSKVIMMYKLMIINSLITAFSVSILTLDGVKLSDAQTTIISLLYTSLIVLISKTAPL
PknoP5A NAKYYEQMKLYNERKKQLEHMMQSMDDSLPLIKLGEASIASPFTYKGNDIKCVKEIICCGRCALSKVIMMYKLMIINSLITAFSVSILTLDGVKLSDAQTTVISLLYTSLIVLISKTAPL
PbraP5A NAKYYEQMKKYNERKQALENMMQSIDDSLPLIKLGEASIASPFTYKGNDIKCVKEIICCGRCALAKVIMMYKLMIINSLITAFSVSILTLDGVKLSDAQTTVISLLYTSLIVLISKTTPL
PmalP5A NAKYYEQMKKYNERKQALENMMQSIDDSLPLIKLGEASIASPFTYKGNDIKCVKEIICCGRCALAKVIMMYKLMIINSLITAFSVSILTLDGVKLSDAQTTVISLLYTSLIVLISKTTPL
PovCP5A GAKYYEQMMLYNERKKKLENMMQSMDDSLPLIKLGEASIASPFTYKGNDIKCVKEIICCGRCALSKVIMMYKLMIINSLITAFSVSILTIDGVKLSDAQTTVISLLYTSLIVLISKTAPL
PovWP5A SAKYYEQMMLYNERKKKLENMMQSMDDSLPLIKLGEASIASPFTYKGNDIKCVKEIICCGRCALSKVIMMYKLMIINSLITAFSVSILTIDGVKLSDAQTTVISLLYTSLIVLISKTAPL
```

```
                       |->       sTM7      <-|                   |->       sTM8      <-|        |->      sTM9      <-|
HtarP5A ETMSNYCPPNSLFNPSVILSLIVQVVVHFTVLIYGWKLAASFRSDDYVPDLKGEFSPNLVNTCIYYLIYIINLSIFSCNYEGLPFMIPLHKHKEIMCIFTFNFILLFVQIMEIFPFLNNF
PrelP5A KNISNYSPPNSLFNISVITSLIFQVFIHFSILIYGWKLSSSYRPPDYVPDLKGEFTPNLVNTCIYYLIYCINLSIFSCNYEGLPFMTPIHKNKEIVYIFIVNFIFLFSLVMNIFPFLNHF
PgalP5A KTISNYSPPTSLFNISVIASLIFQVFVHFSILVYGWKLSSSYRQPDYVPDLKGEFTPNLVNTCIYYLIYCINLSIFSCNYEGLPFMTPIHKNKEIVYIFIVNFIFLFSLVMNIFPFLNYF
PfalP5A KNITNYSPPNSLFNFSVIISLLSQIIIHFSILIYGWKLACVYREINYIPDIKGDFIPNLVNTCIYYLIYCINLSIFSCNYEGLPFMVPIHKNKEIVYIFAVNFFFLFVLVMDIFPFLNYF
PpraP5A KNITNYSPPNSLFNFSVIISLLSQIIIHFSILIYGWKLACVYREINYIPDIKGDFIPNLVNTCIYYLIYCINLSIFSCNYEGLPFMVPIHKNKEIVYIFAVNFFFLFVLVMDIFPFLNYF
PreiP5A KNITNYSPPNSLFNFSVIISLLSQIIIHFSILIYGWKLACAYREINYIPDIKGDFIPNLVNTCIYYLIYCINLSIFSCNYEGLPFMVPIHKNKEIVYIFAVNFFFLFVLVMDIFPFLNYF
PadlP5A KSITNYKPPNSLFNFSVIISLLLQIIIHFSILIYGWKLACTYREINYIPDIKGEFKPNIVNTCIYYLIYCINLSIFSCNYEGLPFMIPIHKNKEIVYIFVVNFFFLLLLIMDLFPYLNYF
PbilP5A KNITNYTPPNSLFNFSVIISLLSQIFIHFYILIYGWKLACSYRDINYIPDIKGDFIPNLVNTCIYYLIYCINLSIFSCNYEGLPFMIPIHKNKEIVYIFVVNFFFLFVLVMDIFPFLNYF
PblaP5A KNITNYSPPNSLFNISVIISLLSQIIIHFFILIYGWKLACAYREINYIPDIKGEFIPNLVNTCIYYLIYCINLSIFSCNYEGLPFMIPIHKNKEIVYIFVVNFFFLFVLVMDIFPFLNYF
PgabP5A KSITNYKPPNSLFNFSVIISLLLQIIIHFSILIYGWKLACTYREINYIPDIKGEFKPNIVNTCIYYLIYCINLSIFSCNYEGLPFMIPIHKNKEIVYIFLVNFFFLLLLIMDLFPYLNYF
HsppP5A KEISRYAPPSSLFNLSVILSLICQIACHFFLLIVGWKMACSLREPDYMPDLKSDFTPNFVNTCIFYFIYCINLSIFSCNYEGLPFMPPIHKNKEMVYIFIGNYIFLFILIMNLCPFINHF
PberP5A ESISSYAPPNSLFNITVVLSLLCQVIVHFSILIYGWIVASSFRDPYYVPDLKGDFSPNIVNTCIYYLIYCINLSIFLCNYEGLPFMLPLHKNKELVYIFIGNFFFLFLNIMNIVPYINYF
PyoeP5A ESISSYAPPNSLFNITVVLSLLCQIIVHFSILIYGWIIASSFRGPDYVPDIKGDFSPNIVNTCIYYLIYCINLSIFLCNYEGLPFMLPLHKNKELVYIFIGNFFFLFLNIMNIVPYINYF
PchaP5A ENISSYAPPNSLFNITVVLSLICQVIVHFSILVYGWIVASSFRGPDYVPDLKGEFSPNIVNTCIYYLIYCINLSIFLCNYEGLPFMLPLHKNKELVYIFIGNFIFLFLNVMNIFPYINYF
PvinP5A ENISSYAPPNSLFNITVVLSLICQVIVHFSILIYGWIVASSYRGPDYVPDLKGEFSPNIVNTCIYYLIYCINLSIFLCNYEGLPFMLPLHKNKELVYIFIGNFFFLFLNVMNIVPYINYF
PgonP5A KSISNYSPPNSLFNISVMSSLISQVFIHFSILIYGWKLACSYRQPDYVPDLKGDFSPNLVNTCIYYLIYCINLSIFSCNYEGLPFMTPIHKNKEIVYIFIANFIFLFALVMNIIPDLNHF
PvivP5A KNISNYSPPNSLFNISVMSSLISQVFIHFSILIYGWKLACSYRQPDYVPDLKGDFSPNLVNTCIYYLIYCINLSIFSCNYEGLPFMTPIHKNKEIVYIFAVNFIFLFALVMNIVPYLNYF
PcoaP5A KNISNYSPPNSLFNISVMSSLISQVFVHFSILIYGWKLACSYREPNYIPDLKGDFSPNLVNTCIYYLIYSINLSIFSCNYEGLPFMTPIHKNKEIVYIFAVNFIFLFALVMNIVPYLNHF
PcynP5A KNISNYSPPNSLFNISVMSSLISQVFIHFSILIYGWKLACSYRQPDYIPDLKGDFSPNLVNTCIYYLIYCINLSIFSCNYEGLPFMTPIHKNKEIVYIFAVNFIFLFALVMNIVPYLNYF
PfraP5A KNISNYSPPNSLFNISVMCSLISQVFIHFSILIYGWKLACSYRQPNYVPDLKGDFSPNLVNTCIYYLIYCINLSIFSCNYEGLPFMTPIHKNKEIVYIFAMNFIFLFALVMNIIPYLNYF
PinuP5A KDISNYSPPNSLFNISVMSSLIFQVFIHFSILIYGWKLACSYRQADYIPDLKGDFSPNLVNTCIYYLIYCINLSIFSCNYEGLPFMTPIHKNKEIVYIFAVNFIFLFALVMNIVPYLNYF
PknoP5A KNISNYSPPNSLFNISVMSSLISQVFVHFSILIYGWKLACSYRESDYIPDLKGDFSPNLVNTCIYYLIYCINLSIFSCNYEGLPFMTPIHKNKEIVYIFGINFIFLFALVMNIIPYLNYF
PbraP5A ENISNYSPPNSLFNISVIASLICQVFVHFFILIYGWKLASSYREPDYVPDLKGDIDPNIVNTCIYYLIYCINLSIFSSNYEGLPFMIPLHKNKEIIFIFISNIIFLFSLVLNILPFLNYF
PmalP5A ENISNYSPPNSLFNISVIASLICQVFVHFFILIYGWKLASSYREPDYVPDLKGDIDPNIVNTCIYYLIYCINLSIFSSNYEGLPFMIPLHKNKEIIFIFISNIIFLFSLVLNILPFLNYF
PovCP5A KDISKYAPPNSLFSISVIVSLISQVIIHFSILTYGWKLACSYRQPDYVPDLKANFTPNLVNTCIYYLIYSINLSIFSCNYEGLPFMAPIHKNKEIVYIFIANFLFLFSLVMNLFPFLNNF
PovWP5A KDISKYAPPNSLFSISVIVSLISQVIIHFSILTYGWKLACSYRQPDYVPDLKANFTPNLVNTCIYYLIYSINLSIFSCNYEGLPFMAPIHKNKEIVYIFIANFLFLFSLVMNLFPFLNHF
```

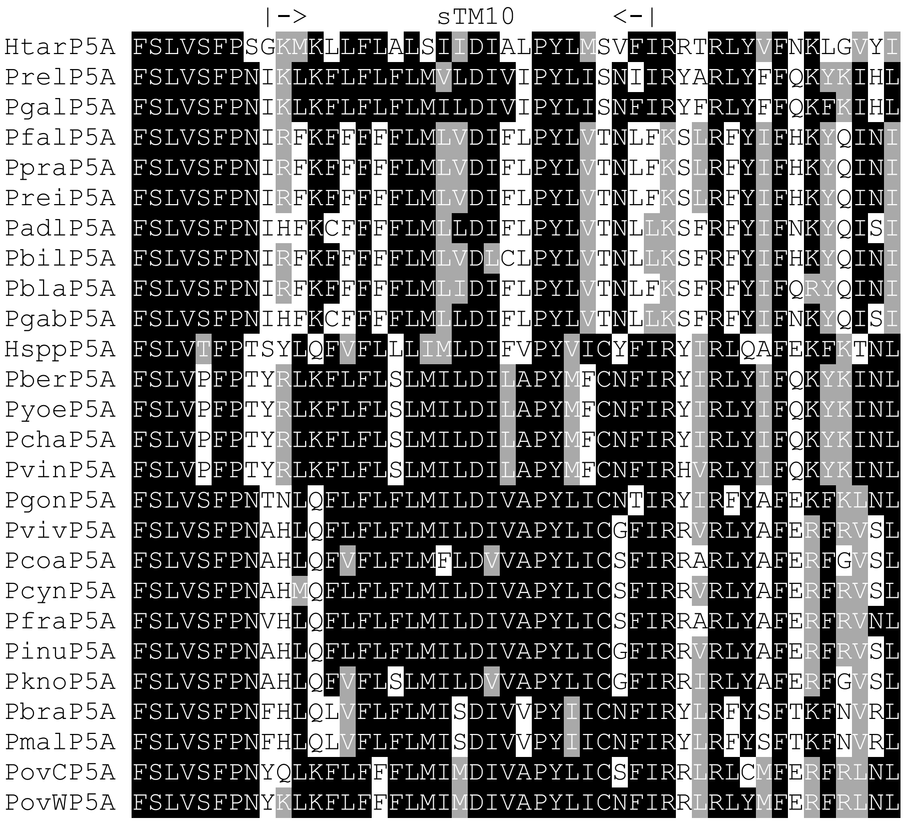

(a) Type-P5A ATPases from haemosporidian species (Supplementary Table 1) were aligned with ClustlW within Mega 11. The variable regions were adjusted manually to compress the sequences. Black shading is identical residues and grade shading is similar residues. The various domains are based on the 3-dimensional structure of the *P. falciparum* and *P. relictum* proteins. The kkkkk below the alignment is the kink in cTM4 and the asterisk denotes the phosphorylated aspartate residue. The residues in *P. relictum* predicted to form the 'arm' of the P-domain are boxed in orange. (b) Unrooted minimum evolution tree generated from the alignment after removing variable sequence. (c) Rooted minimum evolution tree using *Haemoproteus* (Htar) as an outgroup from the same alignment. The eight clades are labeled in blue and bootstrap values are shown at the nodes in the minimum evolution tree.

(b)

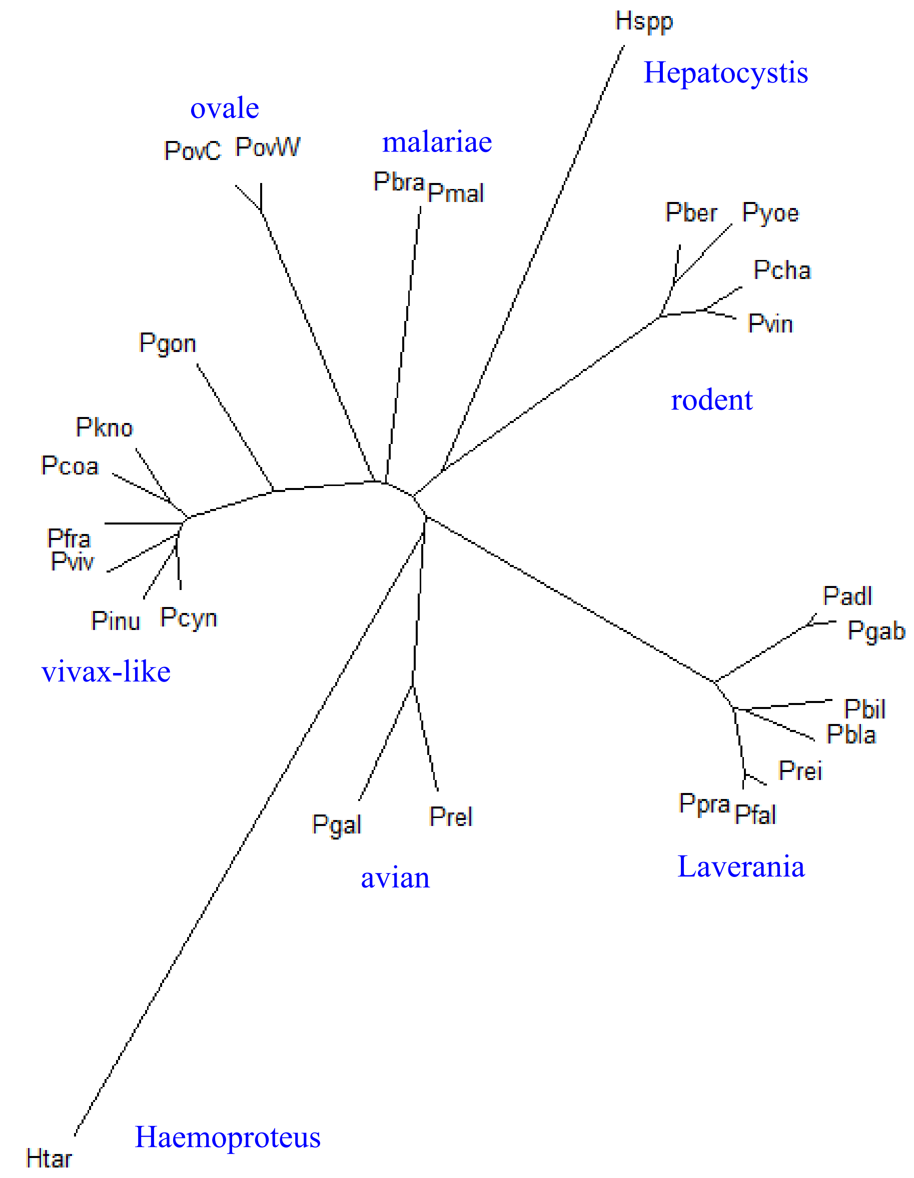
Hspp
Hepatocystis
ovale
PovC PovW
malariae
Pbra Pmal
Pber Pyoe
Pcha
Pvin
rodent
Pgon
Pkno
Pcoa
Pfra
Pviv
Pinu Pcyn
vivax-like
Padl
Pgab
Pbil
Pbla
Prei
Ppra Pfal
Laverania
Pgal
Prel
avian
Htar
Haemoproteus

(c)

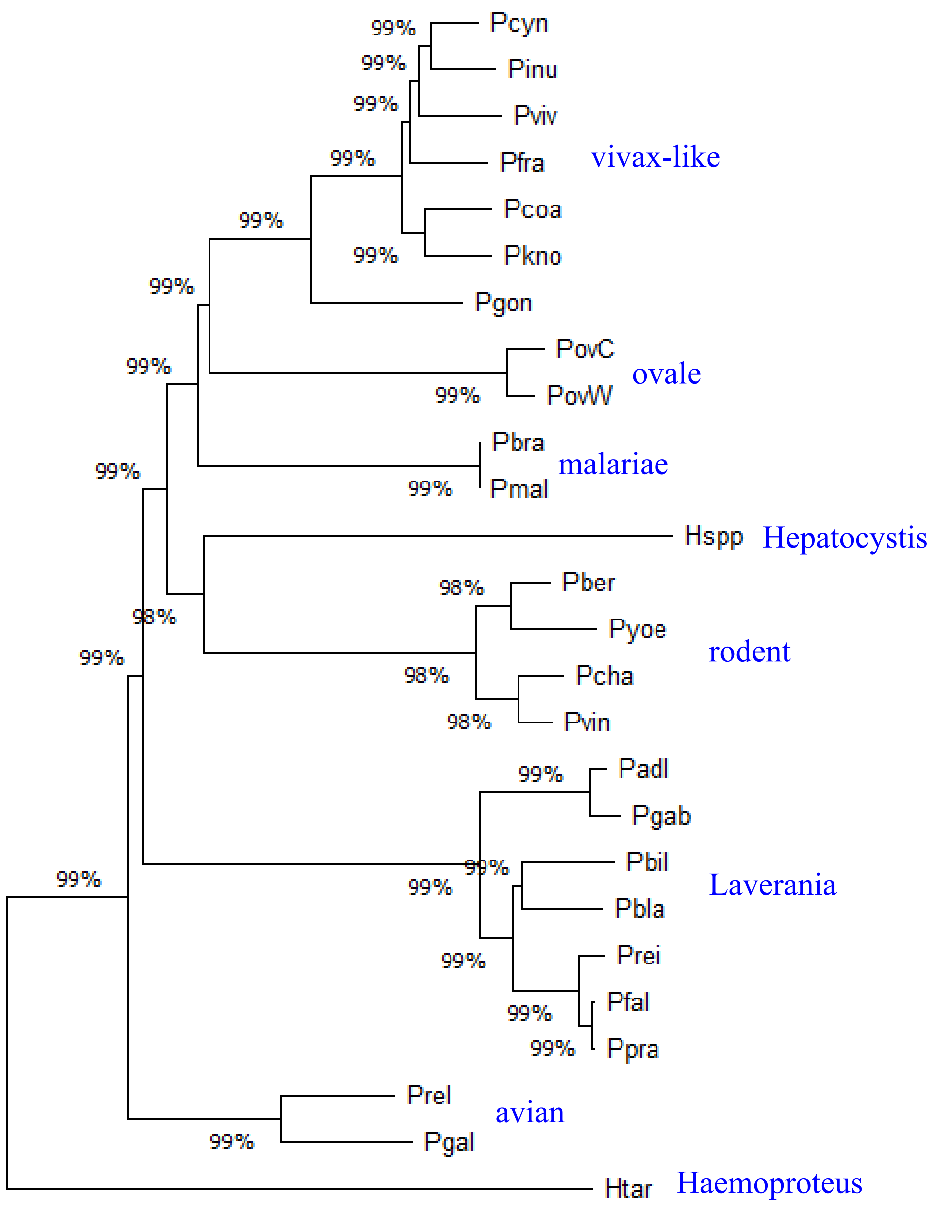
Pcyn
Pinu
Pviv
Pfra
vivax-like
Pcoa
Pkno
Pgon
PovC
PovW
ovale
Pbra
Pmal
malariae
Hspp
Hepatocystis
Pber
Pyoe
Pcha
Pvin
rodent
Padl
Pgab
Pbil
Pbla
Prei
Pfal
Ppra
Laverania
Prel
Pgal
avian
Htar
Haemoproteus
99%
98%
