# Supplementary material for: Homology Modeling of Type-P5 ATPases from the Malaria Parasite: Insight into Their Functions and Evolution, and Implications About the Effect and Role of Intrinsically Disordered Protein Structure
Source: Pathogens. 2025 Nov 14;14(11):1164. doi: 10.3390/pathogens14111164 (PMC12655044; doi:10.3390/pathogens14111164)
Supplement: Supplementary file 1 [file pathogens-14-01164-s001.zip › Supplemental Figure S2.pdf]

Supplemental Figure S2. Alignment of *Plasmodium* type-P5 ATPases with Spf1 (type-P5A) and 13A2 (type-P5B).

```
                                        N-terminal extension (NTE)                                      <-||->      NTD
13A2 --------------------------------------------------------------------------------------------------msadssplvgstptgygtltig
PfA1 ------------------------------------------------------------------------------------------------mykniffphdnikketykvyyyht
PrA1 ------------------------------------------------------------------------------------------------my-nvfdyydkkrkykvlyktilt
PfA3 ---------------------------------marfcksdkllinhrelkyiclfyiillfcfflwcifkkyhenrinrkhkrkgsdsshknekteffnyiyesyndnddviirqegyk
PrA3 ---------------------------------mfrfcksdsllinyrelkyicfiyifllfsffiwclckkhfqnkinkrhkwknhesiyknekaqffnyiyess-dsdeviirqegya
Pf5A mrskyniliykkkkkhlrldvllffvyliflnlilqnkkfeaqpkdyeyieilknknvneetfvhvkrfeptssdlg-nigkekileqnnynvksinnvelrndnmn--vinnegvynky
Pr5A mknkyniiiykkrkthcrldvllflfyafylnfilqnkkfeaqpsdyeyieklklkkseeinilkfnnsnlssdellydeseeeitekldgikirvneleknhinttdknindvnisten
Spf1 -----------------------------------------------------------------------------------------------------------------mtkkSFV
     ^->                                                                                          <-^

              NTD     <-||->           membranes loops of NTD           <-||-> NTD           (IDL)
13A2 tsidplsssvssvrlsgycgspWRVIGYHVVVWMMAGIPLLLFRWKPLWGVRLRLRPCNLAHAETLVIEIRDKEDsswQLFTVQVQTEAIgegslepspqsqae----------------
PfA1 nnlihdeyrisyiecykkkkkkivtilhyvfcilslgllplilswfpifyfrllhsactlnecekvliitkdgnkyikkikkikfnhnvcyvmndvffsedqsmssmyndkeqnknnfny
PrA1 edkniyeyqidYVECYKLKG--IIKIAHYFFCVLSSGLLPLILSWFPIFYFKLLHSKCTLNECEKVLIVTKEGKKYIEKIKNIQLYNDIRVVLNDHYNVHEVSFYSIKS-----------
PfA3 nslcgfllknisivlylllthfiiilligneycikengeilwndrafvffiiflllcfiiitygiltvrkhmhsffikpsilkdsdyvlvytknedytnsyknifkesyvvyitnvfikwnkki
PrA3 nsfvgfvlknisiifylithiiiilliineyciyesndklwndrafvffiifllsfiiitygiltvrkhmhsfflkpsllkdsdyvlvytknedyinfyknifkeileflhiflkkryykl
Pf5A vkeneeiigdnekkekraktfenndfkneksfkpiivgkynliyifysieficlllfiflhfllfllsqwnikinlfisykslysknkekylynlknfcth-------------------
Pr5A ngndinistenngndeqellkende---etqlkpillgkynliyifysiEFVCLLLFIFFHMMVFLLSQWNLNFYLFIAFNNLNCNDKNKYIYNLRNLCTH-------------------
Spf1 SSPIVRDSTLLVPKSLIAKPYVLPFFPLYATFAQLYfqqydryikgpeWTFVYLGTLVSLNILVMLMPAWNVKIKAKFNYSTTKNVNEATHILIYTTPNNG-------------------
                                                                                                             ^->

                                               (IDL)
PfA1 fsrdytfrdqnimnerkdninlkdhiklsghinlkdniklsghinlkdniklsghinlkdniklsghinfkdninfkdsiypcdeilhvdniplh-------------------------
PrA1 ------FCSKNLMN----------------------NCKNSELKNNENDIQKY-YSDIED------------------------------------------------------------
PfA3 ykysckyikllhyyqmnaksffffistdkkkkndyiknsyhdddlddddiqnkdnnnyyysniykknsyynnsfhkksisnqysnkrlsrnslytkkvlrdqennlgyydqdqneinkm
PrA3 kkflfpyfepfnnynk--------------ekkn--------------sifqykknnn---enkyk-------efnkkkisknyen------------ifr-------------------

PfA3 nsidhiynidslnvidqgndknydneekeinkkypfnrndeygdeygdeygdeygnkygnkygnkygnkyddkygnkyddkyddkyddkyddkyddkyddkyddkyddkyddnivwgskr
PrA3 -------------------------eeeiiikk---------------------------------------------------------------------------------------

             (IDL)                                   NTD             <-||-> A-domain
13A2 ---------dgrsqaavgavpegawkdtaqlhkseeavsvgqkRVLRYYLFQGQRYIWIETQQAFYQVSLLDHG----------------------------------------------
PfA1 -----------------nnlninshqpfdetteynksrlqdnnrityIFEFLDIRYVYIKKKNIFVELYFNINY----------------------------------------------
PrA1 -----------------NYEKIKS--------YDEKSELDEYEYSTHIFEFLGVRYVYVRRKKCFVELYFYVTY----------------------------------------------
PfA3 fsnkkkkkgkkkrknemvtsaqtnreyiennlkvhkvkvrinEKNVRYFFFRSMKYVYNEEKDGFYNISHSIEE----------------------------------------------
PrA3 ---ekkkkmetkpin---------------klkvhkvkvrtnEKNIRYFFFRSMKYVYNEKKDAFYNISHSINE----------------------------------------------
Pf5A ---vyiepciikseedngiydknrynmdcnfykpkselielkkidndiyffykqkkyifnyetfifeslkhfdnfnlsfylnwkglldypkgkdinkmdiynnkmmrdikdrvtcnnies
Pr5A ---VYIKPLLKKNDK-AGINSKERFNTDYNFYKPKADLVELKKINNDIFFFYKQKKYLFDYKTVKFESLKHFDIFNLSFYLKWKGLIEYEKEAYDN---IQVNNISNDIKRSIKNEDLNS
Spf1 -----------------------------SDGIVEIQRVTEAGSLQTFFQFQKKRFLWHENEQVFSSPKFLVDE----------------------------------------------
     <-^                                                                       ^->
```

```
                                                                                             (IDL)
Pf5A vikkldilyalhndkkgnlnidekvddvnfncndfiskeknvdnvydmnkknminikyrgktnkgnnnfidvlkndnmcekdinkndifgdynkddmddthnhnvdrniicsnkkkvdsi
Pr5A LFNKIDILYSDKLKKKD------------------IKSYKDKTKVIDSDN----NVKEEGRK-------INRLMNDKNKDEETN----FSEVNKN-------------------SKVHVI

                                         A-domain     <-||->         cTM1            <-|  |->      cTM2      <-||->  A-domain
13A2 -------------------RSCDDVHRSRHGLSLQDQMVRKAIYGPNVISIPVKSYPQLLVDEALNPYYGFQAFSIALWLADHYYWYALCIFLISSISICLSLYKTRKQSQTLRDMVKLSM
PfA1 -------------------PFSYMHLMCTGLRNEKLINDRKILYGECNLNIKSDSFIILLFKEIMNPFFIFQIFAMIVWSLDNYIEYTISILFITSISIILELKNTIKNQKKIKNMLNYTC
PrA1 -------------------PLEYIHLMSDGLNSHKLIRDRKLLYGECNFNIKLDSVFVLLFKEIIHPFFIFQIFAMIIWSIDSYYEYALAILFITAISTIIELRSTRLNQKKLKNMIDYKC
PfA3 ----------------KVKNLDFNYILKKGGLNNNEIINNINEYGYNNIHLEFYSFFKNLKRELLDGIYMFQLFISYKNFFWKEIITSLIWMIISILSVIKKIFKNQKNKKEIYENIQANN
PrA3 ----------------KVNNLDFNYILKKGGLNNNEIINNINEYGYNNIHLEFYSFFKNLKRELLDGIYMFQLFITYKNFFWKEMITSLIWLIISMLTVIKKIFKNQNNKKEIYENIQANN
Pf5A knsihndvvrddnvnnnfynfyykkscfeipydifVHNNVEKYGENIYDIPCPCFKELLYESMLSPFFIFQFFSIVLWMLDSYWYFGIFSIFILIILESQLINKRIREFNMINGMKVDPQ
Pr5A DN---HKIINEN--------FYYNKNYFQIPHDLYVNNNLNKYGENIYDIPSPCFKKLLYESMLSPFFIFQFFSIILWMLDSYWYFGVFSIFILVVLEAQLINKRIREFNLINSMKVPSQ
Spf1 -------------------SPKIGDFQKCKGHSGDLTHLKRLYGENSFDIPIPTFMELFKEHAVAPLFVFQVFCVALWLLDEFWYYSLFNLFMIISMEAAVFQRLTALKEFRTMGIKPY
                  <-^
                                  A-domain                                                  (IDL)
13A2 RVCVCRPGGEEE-WVDSSELVPGDCLVLPQEGG-----LMPCDAALVAGECMVNESSLTGESIPVLKTALPEGLG-------------------------------------------
PfA1 PINVYRYNTSYI--ISSSELVPGDIYEIKNNMT------IPCDTIILSGSVTMSEHMLTGESVPIHKEQLPFEGNAIINKNNKYESNDEKDDYLRIYNNHASINMIKRNHLIEETLGKKD
PrA1 EVKVHRYNSVTV--LDSSNLVPGDVYEIENNMK------IPCDTILLIGSATMSEHMLTGESVPVNKSNLPFID----------EFDDIN--FLKQKKNEKTKLDIVEKHINDIVKSEKK
PfA3 NTFVTVYRNSIVQQIPSNNLTIGDIIIINSKMT------LPCDCLLLTGNVLVDESLLTGESRPMKKICLSSSKANSFNSYHTTSNERNTQNSKNDKYDRYQEGEEEEEYEE--------
PrA3 NTMVTVYRNSIVQQIPSNNLTIGDIIIITSKMT------IPCDCLLLTGNALVDESLLTGESRPMKKICISN--LNSFNSFDKINSNESHILSENQENN---------------------
Pf5A DVYVYRNLRWTI--MKSNKLLPGDIYILTNDMTATDNNICTCETLLIDGTCITDESILTGESVPLIKACIDKSVINNKSNNNNNKNENNNNKNENNNKKKDNNKNENNNKKKDNNKNENN
Pr5A NVYVYRNMQWKI--IKSNMLLPGDIYILSNELNGNDN-ICTCETLLIEGICITDESILTGESVPLIKAAIDK-----------------------------------------------
Spf1 TINVFRNKKWVALQ--TNELLPMDLVSITRTAEES---AIPCDLILLDGSAIVNEAMLSGESTPLLKESIKLRPS--------------------------------------------
                                                                            ^->
                                                                 (IDL)
PfA1 REYKSNTHDLCSMNKLCYINNTYDDVHMKNNKMDYNNNNNNKKKKKINNLNFVKGTYINSNDLLYDDKIGVNIFEDDVNNMKHKFNQRNINYYNKDTNNLEYNNKHRYIYDCLLKKVEAI
PrA1 KKVKKNSN----------INNNYNNKLTKKDEYMIEDWNNK-------NMIHQRNINMNNNYIICEEKSEQTINKYHNEKENMKRRT----YNTNDLNSLLSSPSDSSSTYYERKKKKKK
PfA3 ---------------------------------DHSSIHIDKKKNKKKKNNKKKKSQINNGSKKYVDTQKMDNNKNNIYNKNNIYNNDNNIYNNDNNNNNNNNNIDTYDEGYDHTPFRK
PrA3 ---------------------------------------FIEKQKGNDKKKN-----------------------------------------------------LEYSKINKKDYN
Pf5A NKKKDNNKNNDNNKNNSNNNSYRFIGDDNVERLNYKNFENNENEFIK------------------------------------------------------------DKECDYESSNYC
Pr5A ------------------------------------------------------------------------------------------------------------NTEEKNVESSYE
Spf1 ------------------------------------------------------------------------------------------------------------------------

                                                         A-domain                  <-||->      cTM3      <-|          |-> cTM4
13A2 -------PYCAETHRRHTLFCGTLILQARAYVG---------PHVLAVVTRTGFCTAKGGLVSSILHPRPinfkfyKHSMKFVAALSVLALLGTIYSIFI--LYRNRVPLNEIVIRALDL
PfA1 SQKNKIIYSNEDIN-KYMLYGGTYVLSLYNINKIKYNNK-EENRILGLVIKTGFITTKGKIVNNILYHKKKELNLINDSYKFLIILIIYAFFSVFILLYI--TLSNNEYTNHIIIKCLDI
PrA1 KKKKELERKYEDIISKHTLYAGTTVLSVNSINLTKKKNK-----VLGLVIKTGFITTKGKIINNILYSKKARLNLIQDSYKFLAILAIYALASGIIVLFM--TIINNIFSSNTIIRCLDL
PfA3 QNNDNKKCSVNNFNTNNILYAGTDVISTLNFTEN----------IYAIVINVSIYTYKGKYMQNVLFPNP--LLFKYDSQLPIVF--IFTIMFSFVCLYF--QIRYLGLNMTSIFYTIGT
PrA3 KQHEYNKYNYNNFNTNNILYAGTDVISTLNFSDN----------IYAIVINVSIYTYKGKYMQNVLFPNP--LLFKYDSQLPIVF--IFTIFFSLICLYF--QIYYLGFNMTSIFYSIGT
Pf5A NSLFCNRLDIKNKHKKHIVYAGTNILMTKNENNKFNGKKLPVNGCIGIVLRSGFSTYQGKLVRTIINTSEKVNSSSIDSIIFLMILLFSICSSAYVVYSVLKTNEERNLYKLLLSVSHI
Pr5A NSIFLDRIDIKNKHKKNIIYAGTNILLTKNENNEFNNKKLPINGCVGIVLKNGFTTYQGKLVRTIINTSEKVNSSSIDSIIFLFILLFSISSSAYVVYSVLHSNHERNLYKLILSVSHI
Spf1 ----EDNLQLDGVDKIAVLHGGTKALQVTPPEHKSDIPPPPDGGALAIVTKTGFETSQGSLVRVMIYSAERvsvDNKEALMFILFLLIFAVIASWYVWVEGTKMG--RIQSKLILDCILI
  <-^
```

```
                 cTM4 <-||->              P-domain             <-||-> N-domain                  (IDL)
13A2  VTVVVPPALPAAMTVCTLYAQSRLRR-QGIFCIHPLRINLGGKLQLVCFDKTGTLTEDGLDVMGVVPLKG--------------------------------------------------
PfA1  ITDAIPPALPTTLTVGISIAISRLKKKFSISCLCPHKINIAGQINTMVFDKTGTLTENNLQFIGIITQNKKNKNMLSDFIHIKEMNTESYIHSKDDNMIHNKNSIISEYYIKDNMKNLHT
PrA1  ITDAVPPALPTTLTVGITIAVSRLKKKFSISCISPSRINLAGQINTMVFDKTGTLTENRLDFLGVIPNKNRE---LNDFIPISNYGLVPDLN--ACNFVRTQN--------NNNVSEIVH
PfA3  LSQILPVWTPVVLNIGLNISTNRLKKEKDICCIAPSRIPICGKIRVFFFDKTGTLTDHKIEFSGVHFCNNILNEKKKSISNSVNNIYPNKLLSDDDLKQIKNQTSLLNVSKTLKSIFTKN
PrA3  LSQILPVWTPVVLNIGLNISTNRLKKEKNVCCIAPSRIPICGKIRIFFFDKTGTLTDHKIELSGVHFCNNILNEKKMHSRHSN---HNKSISDDTYDIKFENFSLLN-SKNFKSFYKN-
Pf5A  ITAVIPPEFPITLSLAVTISIVYLYNMKIYCT-EPFRLPFSGKTNICAFDKTGTLTEDNMIVLGLFGLDD--------------------------------------------------
Pr5A  ITAVIPPEFPITLSLAVTISIVYLYNLKIYCT-EPFRLPFSGKSKICAFDKTGTLTEDNMIVLGLFGLDN--------------------------------------------------
Spf1  ITSVVPPELPMELTMAVNSSLAALAKFYVYCT-EPFRIPFAGRIDVCCFDKTGTLTGEDLVFEGLAGISAD--------------------------------------------------
           kkkkk                                   *                     ^->
                                                (IDL)
PfA1  SSKKKSITKERSNFLVQTIKSCLLKDHYIKEKKKEYYTNNTYCNDLHINDSTCSSYLLNSETKDAYCEYYNIDHLCDINKKNMDINSKNELMGKYSKNELMGKYSKNELMGKTIKNELMG
PrA1  LSQKYSFEKKDNNSLLSTIKKCINSNN-VELSFLQYNKN--------IDD----------------CEYHNFDEEIKIKK----IISR-----EY----------------HTLNNGSLN
PfA3  AKSNEKHKQLSKDMKKEAQNKEYQINSVELNNFMHGNHVDILPIENKENNEYNKIHTLDDVTLNHDNVIHHIDSLMPNHDDNNIHAHHNHVLYNNKYNNETSLKSPNGFQSMKSHMPMNK
PrA3  ------------NLKKNWNLRDFDYN---APHFLSNNIVEYYSSNNINNNVFEKINMKDKNDFN-NASQHSFYSSMGFNDANNINEKK------SRNISEEYIQKPLTYYKSHLFALTDN

PfA1  KYSKNELMGKYS------------------------------------------------------------------------------------------------------------
PrA1  NYDDNNNHCNLS------------------------------------------------------------------------------------------------------------
PfA3  NEYNSLQEKREPTHNNDNNNDNNNNDNNNDNNYNYIYAIEDIRMDSMDNTNANDTINDEKDNEIYEEKQNNDVLYNLNEKEQLIDDNNMNEIKKTVYKEHFENYNTNDEPNTEEKINMDN
PrA3  NTYDQPEKNKLFDHKLSN---YNNNERSNDNNS---------------QTSTGDIF------------QSYDELEEYKYYEDEIKEPNHNKKEKNIYRENFKKYNKN-------------

PfA1  -----------------------------------------------------------------------------------------KNELMGKYSKNELMGKYSKNELMGKYSKNEL
PrA1  ------------------------------------------------------------------------------------------------------------------------
PfA3  YEELNNDHNNDHNNDHSNDHNNDHNNDHNNDHNNDLNNDHNNDHNNDHNNDHNNDHNNDHNNDHNNDHNNNNNYNNEFNYNDNHHLQYVGIDGVREDSFVMDQRNIQNDYDEKNGD
PrA3  ------------NNEKKQIQKKKNGRYNNNQTNYKISHDEVEKYNEDTFVNETKENSKKD------------------------------QQVSID--------MNSNIIKGDCFIKKKQ

                                  (IDL)                                                       N-domain
13A2  ----------------------------------------------------------------------QAFLPLVPEPRRLPVGPLLRALATCHALSRLQDTPVGDPMDLKMVESTGW
PfA1  MGKYSKNELMGKTIKNQVGVDTNIYHMNCDNDYNYDYPCDYNCNNCNDTYHRLEYHNINKDNSFNIPPEKNKSYNNISEHIKINYPLLFEALACCHTLSKVNNKIMGDVLEILMFNFTNC
PrA1  ---------------------------NCYNDDRYN-LCNDNSNNYNN------FHFYKKEN--------------FNKIMKMNHSMFHDALACCHSLTKINNELIGDVLEILMFRSTNC
PfA3  DNHTYNTSFDISNVHNNYHNNNNNNNNHVDYVNYDYNNHAYENRNTQNKYFNESNSSMNSITSLLYNSKLMWTNKVTLNNTPSNYHLLIYALAGCSCVYYDNNNIYGNEIDKRLCEATEM
PrA3  KKDLIKS--------NNKRGNDNNNN-----------------IITKTSNINES-TTMNSITSLLYNSKLIWTNKLTLNNTPSNYHLLIYALAGCSCVYYDSNNIYGNEIDKRLFEATEM
Pf5A  ------------------------------------------------------------------NLKRINEINESIINKQKIPFFSLSVIAGCHSICTLNNKLLGDPLEKNSFLKLKC
Pr5A  ------------------------------------------------------------------NFKKINEIKESIVNKQKIPFLSLSVIAGCHSICTVNNQLLGDPLEKNSFLKFKC
Spf1  ----------------------------------------------------------------------SENIRHLYSAAEAPESTILVIGAAHALVKLEDGDIVGDPMEKATLKAVGW
                                                               <-^

                              (IDL)                      N-domain                                  (IDL)
13A2  VLEeepaadsafgtqVLAVMRPPLwepqlqamee-------------pPVPVSVL-HRFPFSSALQRMSVVVA-----------------------------------------------
PfA1  DMLINNNSFIIKEKKKNCSYDFQKIDGDKNIGANDERCHLN------NNLVSYNILKRFEFQSRLQRMSVIVK-----------------------------------------------
PrA1  DIQIGEDCVIIRESE-NLNNNFFSLK-EKNL-----------------LSKPIYYILKQFEFNSNLQRMSVIVK-----------------------------------------------
PfA3  KITN-------YINDDNVNIKKISLKINNNT------------------YKSFDVLKTYEFDYYTKISTTLTYG-----------------------------------------------
PrA3  RISN-------YINKHDMNIKKITLKMN-DE------------------YKSFDILKTYEFDYYTKISTTLSYG-----------------------------------------------
Pf5A  VMKSLNHTYVYTNNINNNNNNNNNINNDNVVDKKYHKNNEKKIKNQSLENFQIVKRFFFSSELQRMTCIILHEGSQHDWYGDEYETDTCDSDEQNEEQYKNTKQHVLRNNGHEIYKTT
Pr5A  NMKCLDNTFVYTNNRKSSNEINKKS----------------GNYYSKNNYVENFQIYKRFFFTSELQRMTCIIAHEGYEYDWYGEEYE-----------EEYE------------------
Spf1  AVERK------------------------------------NSNYREGTGKLDII-RRFQFSSALKRSASIAS-----------------------------------------------
                                                                         ^->
```

```
                                                          (IDL)
13A2 ----------------------------------------------------------------------------------------------------WPGATQPEAYV-KGSPELV
PfA1 --------------------------------------------------------------STYGNNNDDNNDDDNNNDDDNNDDNNNDDNNNDDNNDDNNNNNYYYNIFCKGSPEKI
PrA1 -----------------------------------------------------------------------------------------------------NVQNEKYYLFCKGSPEKI
PfA3 ---------------------------------------------------------------------------------------------------NFDFKEKKYIVFSKGSFDKI
PrA3 ---------------------------------------------------------------------------------------------------YFDFKDKIYIVFSKGSFDKI
Pf5A PYKESAQMLRKLKRKNNNEYDTESDDHADQNCDIHNNDIHNNDIPNNDIHNKDIHNKDIHNKDIRHNDVHHNDICYKHTEKEKKKKNRIKNILFVKKKKENKDKIVKQYLVVSKGSPEIM
Pr5A -------------SKEND------DNTISENVEIIDN-------------------------------------------------------LKKNKNDNSNQIIRQYLVVSKGSPEMM
Spf1 ------------------------------------------------------------------------------------------------------HNDALFAAV-KGAPETI
                                                                                                             <-^

                         N-domain                                   <-||->              P-domain
13A2 AGLCNPETVPTDFAQMLQSYTAAGYRVVALASKPLptvpsleaaqqltrDTVEGDLSLLGLLVMRNLLKPQTTPVIQALRRTRIRAVMVTGDNLQTAVTVARGCGMVAPQEHLIIVHATH
PfA1 KELCLKSKIPNNYDEILNKYTKQGMRILSISYKRVKSKNINLLNVKRSF-VESNLHFLGFLIFTNNMEKNAPD-IIHNLQTSGCQCIMSTGDNVLTSIHVAKKCGIINSNVESIIIGDVI
PrA1 KELCLKNKIPDNYDDMLHKYAKLGMRIISLSYKELDANNYNVLNITRNS-LEKNLLFLGFLIFANKLKNSAYD-MIKNLKSSGCQCIMSTGDNILTSISIAKQCGIINENIESLIIGDVA
PfA3 YMKCIKNEELYFFKKKEQEYSKNGFYVIALAFKIISNMKHEHILDLPRQLLEKDMNFLSLIMFNNHIKKDASM-VIQTLKGSSIRPVILTGDNAYNCLYVGNKIGLFNNVNFYESFFSLN
PrA3 YQKCIKNDEIFFFKKKEQEYSKNGFYVIALAFKIIWKSSYENILELSREELEKDMNFLSLIMFNNHIKNDAPN-VIQTLKDSSIRPIILTGDNAYNCLYVGNKIGLFSKVDFYDSFFSLN
Pf5A KRFLKKVPEHY--DEVLNSLSIKGYRVLCLAVNILDNNMYKE--NISREEVEKDLYFCGFLTFICPIKVTTPN-YILHIKNAGIKNIMITGDNALTACQVSQDVNIVPKVTCKDILILKM
Pr5A KKFLKKIPENY--DQILNSLSIKGYRVICLAAKVLDSKIINK--SIKREEIEKDLHFCGFLAFICPIKKSTPK-YILDIKRAGIKNIMITGDNALTACQVSQDVNITPSVKIKDILILKL
Spf1 RERLSDIPKNY--DEIYKSFTRSGSRVLALASKSLPKmsqskiDDLNRDDVESELTFNGFLIFHCPLKDDAIE-TIKMLNESSHRSIMITGDNPLTAVHVAKEVGIVFGETLILDRAGKS

13A2 PERGQ------------------------------------------------------------------------------------------------------------------
PfA1 PVVGKNNKQKKKLVWYNHKNDTYLKGHDKTCIDNEFTSIQSQMNSDNICGDNICGDNICGDNIYGDNICGDNIYGDNINGDNINGDNIYGDNINGDNINGDNINTYDNIYGDNYN
PrA1 TV-----KKKKKIVWYVDKNDKIFKNED---IDRNIDNKKKKNNDKDIADSNNNNNNNSKKQIFEN--------GKKNNTSDIAFK-IN------ENLNELCKNKESDRYEEDIMKNNN-
PfA3 MNSNINNKYINNKYINNNNKNNIYLNELNTNNHLSNTKNINS-SIHSNMSNMELNHDEMKHSPKSPASKFLSFENFFKSGKKKNIKNNNKTDNENNYEDDNVMHNQSDYQYDHLLTPGTE
PrA3 INSNIN------SEIKKNDKENVFQ--SNETKFLSFENLIKSNSNEKKLSNSPYILDKYNEQPKK---------------KKKNKSSDKKEIDKGNVRN----KKYVDIKYSYIKS---K
Pf5A NEVISYDLIGEKRTNMINMIDIMTTNNNHTCDTNQSDDVCKNKINNVVKDLRYDHAIFKNDDIKDCIEFLTCLKNKETSVILKNHVENLIRIIEVNYNK---------------------
Pr5A KGNVSTNKR--ILKDIPNEMQHNKKNNTNEEEKENKNDINKKEENESMNENR-KYQTFTNEEKEEVITMLNHFISKR-NINLKNYVEKIIEIIE-NSKN---------------------
Spf1 DDNQLLFRD--------------------------------------------------------------------------------------------------------------
                                             ^->

PfA1 LDYCPTEYHKCTYNNSILYRNNFLYKKENKKDKNYKNISTLYEHRTNDIQFDKLCDILINKDPRNVNIVLTGKAFIFLKKKFYSFHLPYYEECKNIVHYIMKKKHKKIKNIINNHNSNLY
PrA1 ---------KCHDSN--ISKN--LEKGEKKKIEKYP--------RNSRLVFDDLCELLIHRDPRSIGIVISGEAFIFLKKEFERLDLPFYDECEKITYNVMENNSKKKK-------NKIY
PfA3 HLDGFKNNETQKKEKHIYESLDKSQKLNETSKYIPGNAANYTTYSEFNHKYDQHKYDMNNEFSNDENSALIPHHVKEKNKRNKKKRSHIINTLIND-NISDDENNSVYSQSHIFFNEQKL
PrA3 YCNGNCKYESEENTLLLPQNKDEKEKLVSNN----LNQNDYSVKNSLMCKFSNGKNDTYNDAVNYSN-----------NNTSADNYDSINNSIEDDKNIKDKESSILKSNDQIHFMNNKA

                                                                 (IDL)
PfA1 YHYNIIDTFVKRMNKEYMCFNKLLYKIQQKLLYNLIHNLYKKKKYMNN---YYDIDEVHLIGNNNN--------NNKNNSKEKKLPLKNKMKHIRKNE-SNDNITFNTYTSNNIYLSKYK
PrA1 YNSKIIPKYIENLHNKNFCYNKLFYKIQSKLFENLSRGILKKKSLENNKNYMRNKSKDSLIYEKYSGLTNSVHLMNINNSEENGLVCLNNIKNKEENQYVNLKILKNKNKSNFLNIKNYI
PfA3 SISIINEEE--------------------------------------------------------------------------------------------------------------
PrA3 SYYIK------------------------------------------------------------------------------------------------------------------

PfA1 YVHHKN-----------------------YYYPDSCTNLRKKKNSLFYNLKKYIYYEKKKYLQHCLLKHDNYKKVELPRIKDINYSYQMESIKTRNFIHSLS-EQFAIFS----NLILSF
PrA1 KITQKRKIMDALLIKHDSDEKEVLSESLRINSLNNKKSLKKKKLSRFNDLTKNILSYPKPLLSLCIIKEKNICKVNYFLQPKENFILKLVFRCTNYFFNLINKKKKKIFREPEHNHLLEY

PfA1 YIIKNDDNNVYNKN-------------YIYNKNYIYNKNSICNKNYICNKNYIYNKNNIYNKNNIYNKKNILTHAKSVLLSGSSKKFLKFFSNIIRRHKLKE------KKNKKNIKRYKM
PrA1 HSSNEENYKLSKLDSIPENQFKLDIYGNTYKKKNKGGGGKKKNVFFLCNGLNKKKIFFKNKIFNINNEMRIMKRKEKRNLQNSTTFNDVYFYSNRKYKKLKEDYLCYNNNSYMNTLSPRF
```

```
13A2 -------------------------------------------------------------------------PASLEFLPMESPTavngvkdpdqaasyTVEPDPRSRHLALSGPTFGI
PfA1 NHVNNTSKGH---IILNMCTHGFKKDYSSLKNKYR--IVNN---KGYMLKNDNVYDRHMYNLTDMYRGTQYGCSKKKNKNIYMNN--NNNILKNKINRFLEHLLVDKCKRNICHKYTDIK
PrA1 NKEKNSYKDVNSILKFNKYRNNRRENVTCLLDEQKKCIENNENVKNDIYLKSNFYKCKSNFKNSLYYKYTYQMKKKKNKIINKKNIIKNKLLMNNYLRCLKQSLYHECKKGTCYRYSHIK
PfA3 -----------------------------------------------------EQKSDENIIVYGYLLNDELIFVNVHNEKRINKNVVLFKDIYKEIILTGEAYTFIRYHVFQIKTPDED
PrA3 -----------------------------------------------------EEKYEENIIVYGHVLNDELIFINIQNDRKISKNIVLYDDIYKEIILTGEAYRFIRHRIFHIKSNDEE
Pf5A --------------------------------------------------------------------------CSNILYFMNRENKKILPFIHNIEYIKVCSEIFSLCITGDIIDYFLE
Pr5A --------------------------------------------------------------------------INNMLYFINRENKKVLPFIDNKEYIKLCAKLFSLCITGDIIEYFLQ
Spf1 ---------------------------------------------------------------------------------VEETVSIPFDPSKDTFDHSKLFDRYDIAVTGYALNALEG
                                                                                   <-^
                                    P-domain                                                   (IDL)
13A2 IVKHFPKLLPKVLVQGTVFARMAPEQKTELVCELQKLQYCVGMCGDGANDCGALKAADVGISLSQA------------------------------------------------------
PfA1 NIKLSIYEYILRTCTVYARMKPKDKSDLILSLKKLPNNSYVGMCGDGANDCLALSCADIGISLCNNN-----------------------------------------------------
PrA1 NVKLSIYEYILRTCTVYARMKPEYKTQLILSLKNLPHMPFIGMCGDGSNDCGALNCADIGVSLSHN------------------------------------------------------
PfA3 FDSEELEEYKNFLLRIRIFSRLTPNNKIEVIRDFIKFDYISGMCGDGSNDCGALKISHAGLALSNL------------------------------------------------------
PrA3 FNNKELEEYKNFLLRVRIFSRLTPNNKIEVIKDFINFGYISGMCGDGSNDCGALKISHAGLALSNS------------------------------------------------------
Pf5A VYKNNLHIFNELIRGVHIFCRMSPKNKEIIIKTLNKIGYITIMCGDGTNDMAALKAAHVGVSLLSIKISYKNRDGNRKSVLNDDRKSLLNNHNNMRMMNMYGDGRV--------------
Pr5A NTQKNSTLFDELIKRVHIFCRVSPKNKEIIIKTLNKLGNITIMCGDGTNDMAALKAAHVGVSLLSIKIGYKN--SNLSSNVKNNSNYLCNNKYYNNSVYVN-ENYL--------------
Spf1 HSQ-----LRDLLRHTWVYARVSPSQKEFLLNTLKDMGYQTLMCGDGTNDVGALKQAHVGIALLNGTEEGLKKLGEQRRLEGmkmmyikqtefmarwnqpqppvpepiahlfppgpknph
                                                                           arm                                 ^->
                           (IDL)                                                      P-domain <-||->        cTM5         <-|
13A2 ------------------------------------------------------------------EASVVSPFTSSMASIECVPMVIREGRCSLDTSFSVFKYMALYSLTQFISVLILY
PfA1 ------------------------------------------------------------------ESSICSSFTSNKLCLHSIVHILIEGRASLVNSFQLFKFISLYSIMQCSQVLILY
PrA1 ------------------------------------------------------------------ESSICAPFTSDNFYLNSVINILVEGRAALVNSFQLFKFISLYSVMQCSVVLILY
PfA3 ------------------------------------------------------------------DTSVVSPFSSKNENLKSVIDILREGRACLVTSINCYKYMLLYGFMISFIKILLF
PrA3 ------------------------------------------------------------------DTSVVSPFSSRNENLKSVIDILREGRACLVTSINCYKYMLLYGFMISIIKIVLF
Pf5A --KSVYDNLRASYSEARNIINNNSNNLGGINFRRSYEQMKLYNEKKKELDKMLQSLDDSLPLIKLGEASIASPFTYKGNDIKCVKEIISCGRCALSKVIMMYKLMIINSLITAFSVSILT
Pr5A --KTMYDNLEIKNRSTG--MHNNMN-------YKYYEQMKLYNERKKKLENMMQSMDDSLPLIKLGEASIASPFTYKGNDIKCVKEIICCGRCALSKVIMMYKLMIINSLITAFSVSILT
Spf1 ylkaleskgtvitpeirkaveeanskpvevikpnglsekkpadlaslllnsagdaqgdeAPALKLGDASCAAPFTSKLANVSAVTNIIRQGRCALVNTIQMYKILALNCLISAYSLSIIY
                                                                    <-^

              |->     cTM6    <-|                    |->       sTM7       <-|                           |->     sTM8      <-|
13A2 TIN-TNLGDLQFLAIDLVITTTVAVLMSRTGPALVLGRVRPPGALLSVPVLSSLLLQMVLVTGVQLGGYFLTLAQPWFVP------LNRTVAAPDNLPNYENTVVFSLSSFQYLILAAAV
PfA1 SIS-NKLTDNQYIFIDIVTILPLSIFMCWTSASEKLSKNIPIGKLFSFPILISIYGQIIIQLFFVMISLVLLMNLSFYK--YDKNKVMKEKSDDTYLYKAQNTLLYILCSFQNLFMCISL
PrA1 AFS-NNLTDNQFIFTDIFTILPLSIFMSWTSASEKLSSNLPLGKLFCFPVLFSIYGQIIIQLFFIFISLFLLFYQPFYK--NDTNAFPLGDAENKKLICTKNTLIFIISSFQILFICISL
PfA3 MNAHAVMSEYGYLFFDNVILLLLAKSMTLSKPACKLKTQTPTSSIIGAQTILSLLCTLLVNFFFLYSIIFYFLPINNLPSSYQANSSAPKSSWWLMSDNYESFLACIWFCFQIVNSALIL
PrA3 MHAHAVMSEYGYLFFDNVILLLLAKSMTLSKPAHKLKTQTPTSSIIGAQTILSLLCTLLVNFFFLYVIIFQFFYVYNLPTSYHMNSSAPKSSWWLMSDNYESFLTCIWFCFQIVNSAFIL
Pf5A LDG-VKLSDAQTTIISLLYT-CLIVLISKTSPLKNITNYSPPNSLFNFSVIISLLSQIIIHFSILIYGWKLACVYREI--------NYIPDIKGDFIPNLVNTCIYYLIYCINLSIFSCN
Pr5A LDG-VKLSDAQTTIISLLYT-SLIVLISKATPLKNISNYSPPNSLFNISVITSLIFQVFIHFSILIYGWKLSSSYRPP--------DYVPDLKGEFTPNLVNTCIYYLIYCINLSIFSCN
Spf1 MAG-VKFGDGQATVSGLLLSVCFLS-ISRGKPLEKLSKQRPQSGIFNVYIMGSILSQFAVHIATLVYITTEIYKLEPR--------EPQVDLEKEFAPSLLNTGIFIIQLVQQVSTFAVN

              |->       sTM9      <-|                                                  |->        sTM10      <-||->  CTE
13A2 SKGAPFRRPLYTNVPFLVALALLSSVLVGLVLVPG---------------------------------LLQGPLALRNITDTGFKLLLLGLVTLNFVGAFMLESVLDQCLPACLRRLRPK
PfA1 NIKNEWRKSVFTNIAFIIWMSFLLLMNTC-ITFFSSE------------------------IFLVGWIIDLLKQYLSLITFPFYFRIFLFFILFFNFLCSYSYEKYIIKYFEkreiqkky
PrA1 NIKTEWRKSVITNVVYVAWILILILVNIF-ITIFSSH------------------------TCLIGSFVHFLKKYLNLVTFPVYFRFYLSFILIINFICTYYFEKYLIrflekkemkkny
PfA3 TFGGKYRKPIFTNHTFMAYYVLINSFLFY-LTVGGPNRLTCLFRMNCNDEISKITKFKFLELISYSASGLSFYGPHGHNILSTGLKIKFLLLNFINIAVNIFISKYILCESLYNVVRRff
PrA3 TFGGKYRKNIFTNYTFMTYYFLINSFLLY-LTLGGPNKLTCLFRMNCNNEISKTTKFKILDLFSYSASGLSFYGPNGNNILSRNHKIRFLFLNFLNIAVNIFISKYILceklynlvrkff
Pf5A YEGLPFMVPIHKNKEIV-YIFAVNFFFLFVLVMDIFP--------------------------------FLNYFFSLVSFPNIRFKFFFFFLMLVDIFLPYLVTNLFKSLRFYIfhkyqi
Pr5A YEGLPFMTPIHKNKEIV-YIFIVNFIFLFSLVMNIFP--------------------------------FLNHFFSLVSFPNIKLKFLFLFLMVLDIVIPYLISNIIRYARLYffqkyki
Spf1 YQGEPFRENIRSNKGMYYGLLGVTGLALASATEFLP----------------------------------ELNEAMKFVPMTDDFKIKLTLTLLLDFFGSWGVEHFFKFFFMDDKPSDIS
```

```
          CTE
13A2 RASKKRFKQLERELAEQPWPPLpagplr
PfA1 nhnhmssifcspqeldttnvfvtcn---
PrA1 khnhins-fpvpneldt-kvfvtcl---
PfA3 nfqnrkvpi-------------------
PrA3 nfknrkipv-------------------
Pf5A ni--------------------------
Pr5A hl--------------------------
Spf1 VQQVKiask-------------------
```

Legend. Shown is a ClustalW manually adjusted based on the 3-dimensional structures as described in the Methods. Identical residues are shaded in black and similar residues are shaded in gray. The boundaries of the domains are marked with vertical lines (|) and arrowheads (-> <-) above the alignment. Carets (^) and arrowheads (-> <-) below the alignments denote variable regions that were removed (vrr) for additional homology modeling. Intrinsically disordered loops (IDL) are denoted in italics and residues excluded from the homology models are denoted in lower case. The residues making up the ‘kink’ in cTM4 which forms the base of the substrate binding groove are denoted with k’s below the alignment and the phosphorylated aspartate (D) in the highly conserved FDKTGTLT motif is denoted with an * below the alignment. The residues forming the ‘arm’ in type-P5A ATPases are double underlined.
