# Supplementary material for: Homology Modeling of Type-P5 ATPases from the Malaria Parasite: Insight into Their Functions and Evolution, and Implications About the Effect and Role of Intrinsically Disordered Protein Structure
Source: Pathogens. 2025 Nov 14;14(11):1164. doi: 10.3390/pathogens14111164 (PMC12655044; doi:10.3390/pathogens14111164)
Supplement: Supplementary file 1 [file pathogens-14-01164-s001.zip › Supplemental Figure S3.pdf]

Supplemental Figure S3. A-domain structure of type-P5 ATPases. Spf1, ATP13A2, and the *Plasmodium* ATPases were modeled with the Spf1 (6xmu) template (a) or the ATP13A2 template (b) and compared to the experimentally determined Spf1 structure (Spf1*) or ATP13A2 structure (13A2*). The eight β-strands (b1-8) of the distorted jelly roll and the three associated α-helices (h1-3) are denoted. The short β-strand (b6’) in teal and circled is found in the experimentally determined structures but none of the modeled structures.

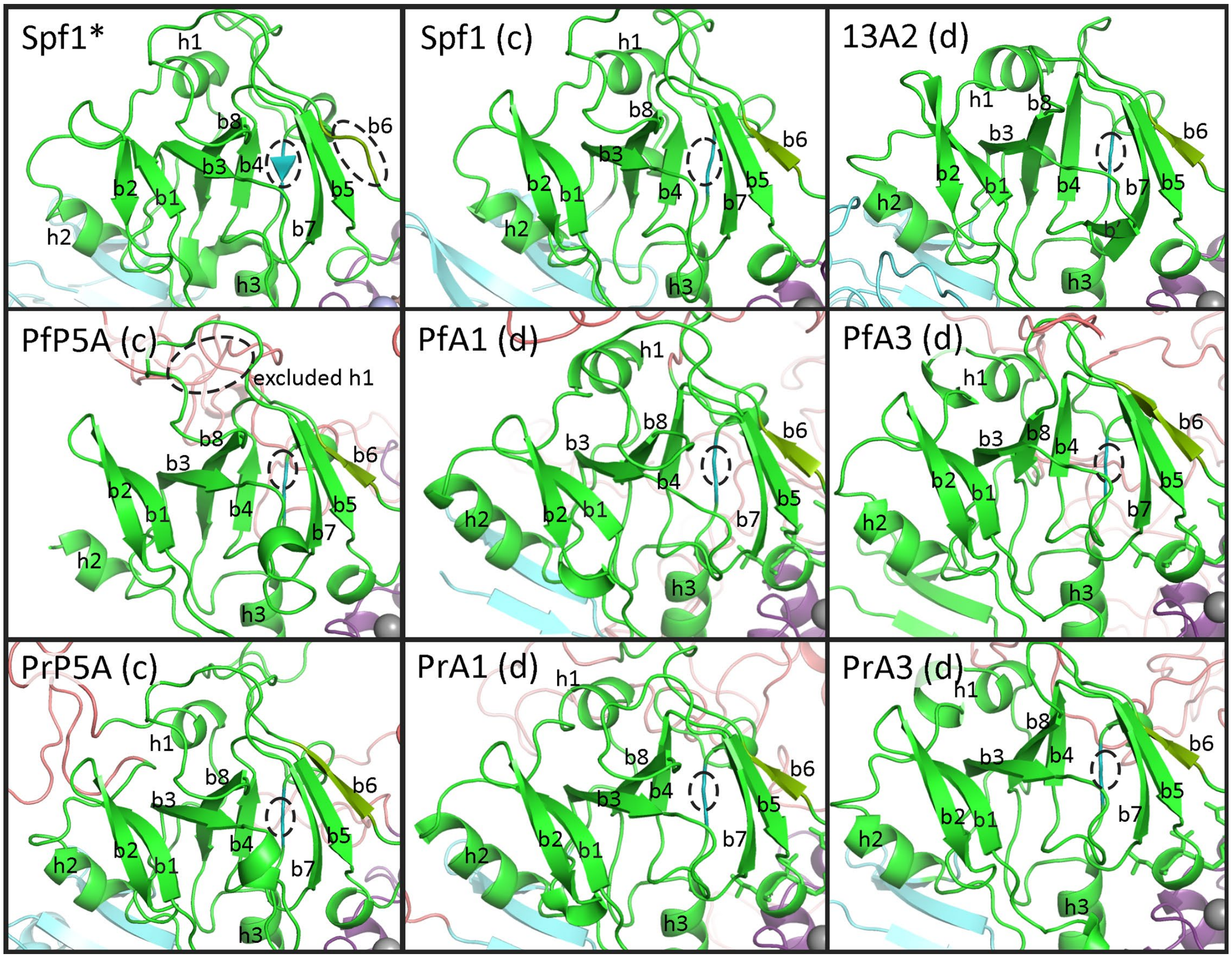

(a) Concordant (c) models are from subtype-P5A sequences, and discordant (d) models are from subtype-P5B sequences. B-strand-6 in ATP13A2 is colored differently as well as the corresponding segment in Spf1 (circled). A large portion of N-terminal sequence including part of the A-domain was excluded from the modeled structure of the *P. falciparum* subtype-P5A (dashed ellipse and excluded h1).

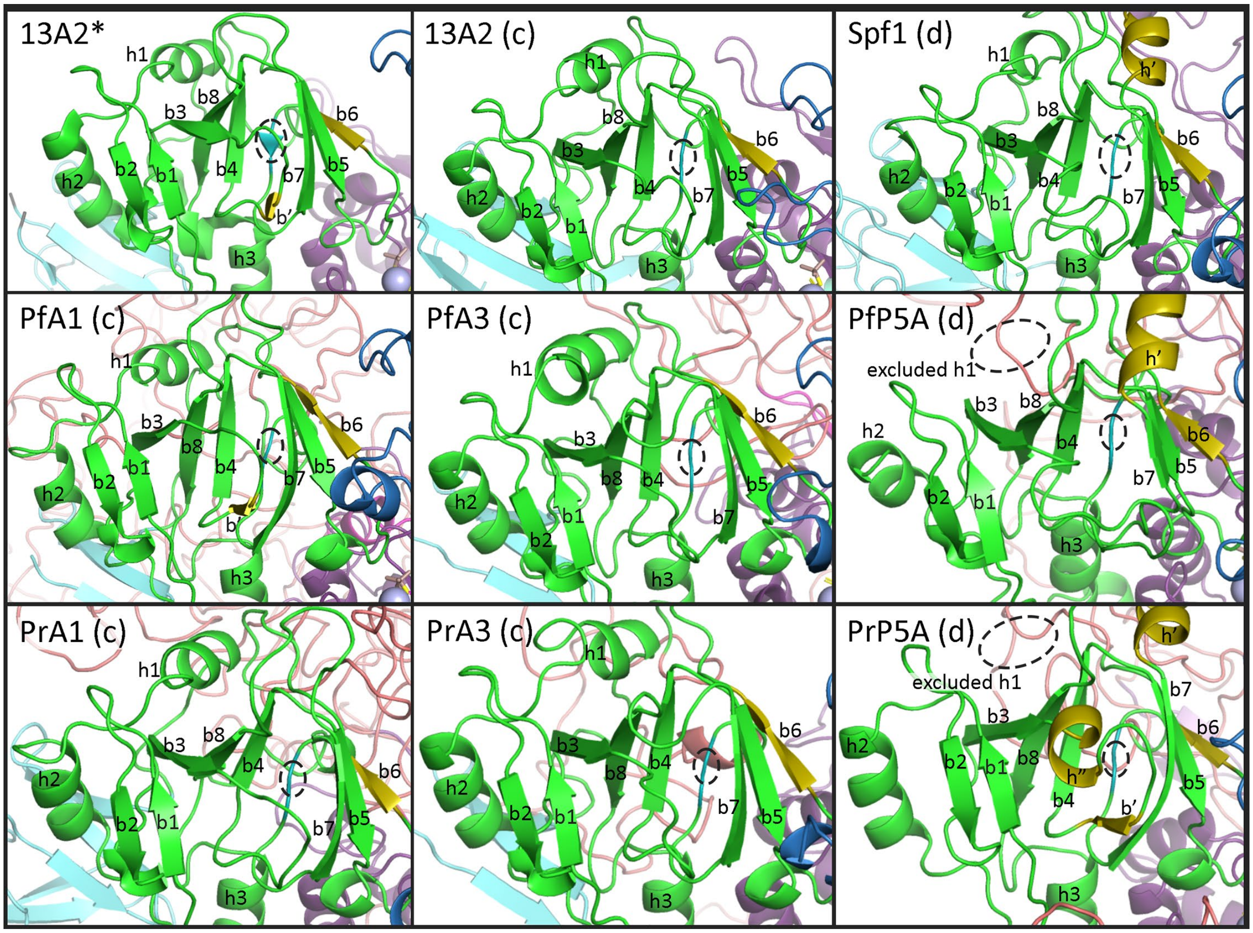

(b) Concordant (c) models are from subtype-P5B sequences, and discordant (d) models are from subtype-P5A sequences. Beta-strand-6 is colored differently since it was missing in the experimentally determined Spf1 structure (a). A large portion of N-terminal sequence including part of the A-domain was excluded from the modeled structure of the *P. falciparum* and *P. relictum* subtype-P5A (dashed ellipse and excluded h1). An extra β-strand (b') and two extra helices (h' and h") were observed in some structures (golden color).
