# Supplementary material for: Homology Modeling of Type-P5 ATPases from the Malaria Parasite: Insight into Their Functions and Evolution, and Implications About the Effect and Role of Intrinsically Disordered Protein Structure
Source: Pathogens. 2025 Nov 14;14(11):1164. doi: 10.3390/pathogens14111164 (PMC12655044; doi:10.3390/pathogens14111164)
Supplement: Supplementary file 1 [file pathogens-14-01164-s001.zip › Supplemental Figure S4.pdf]

Supplemental Figure S4. N-domain structure of type-P5 ATPases. Spf1, ATP13A2, and the *Plasmodium* ATPases were modeled with the Spf1 (6xmu) template (a) or the ATP13A2 template (b) and compared to the experimentally determined Spf1 structure (Spf1*) or ATP13A2 structure (13A2*). The six β-strands (b1-6) and the four associated α-helices (h1-4) are denoted, as well as the hinge that connects the N-domain to the P-domain.

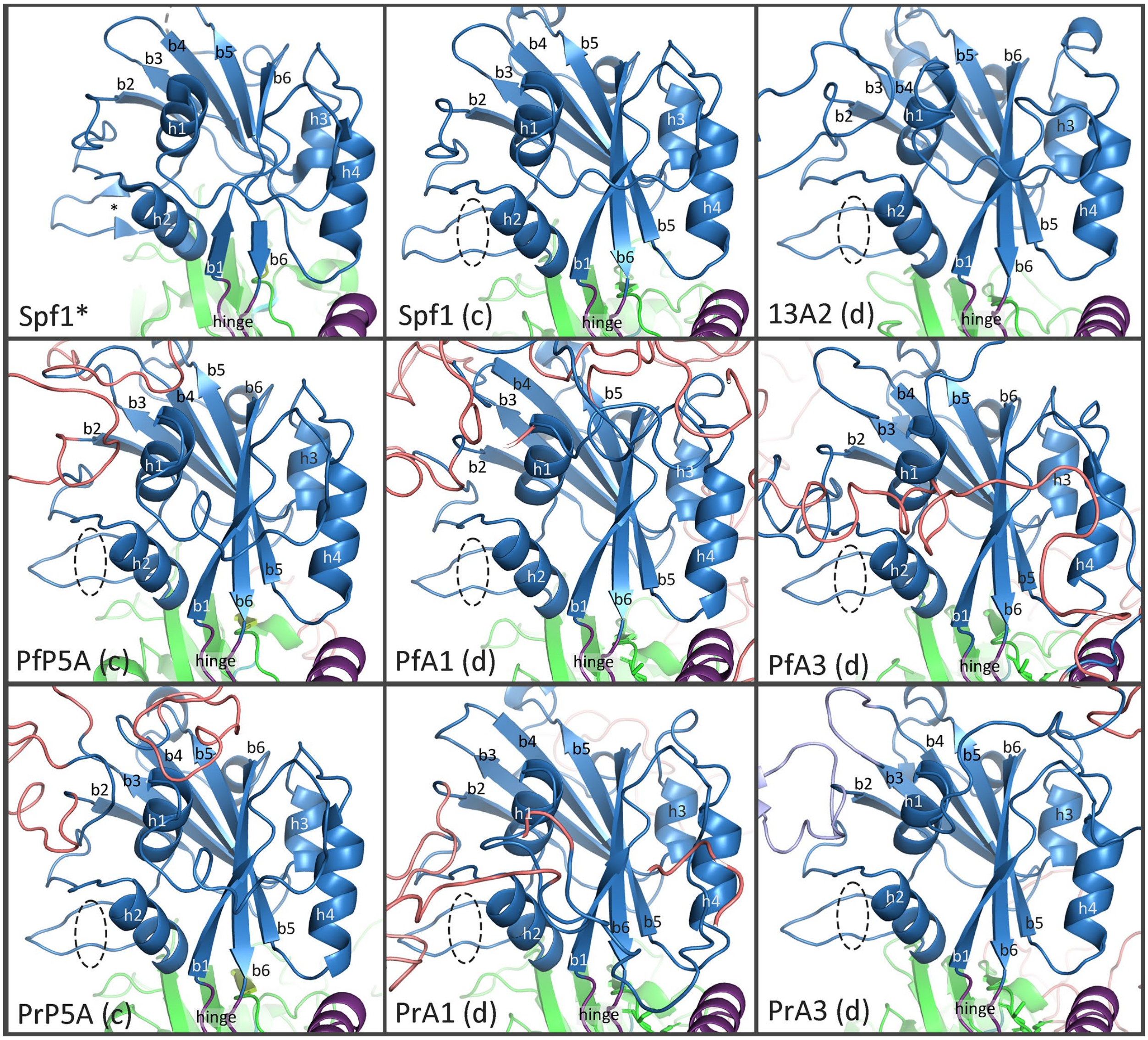

(a) Concordant (c) models are from subtype-P5A sequences, and discordant (d) models are from subtype-P5B sequences. The asterisk (*) in the experimentally determined Spf1 structure denotes two short β-strands that are not found in any of the modeled structures (dashed ellipses).

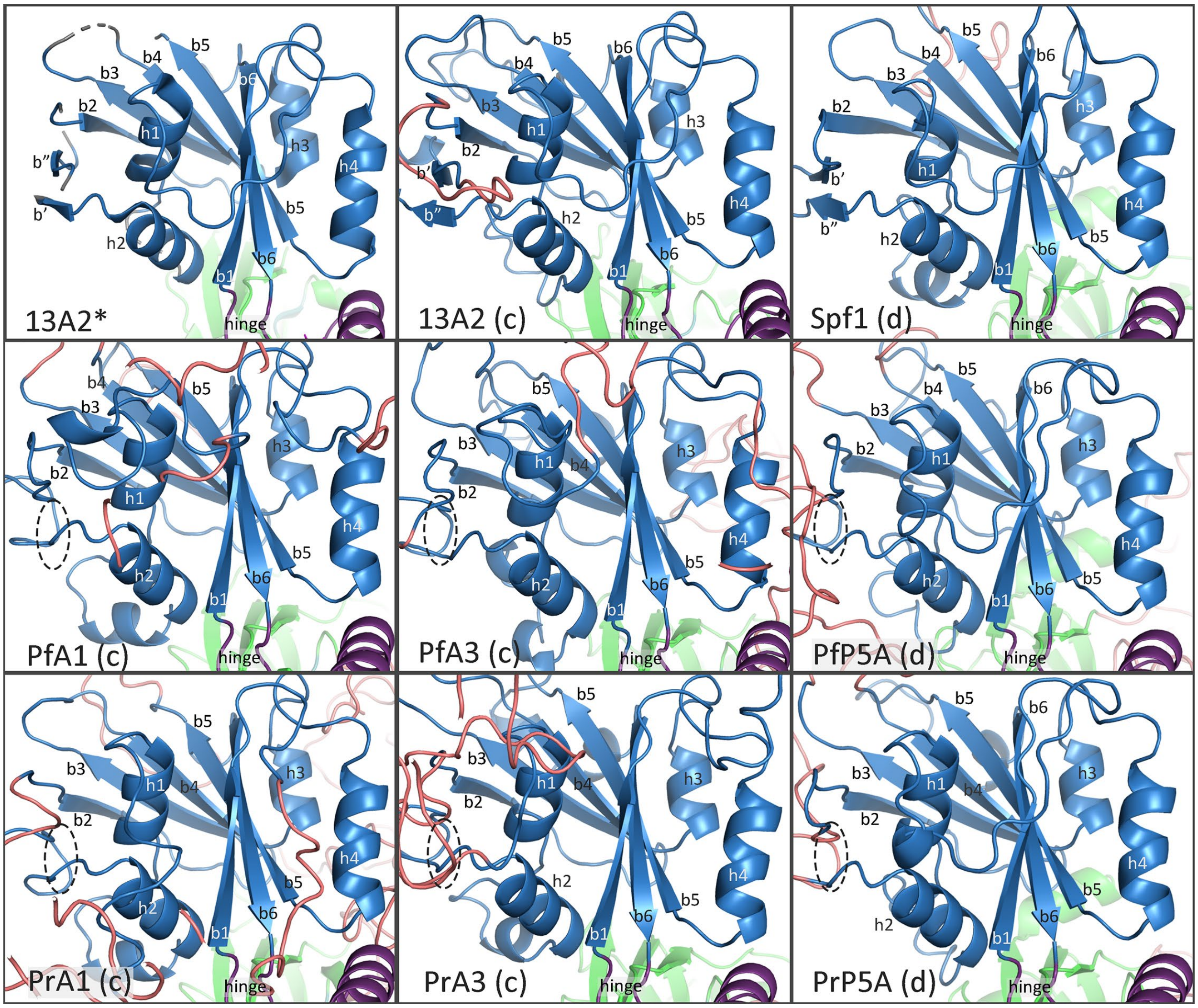

(b) Concordant (c) models are from subtype-P5B sequences, and discordant (d) models are from subtype-P5A sequences. Two additional β-strands (denoted b’ and b”) are observed in the experimentally determined ATP13A2 structure and in the modeled structures of ATP113A2 and Spf1. These extra β-strands are not observed in any of the *Plasmodium* ATPase modeled structures (dashed ellipses).
