# Supplementary material for: Homology Modeling of Type-P5 ATPases from the Malaria Parasite: Insight into Their Functions and Evolution, and Implications About the Effect and Role of Intrinsically Disordered Protein Structure
Source: Pathogens. 2025 Nov 14;14(11):1164. doi: 10.3390/pathogens14111164 (PMC12655044; doi:10.3390/pathogens14111164)
Supplement: Supplementary file 1 [file pathogens-14-01164-s001.zip › Supplemental Figure S5.pdf]

Supplemental Figure S5. Effects of low complexity variable regions on homology modeling.

a) NTD domain

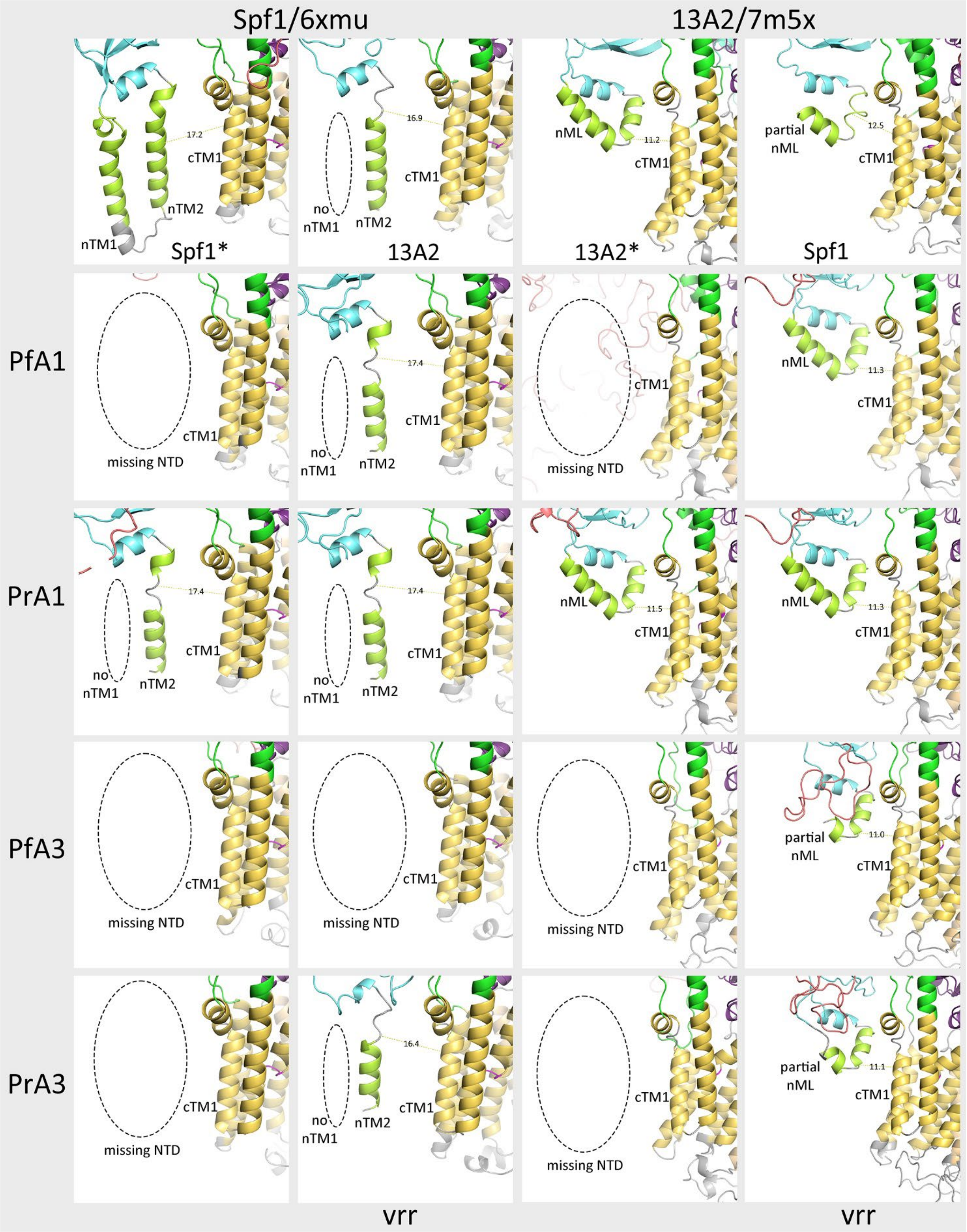

b) A-domain

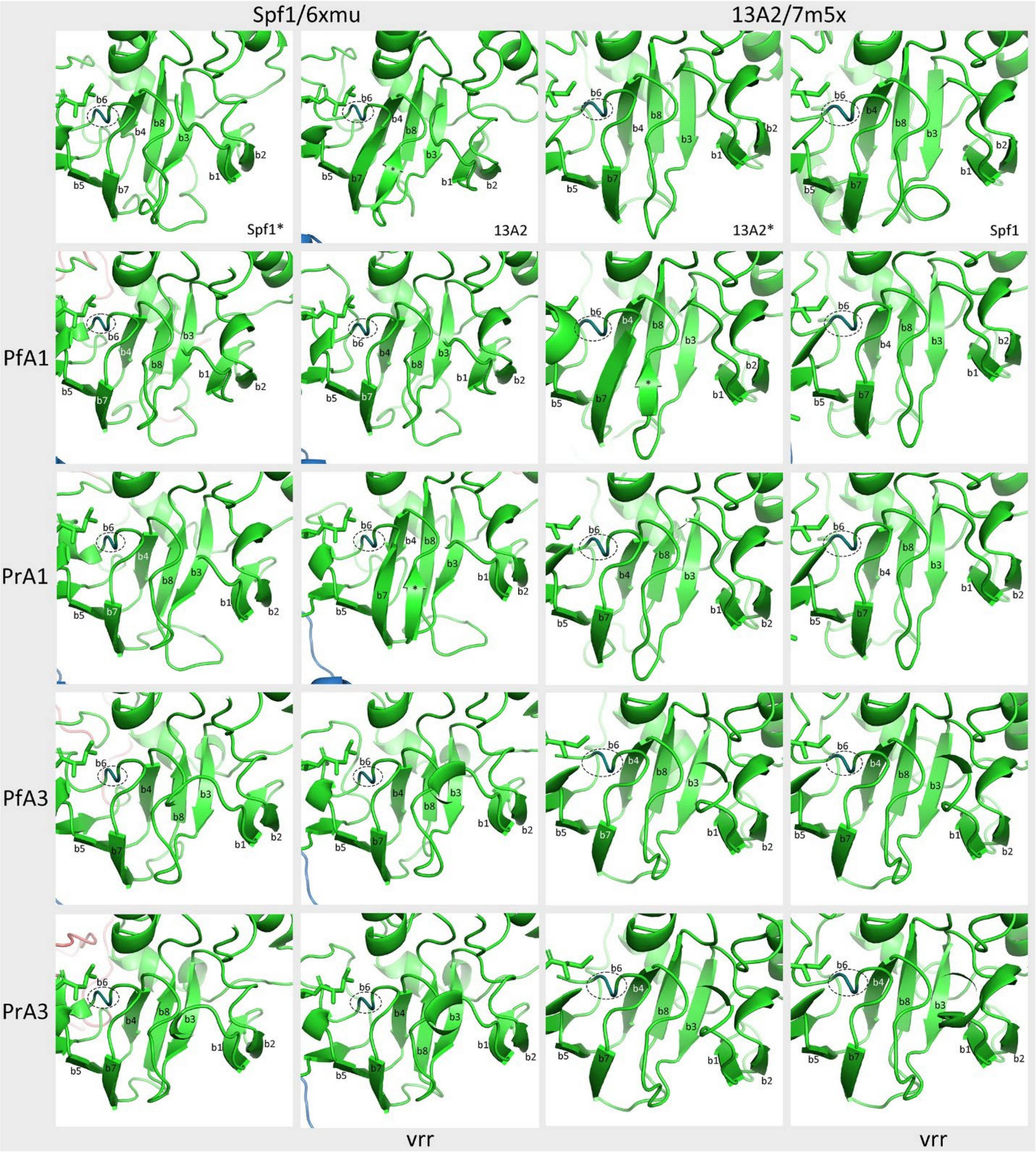
Spf1/6xmu
13A2/7m5x
PfA1
PrA1
PfA3
PrA3
Spf1*
13A2
13A2*
Spf1
vrr
vrr

c) N-domain

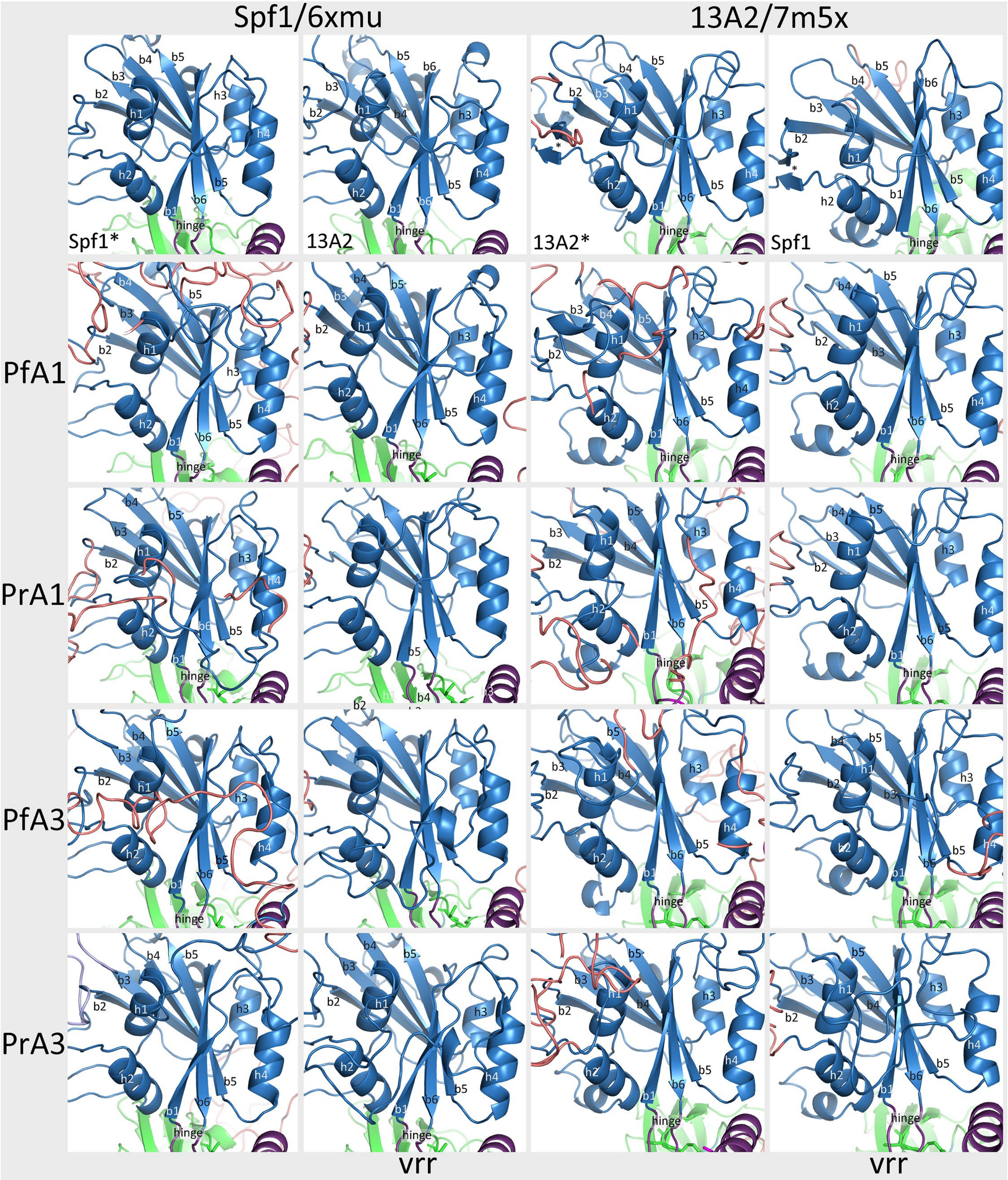
Spf1/6xmu
13A2/7m5x
Spf1*
13A2
13A2*
Spf1
PfA1
PrA1
PfA3
PrA3
vrr
vrr
b1
b2
b3
b4
b5
b6
h1
h2
h3
h4
hinge

d) P-domain

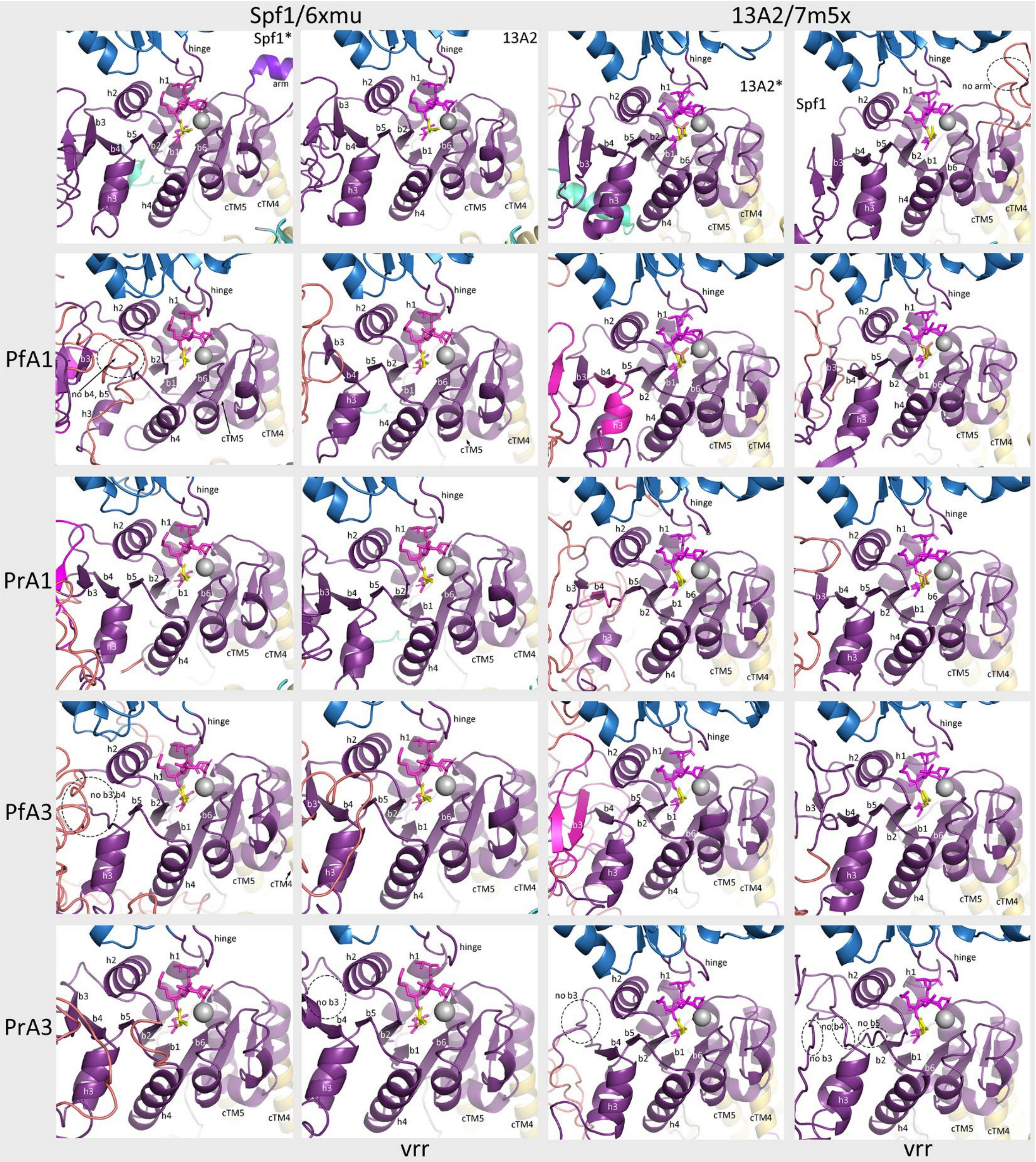
Spf1/6xmu
13A2/7m5x
Spf1*
13A2
13A2*
Spf1
PfA1
PrA1
PfA3
PrA3
hinge
arm
no arm
h1
h2
h3
h4
b1
b2
b3
b4
b5
b6
cTM5
cTM4
no b4, b5
no b3, b4
no b3
no b4
no b5
vrr
vrr

Legend. ATPase1 (A1) and ATPase3 (A3) from *P. falciparum* (Pf) or *P. relictum* (Pr) with or without variable regions (vrr) were modeled with Swiss Model using Spf1 (PDB Acc. No. 6xmu) or ATP13A2 (PDB Acc. No. 7m5x) as templates. The coloring scheme is described in the Methods section. The top rows show Spf1 or ATP13A1 modeled with the concordant (*) or discordant templates. A) A structural feature of the NTD is either two N-terminal transmembrane helices (nTM#) in subtype-P5A or a shorter N-terminal membrane loop (nML) is subtype-P5B. Dashed ovals denote missing elements of the NTD. B) The major structural feature of the A-domain is an eight-stranded (denoted b1-b8 according to sequence order) anti-parallel beta-sheet sometimes called a distorted jelly roll. The two-residues (colored teal and circled) forming beta-strand-6 are not modeled as a beta-strand but are found in the experimentally determined structure. The asterisk (*) denotes an additional beta-strand located between b3 and b4 and is sometimes included in the modelled structure. The highly conserved LTGES sequence is depicted as sticks. C) The N-domains are twisted beta-sheets with six anti-parallel β-strands (numbered b1-b6) flanked by four α-helices (numbered h1-h4). The numbering corresponds to the order of these elements in the primary sequence. The connection between the P-domain and N-domain is often called the hinge. Asterisks (*) denote two additional β-strands found in the Spf1 and 13A2 sequences when modeled with 7m5x, which are not found in the *Plasmodium* sequences when modeled with either template. D) The view is oriented towards the crevice between the N-domain and the P-domain that facilitates ATP binding. The connection between the P-domain and N-domain is often called the hinge and is denoted as such. The FDKTGTLT motif is in stick conformation with the phosphorylated aspartate in yellow. When modeled, BeF_3 is colored salmon. The gray sphere is magnesium. The top row contains Spf1 or ATP13A1 modeled with concordant or discordant templates. P-domains contain a Rossmann fold composed of six parallel beta-strands (b1-b6) forming a beta-sheet and four alpha-helical (h1-h4) segments. The numbering corresponds to the order of these elements in the primary sequence. Missing secondary elements are denoted with dashed ellipses. Secondary structural elements that are incorporated into the P-domain, but that are derived from variable region sequence, are in magenta. The ‘arm’ associated with Spf1 is only generated with the concordant Spf1 sequence and template (bright purple).
