# Supplementary material for: Homology Modeling of Type-P5 ATPases from the Malaria Parasite: Insight into Their Functions and Evolution, and Implications About the Effect and Role of Intrinsically Disordered Protein Structure
Source: Pathogens. 2025 Nov 14;14(11):1164. doi: 10.3390/pathogens14111164 (PMC12655044; doi:10.3390/pathogens14111164)
Supplement: Supplementary file 1 [file pathogens-14-01164-s001.zip › Supplemental Figure S6.pdf]

Supplemental Figure S6. Modeling with AphaFold. Spf1 and ATP13A2 sequences were modeled with Swiss Model (SM) using concordant templates (6xmu or 7m5x) or AlphaFold (AF). *Plasmodium* subtype-P5A (5A), ATPase1 (A1), and ATPase3 (A3) from *P. falciparum* (Pf) or *P. relictum* (Pr) were modeled with AlphaFold (AF). The M-domain (yellow hues), N-terminal domain (NTD, aqua), A-domain (green), N-domain (blue), and P-domain (purple) are denoted when convenient. The IDL are colored salmon.

(a) Modeling of type-P5A ATPases with AlphaFold3.

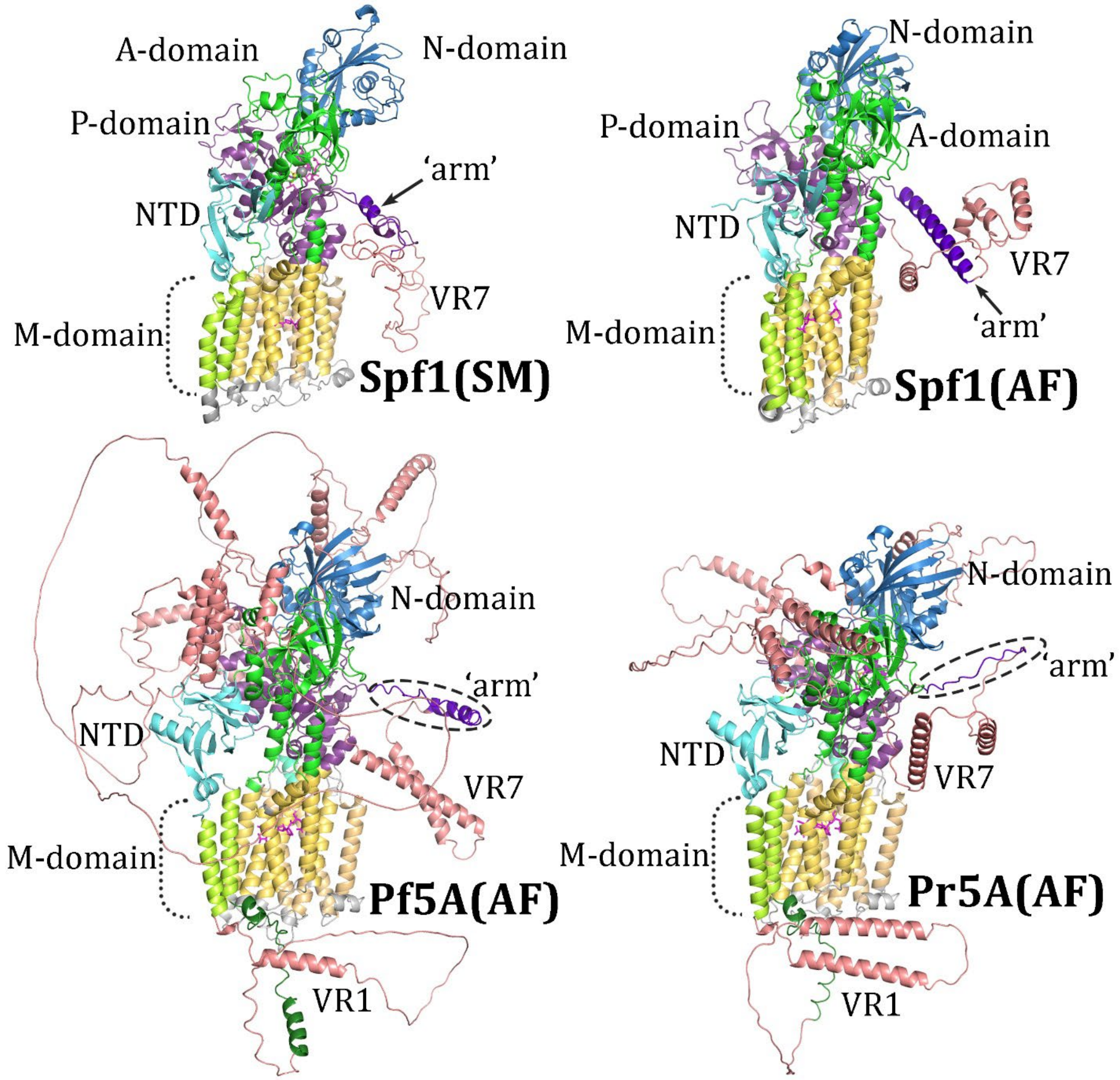

Legend. The IDL are colored salmon and the regions corresponding to the arms are dark purple. Variable region (VR)-1 is located between the N-terminal extension and the NTD. VR7 forms an IDL at the end of the arm that interacts with the membrane.

(b) Modeling of type-P5B ATPases with AlphaFold3.

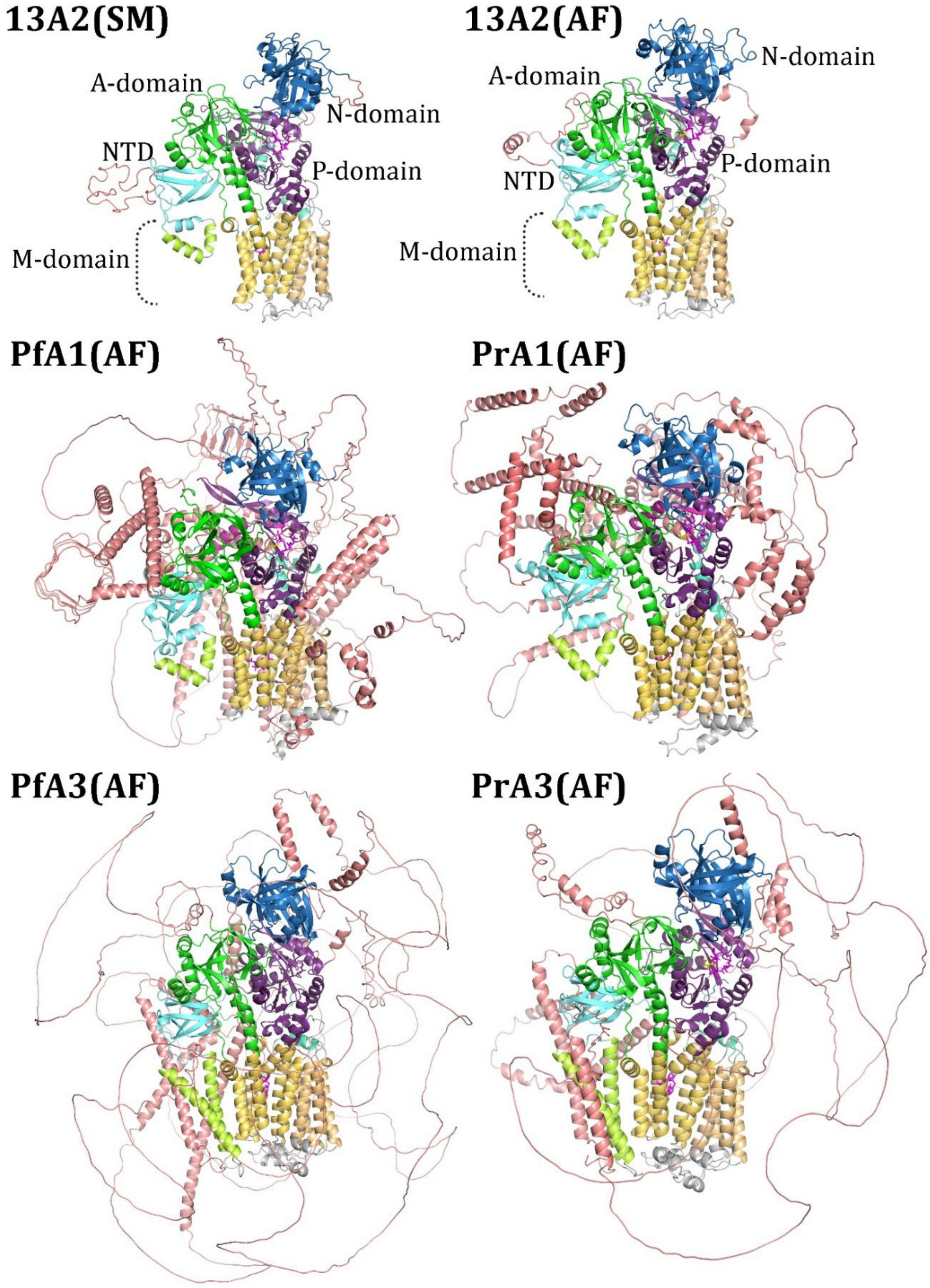

(c) Modeling of the M-domain from type-P5A ATPases with AlphaFold3.

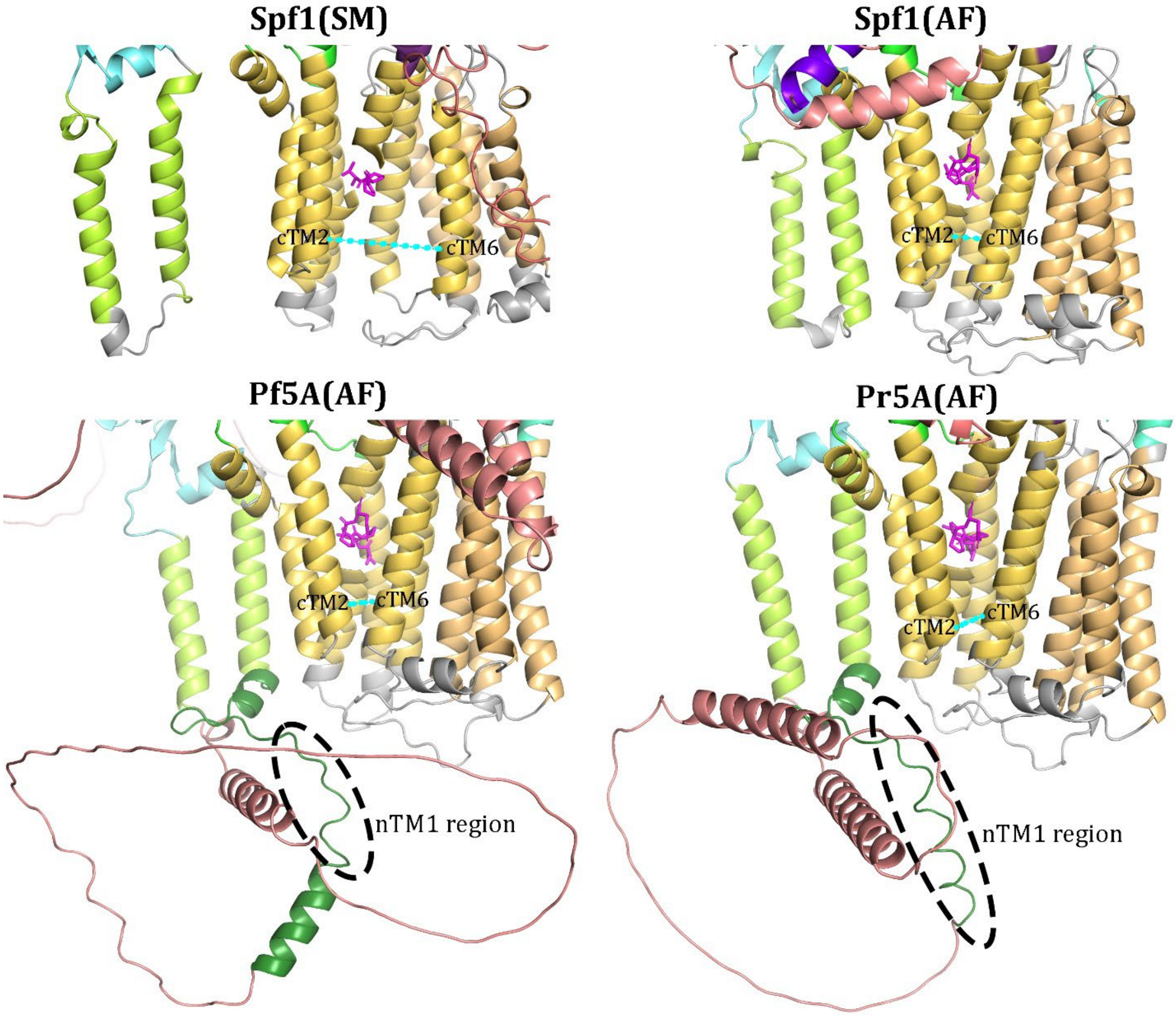

The regions corresponding to the nTM1 transmembrane helix in the alignment are in dark green and circled with dashed ellipses. Distances between cTM2 and cTM6 forming the substrate-binding groove are denoted with a dashed aqua line. The kink of the substrate-binding site is in stick conformation and colored magenta.

(d) Type P5B M-domain

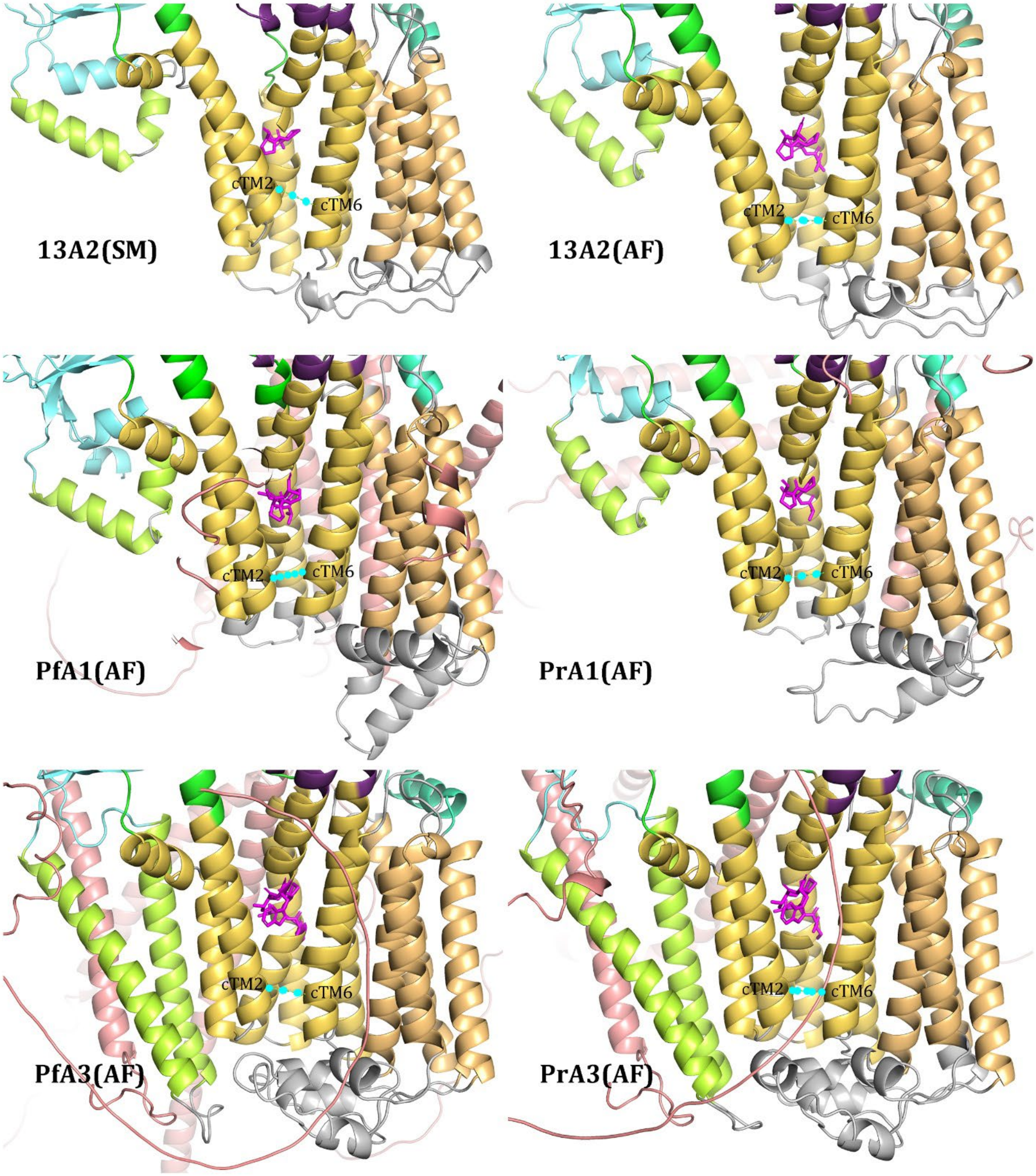

(e) Modeling of the P-domain with AlphaFold.

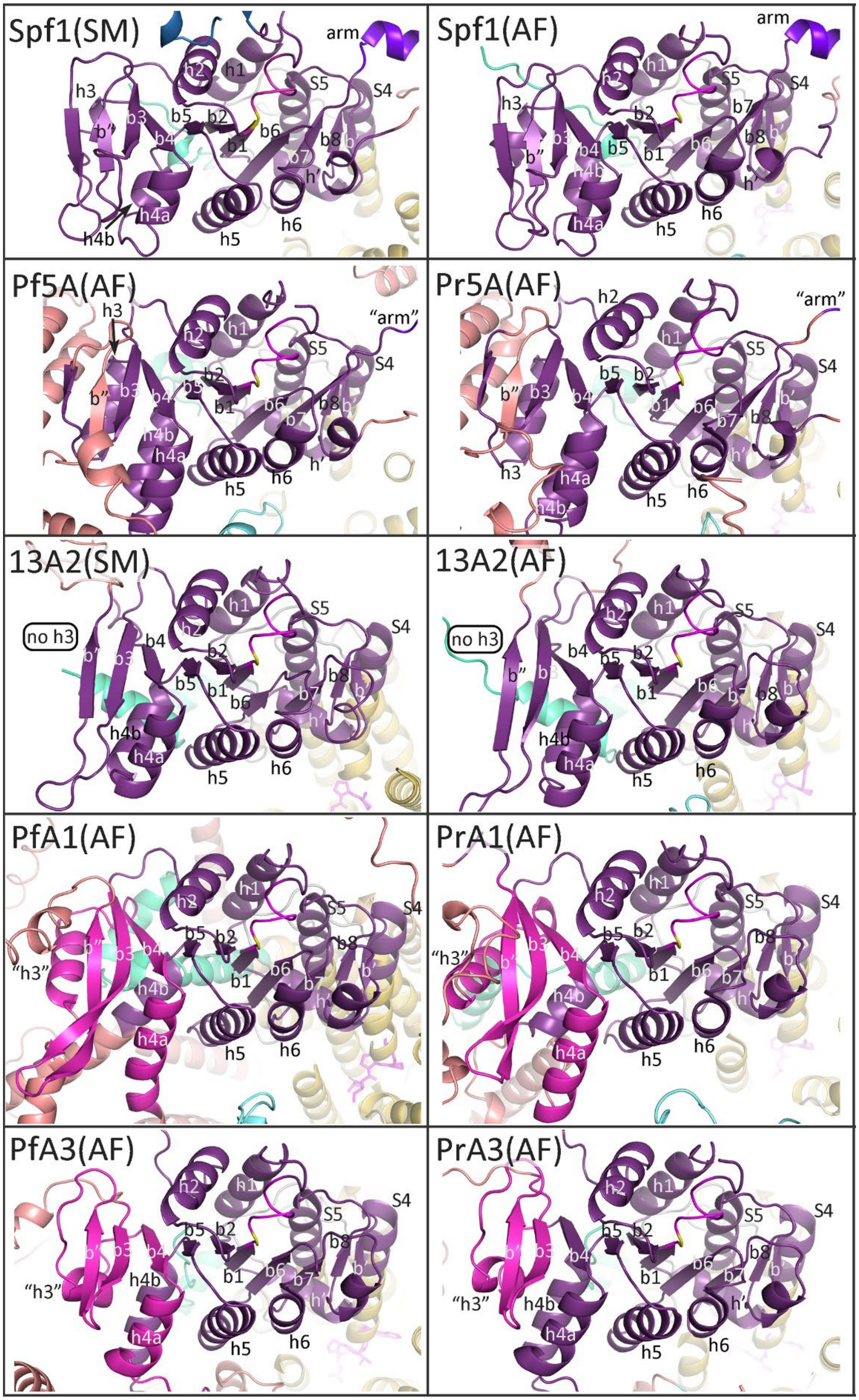

Eight parallel β-strands (b1-8) form a wavy β-sheet that is flanked by two anti-parallel β-strands (b' and b"). The α-helices include two helices that continue from cTM4 (S4) and cTM5 (S5) and form stalks (S), six helices of the modified Rossmann fold (h1-6), and an additional helix (h'). The no h3 box highlights that helix-3 is not found in ATP13A2. Secondary elements derived from variable regions are magenta. Quotation marks ("") around an element indicate that the element is missing or different than the same element in Spf1 or ATP13A2.
