# Supplementary material for: Homology Modeling of Type-P5 ATPases from the Malaria Parasite: Insight into Their Functions and Evolution, and Implications About the Effect and Role of Intrinsically Disordered Protein Structure
Source: Pathogens. 2025 Nov 14;14(11):1164. doi: 10.3390/pathogens14111164 (PMC12655044; doi:10.3390/pathogens14111164)
Supplement: Supplementary file 1 [file pathogens-14-01164-s001.zip › Supplemental Table S1.pdf]

Supplemental Table S1. Identification of type-P5A ATPase genes in SAR (Stramenopiles, Alveolata, and Rhizaria).

**Species**	**Strain**	**Abbr**[a]	**Clade**[b]	**Api Taxon**[c]	**SAR Taxon**[d]	**Gene ID**	**protein ID**	**Chr**[e]	**cd07543**	**TIGR01657**
Plasmodium falciparum	3D7	PfalP5A	Lavernia	Haemosporidian	Apicomplexan	PF3D7_0727800	CAD51021.1	7	9.10E-177	8.03E-149
Plasmodium adleri	G01	PadlP5A	Lavernia	Haemosporidian	Apicomplexan	PADL01_0725500	SOV13227.1	7	6.83E-166	4.01E-147
Plasmodium billcollinsi	G01	PbilP5A	Lavernia	Haemosporidian	Apicomplexan	PBILCG01_0528900	CAC9696051.1		0.00E+00	4.66E-156
Plasmodium blacklocki	G01	PblaP5A	Lavernia	Haemosporidian	Apicomplexan	PBLACG01_0724500	SOV75222.1	7	2.02E-176	1.26E-150
Plasmodium gaboni	SY75	PgabP5A	Lavernia	Haemosporidian	Apicomplexan	PGSY75_0727800	XP_018642614.1	7	2.18E-160	1.56E-138
Plasmodium praefalciparum	G01	PpraP5A	Lavernia	Haemosporidian	Apicomplexan	PPRFG01_0727000	SOS77829.1	7	2.27E-179	1.48E-151
Plasmodium reichnowi	CDC	PreiP5A	Lavernia	Haemosporidian	Apicomplexan	PRCDC_0724600	CDO63730.1	7	1.17E-177	1.58E-154
Plasmodium gallinaceum	8A	PgalP5A	Avian	Haemosporidian	Apicomplexan	PGAL8A_00097100	CRG93265.1		0.00E+00	1.02E-176
Plasmodium relictum	SGS1-like	PrelP5A	Avian	Haemosporidian	Apicomplexan	PRELSG_0216200	CRH03136.1	2	0.00E+00	2.94E-176
Haemoproteus tartakovskyi	SISKIN1	HtarP5A[g]	Haemoproteus	Haemosporidian	Apicomplexan	LSRZ01000006.1			0.00E+00	1.26E-176
Hepatocystis sp	2019	HsppP5A	Hepatocystis	Haemosporidian	Apicomplexan	HEP_00161800	VVF15158.1		2.65E-170	4.24E-153
Plasmodium berghei	ANKA	PberP5A	Rodent	Haemosporidian	Apicomplexan	PBANKA_0211900	VUC53993.1	2	0.00E+00	1.47E-175
Plasmodium yoelii	17X	PyoeP5A	Rodent	Haemosporidian	Apicomplexan	PY17X_0213300	CDU16147.1	2	0.00E+00	4.00E-173
Plasmodium chabaudi	chabaudi	PchaP5A	Rodent	Haemosporidian	Apicomplexan	PCHAS_0210300	VTZ66881.1	2	0.00E+00	3.47E-177
Plasmodium vinckei	CY	PvinP5A	Rodent	Haemosporidian	Apicomplexan	PVVCY_0201160	VEV54678.1	2	0.00E+00	1.37E-175
Plasmodium gonderi		PgonP5A	Vivax-like	Haemosporidian	Apicomplexan	PGO_021320	XP_028541751.1	2	0.00E+00	2.04E-177
Plasmodium vivax	P01	PvivP5A	Vivax-like	Haemosporidian	Apicomplexan	PVP01_0213500	VUZ93367.1	2	0.00E+00	0.00E+00
Plasmodium coatneyi	Hackeri	PcoaP5A	Vivax-like	Haemosporidian	Apicomplexan	PCOAH_00002720	XP_019912738.1	2	0.00E+00	0.00E+00
Plasmodium cynomolgi	M	PcynP5A	Vivax-like	Haemosporidian	Apicomplexan	PcyM_0217000	PlasmoDB	2	0.00E+00	2.23E-179
Plasmodium fragile	nilgiri	PfraP5A	Vivax-like	Haemosporidian	Apicomplexan	AK88_01122	XP_012334183.1		0.00E+00	1.71E-179
Plasmodium inui	San Antonio 1	PinuP5A	Vivax-like	Haemosporidian	Apicomplexan	C922_04283	XP_008818090.1		0.00E+00	1.96E-178
Plasmodium knowlesi	H	PknoP5A	Vivax-like	Haemosporidian	Apicomplexan	PKNH_0212400	CAA9986390.1	2	0.00E+00	1.54E-179
Plasmodium brasilianum	Bolivian 1	PbraP5A	Malariae	Haemosporidian	Apicomplexan	MKS88_000744	XP_067075526.1	2	0.00E+00	8.12E-176
Plasmodium malariae	UG01	PmalP5A	Malariae	Haemosporidian	Apicomplexan	PmUG01_02022400	SBT87178.1	2	0.00E+00	9.28E-176
Plasmodium ovale curtisi	GH01	PovCP5A	Ovale	Haemosporidian	Apicomplexan	PocGH01_02020700	SCA48290.1	2	0.00E+00	0.00E+00
Plasmodium ovale wallikeri	PowCR01	PovWP5A	Ovale	Haemosporidian	Apicomplexan	POWCR01_020015300	SBT75358.1	2	0.00E+00	0.00E+00
Toxoplasma gondii		TgonP5A		Coccidian	Apicomplexan		ABN55907.1		0.00E+00	0.00E+00
Neospora caninum	Liverpool	NcanP5A		Coccidian	Apicomplexan	BN1204_011090	CEL65253.1		0.00E+00	0.00E+00
Besnoitia besnoiti	Bb-Ger1	BbesP5A		Coccidian	Apicomplexan	BESB_044850	XP_029220302.1	3	0.00E+00	0.00E+00
Cystoisospora suis	Wien 1	CsuiP5A		Coccidian	Apicomplexan	CSUI_000700	XP_067927094.1		0.00E+00	0.00E+00
Sarcocystis calchasi	Giessen16	ScalP5A		Coccidian	Apicomplexan	SCGI_LOCUS7592	CAL7864645.1		1.01E-178	4.82E-142
Vitrella brassicaformis	CCMP3155	VbraP5A		Colpodelid	Apicomplexan	Vbra_13145	CEM01515.1		0.00E+00	0.00E+00
Gregarina niphandrodes		GnipP5A		Gregarine	Apicomplexan	GNI_088950	XP_011130755.1		0.00E+00	0.00E+00
Porospora cf. gigantea		PgigP5A		Gregarine	Apicomplexan	KVP17_003450	XP_068378178.1		0.00E+00	0.00E+00
Cryptosporidium parvum	isolate 11730	CparP5A		Cryptosporidium	Apicomplexan	CPHLJ_6g720	KAL5367943.1		0.00E+00	0.00E+00

Cryptosporidium hominis	isolate 30976	ChomP5A	Cryptosporidium	Apicomplexan	GY17_00000070	PPS97767.1		0.00E+00	0.00E+00
Cryptosporidium meleagridis	UKMEL1	CmelP5A	Cryptosporidium	Apicomplexan	CmeUKMEL1_05085	POM82976.1		0.00E+00	0.00E+00
Cryptosporidium ubiquitum	isolate 39726	CubiP5A	Cryptosporidium	Apicomplexan	cubi_02198	XP_028874331.1		0.00E+00	0.00E+00
Cryptosporidium canis	isolate 45460	CcanP5A	Cryptosporidium	Apicomplexan		KAJ1605071.1		0.00E+00	0.00E+00
Cryptosporidium bovis	isolate 52996	CbovP5A	Cryptosporidium	Apicomplexan	FG379_000095	XP_067183049.1		0.00E+00	0.00E+00
Cryptosporidium xiaoi	isolate 45016	CxiaP5A	Cryptosporidium	Apicomplexan		KAK6590325.1		0.00E+00	0.00E+00
Cryptosporidium serpentis	isolate 8845_47	CserP5A	Cryptosporidium	Apicomplexan	ACR3K2_29980	KAL7066570.1		0.00E+00	0.00E+00
Cryptosporidium andersoni	isolate 30847	CandP5A	Cryptosporidium	Apicomplexan	cand_024250	XP_067068696.1		0.00E+00	0.00E+00
Babesia ovata	Miyake	BovaP5A	Piroplasmid	Apicomplexan	BOVATA_013940	XP_028866144.1		0.00E+00	0.00E+00
Babesia microti	RI	BmicP5A	Piroplasmid	Apicomplexan	BMR1_03g04820	XP_012649572.1		0.00E+00	0.00E+00
Babesia sp.	Xinjiang	BspXP5A	Piroplasmid	Apicomplexan	BXIN_0762	XP_028871806.1		0.00E+00	0.00E+00
Babesia ovis	Selcuk	BoviP5A	Piroplasmid	Apicomplexan	BaOVIS_029530	GFE55549.1		0.00E+00	0.00E+00
Babesia divergens	1802A	BdivP5A	Piroplasmid	Apicomplexan	X943_001071	KAK1934348.1		0.00E+00	0.00E+00
Babesia gibsoni	Azabu	BgibP5A	Piroplasmid	Apicomplexan	BgAZ_403130	KAK1442283.1		0.00E+00	0.00E+00
Babesia bovis	T2Bo	PbovP5A	Piroplasmid	Apicomplexan	BBOV_IV002730	XP_001609438.1	4	0.00E+00	0.00E+00
Theileria annulata	Ankara C9	TannP5A	Piroplasmid	Apicomplexan	TA04200	XP_955061.1	3	7.72E-117	1.27E-108
Theileria equi	WA	TequP5A	Piroplasmid	Apicomplexan	BEWA_014370	XP_004832330.1	4	1.36E-148	5.29E-176
Theileria parva	Muguga	TparP5A	Piroplasmid	Apicomplexan	cta4	XP_763395.1	3	8.02E-123	2.37E-108
Paramecium tetraurelia	d4-2	PtetP5A		Ciliate	GSPATT00008912001	XP_001439867.1		0.00E+00	0.00E+00
Paramecium sonneborni		PsonP5A		Ciliate		CAD8050315.1		0.00E+00	0.00E+00
Paramecium octaurelia		PoctP5A		Ciliate	POCTA_138.1.T0360361	CAD8159396.1		0.00E+00	0.00E+00
Paramecium pentaurelia		PpenP5A		Ciliate	PPENT_87.1.T0120490	CAD8143959.1		0.00E+00	0.00E+00
Paramecium primaurelia		PpriP5A		Ciliate	PPRIM_AZ9-3.1.T0100012	CAD8045757.1		0.00E+00	0.00E+00
Ichthyophthirius multifiliis	G5	ImulP5A		Ciliate	IMG5_160550	XP_004030454.1		0.00E+00	0.00E+00
Pseudocohnilembus persalinus	36N120E	PperP5A		Ciliate	PPERSA_11700	KRW98783.1		0.00E+00	0.00E+00
Tetrahymena utriculariae	A2B2	TutrP5A		Ciliate	ABPG72_010529	KAL4481376.1		0.00E+00	0.00E+00
Tetrahymena malaccensis	23b	TmalP5A		Ciliate	ABPG74_010874	KAL4433179.1		0.00E+00	0.00E+00
Tetrahymena thermophila	SB210	TtheP5A		Ciliate	TTHERM_00696910	XP_001025603.2		0.00E+00	0.00E+00
Blepharisma stoltei	ATCC 30299	BstoP5A		Ciliate	CC_MIC31870	CAG9322754.1		0.00E+00	0.00E+00
Halteria grandinella	QDHG01	HgraP5A		Ciliate	FGO68_gene4763	TNV74392.1		0.00E+00	0.00E+00
Stentor coeruleus	WM001	ScoeP5A		Ciliate	SteCoe_22079	OMJ78186.1		0.00E+00	0.00E+00
Moneuplotes crassus	DP1	McraP5A		Ciliate	ECRASSUSDP1_LOCUS11284	CAI2369978.1		0.00E+00	0.00E+00
Stylonychia lemnae	130c	SlemP5A		Ciliate	STYLEM_6775	CDW77809.1		0.00E+00	0.00E+00
Durusdinium trenchii		DtreP5A2		Dinoflagellate	SCF082_LOCUS41914	CAK9088734.1		0.00E+00	0.00E+00
Symbiodinium natans		SnatP5A3		Dinoflagellate	SNAT2548_LOCUS21615	CAE7396985.1		0.00E+00	0.00E+00
Symbiodinium sp.	KB8			Dinoflagellate		CAE7948004.1		0.00E+00	0.00E+00
Symbiodinium sp.	CCMP2592	Ssp2P5A2		Dinoflagellate		CAE7764273.1		0.00E+00	0.00E+00
Amoebophrya sp.	A120	AspAP5A		Dinoflagellate	AMSPA120_T00003953001	CAD7959303.1		0.00E+00	0.00E+00

Prorocentrum cordatum		PcorP5A2	Dinoflagellate	PCOR1329_LOCUS78374	CAK0901433.1	0.00E+00	0.00E+00
Polarella glacialis		PglaP5A	Dinoflagellate	PGLA1383_LOCUS50672	CAE8635067.1	0.00E+00	0.00E+00
Durusdinium trenchii		DtreP5A1	Dinoflagellate	SCF082_LOCUS26714	CAK9047768.1	0.00E+00	0.00E+00
Effrenium voratum		EvorP5A1	Dinoflagellate	EVOR1521_LOCUS24439	CAJ1401253.1	0.00E+00	0.00E+00
Effrenium voratum		EvorP5A2	Dinoflagellate	EVOR1521_LOCUS31641	CAJ1410917.1	0.00E+00	0.00E+00
Cladocopium goreaui		CgorP5A1	Dinoflagellate	C1SCF055_LOCUS31750	CAL1159452.1	0.00E+00	0.00E+00
Cladocopium goreaui			Dinoflagellate	C1SCF055_LOCUS33993	CAL1161938.1	5.84E-149	3.58E-164
Cladocopium goreaui			Dinoflagellate	C1SCF055_LOCUS21977	CAL1148782.1	3.68E-134	4.52E-137
Cladocopium goreaui			Dinoflagellate	C1SCF055_LOCUS21977	CAL1148781.1	6.39E-134	8.80E-136
Cladocopium goreaui		CgorP5A2	Dinoflagellate	C1SCF055_LOCUS35246	CAL1163296.1	0.00E+00	0.00E+00
Cladocopium goreaui			Dinoflagellate	C1SCF055_LOCUS39074	CAL1167525.1	0.00E+00	0.00E+00
Symbiodinium sp.	CCMP2592	Ssp2P5A1	Dinoflagellate	STRI2592_LOCUS823	CAE6928776.1	8.71E-136	1.56E-122
Symbiodinium natans		SnatP5A1	Dinoflagellate	SNAT2548_LOCUS8857	CAE7226706.1	7.36E-121	5.64E-110
Symbiodinium microadriaticum		SmicP5A	Dinoflagellate	SMIC04503_LOCUS5317	CAE7249772.1	1.47E-133	1.86E-119
Symbiodinium microadriaticum			Dinoflagellate		OLP78101.1	5.99E-131	2.95E-117
Symbiodinium necroappetens		SnecP5A	Dinoflagellate	SNEC2469_LOCUS26430	CAE7851597.1	3.44E-134	2.02E-121
Symbiodinium natans		SnatP5A2	Dinoflagellate	SNAT2548_LOCUS17343	CAE7331543.1	0.00E+00	0.00E+00
Symbiodinium microadriaticum			Dinoflagellate	SMIC04503_LOCUS4143	CAE7236878.1	0.00E+00	0.00E+00
Prorocentrum cordatum		PcorP5A1	Dinoflagellate	PCOR1329_LOCUS30843	CAK0833011.1	0.00E+00	0.00E+00
Amoebophrya sp.	A25		Dinoflagellate	AMSPA25_T00014549001	CAD7954445.1	0.00E+00	0.00E+00
Hondaea fermentalgiana		HferP5A	Stramenopile	FCC1311_032862	GBG27063.1	0.00E+00	0.00E+00
Ectocarpus fasciculatus	EfasUO1	EfasP5A	Stramenopile	PHAE_EFASS1_2010.7377.1	CAM9706540.1	0.00E+00	0.00E+00
Pelagophyceae sp.	CCMP2097	Psp7P5A	Stramenopile	M885DRAFT_463980	KAJ1455667.1	0.00E+00	0.00E+00
Chrysophaeum taylorii	NIES-1699	CtayP5A	Stramenopile	CTAYLR_003391	KAJ8599753.1	0.00E+00	0.00E+00
Aureococcus anophagefferens	CCMP1851	AanoP5A	Stramenopile	SO694_000382101	KAK7233254.1	0.00E+00	0.00E+00
Phytophthora nicotianae	CHvinca01	PnicP5A	Stramenopile	L917_12287	ETL88651.1	0.00E+00	0.00E+00
Plasmodiophora brassicae	e3	PbrasP5A	Rhizaria	PBRA_006986	CEO98872.1	0.00E+00	0.00E+00

[a]Abbreviation used in alignments and figures. [b]The clade within the haemosporidians. [c]The sub-taxon of the apicomplexans. [d]The major groups within the SAR taxon including three groups (apicomplexans, dinoflagellates, and ciliates) within the Alveolata. [e]Chromosome number when known of the malaria parasites. [f]E-values of the conserved-domain searches against subtype-P5A (accession number cd07543) and subtype-P5B (accession number TIGR01657). [g]Sequence corresponds to nucleotides 40220-45132 of HtScaffold0006 with an A inserted at nucleotide 40312.
